# Elevating RNA m^5^C methylation provides a promising strategy for crop productivity

**DOI:** 10.1093/nsr/nwag102

**Published:** 2026-02-12

**Authors:** Xiulan Li, Cong Li, Xiangyu Wang, Liwen Yang, Weijun Guo, Yichao Mao, Hanlin Liu, Dongwei Li, Shuangyong Yan, Yong Zhang, Xiaofeng Gu, Li Pu

**Affiliations:** Biotechnology Research Institute, Chinese Academy of Agricultural Sciences, Beijing 100081, China; Biotechnology Research Institute, Chinese Academy of Agricultural Sciences, Beijing 100081, China; Biotechnology Research Institute, Chinese Academy of Agricultural Sciences, Beijing 100081, China; Biotechnology Research Institute, Chinese Academy of Agricultural Sciences, Beijing 100081, China; Biotechnology Research Institute, Chinese Academy of Agricultural Sciences, Beijing 100081, China; Biotechnology Research Institute, Chinese Academy of Agricultural Sciences, Beijing 100081, China; Biotechnology Research Institute, Chinese Academy of Agricultural Sciences, Beijing 100081, China; Biotechnology Research Institute, Chinese Academy of Agricultural Sciences, Beijing 100081, China; Tianjin Key Laboratory of Crop Genetics and Breeding, Tianjin Crop Research Institute, Tianjin Academy of Agricultural Sciences, Tianjin 300384, China; Chongqing Key Laboratory of Plant Resource Conservation and Germplasm Innovation, Integrative Science Center of Germplasm Creation in Western China (Chongqing) Science City, School of Life Sciences, Southwest University, Chongqing 400715, China; Biotechnology Research Institute, Chinese Academy of Agricultural Sciences, Beijing 100081, China; Biotechnology Research Institute, Chinese Academy of Agricultural Sciences, Beijing 100081, China

**Keywords:** RNA m^5^C, demethylase, OsNOP2, grain yield, multiple crops

## Abstract

RNA 5-methylcytidine (m^5^C) has been identified as a key epitranscriptomic modification of mRNAs involved in regulating multiple post-transcriptional processes. Here, we found that knockout of the RNA m^5^C demethylase, OsNOP2, results in elevated m^5^C levels and positively influences numerous agronomic traits in rice. After verifying OsNOP2 RNA m^5^C demethylase function *in vitro* and *in planta*, we found that enhanced m^5^C levels arising from *OsNOP2* knockout results in increased translation, particularly for transcripts involved in carbon assimilation and nitrogen metabolism. *OsNOP2*-KO boosts grain yield by ∼28% per plot in the Nipponbare genetic background in normal conditions. Furthermore, these increased yield traits are maintained under both heat treatment and saline soil conditions. More importantly, knockout of *OsNOP2* in the rice varieties Longgeng31 and Xiushui134, as well as its orthologs in wheat and tomato, also increases the RNA m^5^C level to enhance yield, supporting functional conservation of OsNOP2’s regulatory impacts. Together, our findings unveil an RNA m^5^C-elevating mechanism by OsNOP2 that epigenetically governs carbon assimilation and nitrogen utilization efficiency in plants, providing a potential strategy for genetic improvement in multiple crops.

## INTRODUCTION

Epitranscriptomic chemical modifications of RNAs have emerged as crucial regulatory mechanisms affecting several aspects of RNA metabolism, including mRNA stability, splicing, localization and translation [1–4]. Currently, over 170 different post-transcriptional chemical modifications have been identified among total cellular RNAs, including mRNAs, tRNAs, rRNAs and non-coding RNAs [5–8]. In particular, RNA 5-methylcytosine (m^5^C) modification is an epitranscriptomic mark that is dynamically added or removed by ‘writer’ and ‘eraser’ enzymes, respectively [9,10]. A number of m^5^C regulators have been reported to date, including NOL1/NOP2/Sun domain (NSUN) family RNA m^5^C methyltransferases (RCMTs) in human cells [11,12]. Among them, NOP2 (NSUN1) is an S-adenosyl-l-methionine-dependent methyltransferase that regulates cell cycle and cell proliferation in animals [13]. In animals, known RNA m^5^C demethylases include 11 translocation family (TET2) members and the alkylated DNA repair protein, alkB homolog 1 (ALKBH1) [14]. Recently, a chromatin regulation pathway by TET2 via oxidation of retrotransposon RNA m^5^C has been reported [15].

Homologs of HsNSUN2 have been identified in plants, including NOL1/NOP2/Sun2 (OsNSUN2) in rice [16] and tRNA-specific methyltransferase 4B (AtTRM4B) in *Arabidopsis* [17]. All members of HsNSUN2 homologs consist of a conserved catalytic NSUN domain with two conserved cysteine sites [12,18]. AtTRM4B-dependent m^5^C modifications have been shown to directly affect the transcript abundance of transcripts involved in root development [19,20]. OsNSUN2 has been shown to act as an RNA m^5^C methyltransferase that positively regulates chloroplast function through increasing *OsPAL1* translation levels [16]. Although RNA m^5^C modifications are known to regulate the fate of mRNA, the full scope of their functions has remained elusive. In addition, no m^5^C demethylases have yet been identified to regulate plant agronomic traits.

Here, we identified the NSUN family protein OsNOP2 as an RNA m^5^C demethylase that participates in regulating photosynthetic capacity and nitrogen metabolism in rice. In *OsNOP2*-KO rice lines, both RNA m^5^C levels and translation of hypermethylated transcripts were increased. Moreover, knockout of *OsNOP2* in the Nipponbare genetic background increased rice yield by ∼28% per plot in multi-plot, multi-year field trials, most likely due to increased photosynthetic capacity and nitrogen utilization. At the same time, *OsNOP2*-KO plants maintained obviously increased yield under heat treatment or saline soil conditions compared to wild-type (WT). *OsNOP2* knockout also led to enhanced grain yield and higher total RNA m^5^C levels in the elite rice varieties Longgeng31 (LG31) and Xiushui134 (XS134). Additionally, knocking out NOP2 orthologs boosts grain yield by ∼33% per plant in wheat, and increases fruit numbers per plant (26.4% to 40.2%) and fresh weight per fruit (33.2% to 33.8%) in tomato. Our study thus demonstrates that increasing RNA m^5^C modification levels via disruption of RNA m^5^C demethylase function in plants can confer yield improvements in rice and other crops.

### RESULTS

#### *OsNOP2* locus regulates grain yield

**Figure 1.**
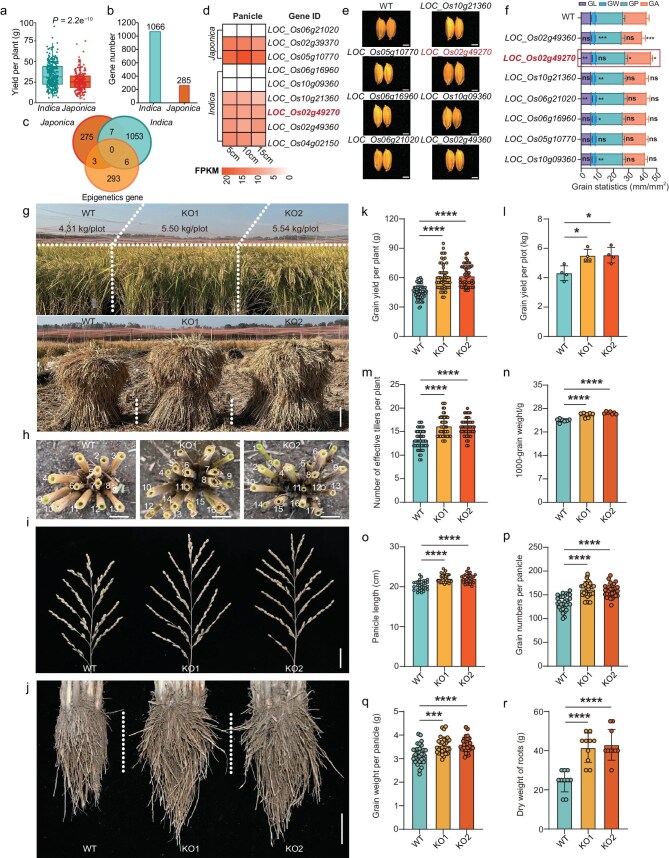
*OsNOP2* knockout boosts grain yield in Nipponbare background. (a) Distribution of *indica* rice and *japonica* rice after yield correlation analysis. (b) Statistics of gene numbers in *indica* and *japonica*. (c) Venn diagram showing overlap of yield-related genes in *indica* and *japonica* and epigenetic genes in rice. (d) The expression of nine genes in *indica* and *japonica* overlapped with epigenetic genes in panicles at different developmental stages. (e and f) The phenotypes (e) and statistics (f) of grain length (GL), grain width (GW), grain area (GA) and grain perimeter (GP) of related gene-editing materials. (g–j) Field morphology, grain yield per plot (g), tillers (h), panicle (i) and root (j) of WT, KO1 and KO2 plants. Scale bars: 10 cm (g), 3 cm (h and j) and 5 cm (i). (k–r) Statistical analysis of grain yield per plant (k), grain yield per plot (l), number of effective tillers per plant (m), 1000-grain weight (n), panicle length (o), grain numbers per panicle (p), grain weight per panicle (q) and dry weight of roots (r) in WT, KO1 and KO2 plants. (g and l) Yields in paddy fields between WT, KO1 and KO2 grown in the 4.5-m^2^ paddies (each paddy contained 10 × 15 plants) were harvested. Data are mean ± SD. (k and m): *n* = 30 plants; (l): *n* = 4 biological replicates; (n): *n* = 7 biological replicates; (o–q) *n* = 30 panicles; (r) *n* = 10 plants. *P < 0.05, ***P < 0.001, ****P < 0.0001; *P* values are from one-way analysis of variance (ANOVA) (and non-parametric or mixed).

To identify and assess genes involved in regulating yield in rice, we first examined previously published yield data for *indica* and *japonica* populations and downloaded the yield phenotype and high-quality SNPs of 529 rice varieties [21,22]. We also used the https://www.elabcaas.cn/rice/index.html website to obtain a list of 302 ‘epigenetic genes’ of rice [23], and compared this list with the published yield-related genes. Among 1066 total yield-related genes in *indica* and 285 genes in *japonica* that we examined (Fig. 1a and b), only 7 genes were common potential co-regulators of yield in both *indica* and *japonica* rice populations, and 9 candidate epigenetic-related yield regulators (Fig. 1c, Table S1). We then performed RNA sequencing (RNA-seq) analyses of panicle tissues sampled at various developmental periods from the *japonica* accession, *Oryza sativa* cv. Nipponbare, and analyzed transcript levels for the nine candidate epigenetic-related yield regulators (Fig. 1d). We then generated and obtained seven homozygous gene-edited materials, and phenotypic screening of the seeds revealed that *OsNOP2 LOC_Os02g49270* exhibited superior traits in grain length, width, perimeter and area (Fig. 1e and f).

To explore potential impacts of OsNOP2 function on grain yield, we generated two independent knockout lines (*OsNOP2*-KO1/2) by CRISPR-Cas9-mediated gene editing in the Nipponbare (WT) genetic background, and obtained the homozygous materials by genotype identification (Fig. S1a). Phenotypically, the *OsNOP2*-KO plants had significantly greater mass for both aerial and root organs (Fig. S1b–d). Analysis of *OsNOP2* relative expression by quantitative real-time polymerase chain reaction (RT-qPCR) in WT plants showed that it was ubiquitously expressed across tissues, including shoot, root, leaf, leaf sheath, stem, shoot apical meristem (SAM), tiller bud and panicles. Further RNA *in situ* hybridization assays showed that a clear signal from *OsNOP2* transcripts could be detected in various parts of the root, shoot apex, leaf and tiller bud (Fig. S2).

Evaluation of field characteristics in the *OsNOP2*-KO lines showed significant increases in yield over that of WT plants at both the individual plant and plot levels, with ∼37% higher yield per plant and 27.7%–28.5% higher yield per plot in multi-plot, multi-year field trials (Fig. 1g, k and l, Tables S2–S7). Moreover, the *OsNOP2-*KO lines had significantly higher biomass increase (∼45.5%) and ∼51.0% greater straw weight than WT, although no significant change was observed in harvest index (Fig. S3). In addition, the number of effective tillers per plant significantly increased (20.5% to 22.0%), as did 1000-grain weight (5.7% to 10.1%) and grain numbers per panicle (20.8% to 21.2%) in the *OsNOP2-*KO lines (Fig. 1h, m, n and p). These improvements in yield were accompanied by significant increases in panicle length (8.22% to 8.37%), primary branch (4.6% to 13.6%), secondary branch (19.6% to 30.5%) and weight per panicle (11.7% to 13.0%) in the *OsNOP2*-KO1/2 lines compared to WT plants (Fig. 1i, o and q, Fig. S4). We also noted that *OsNOP2*-KO plants had significantly larger grain size and faster grain filling rate than WT at the grain filling stage (Fig. S5). These results suggested that knockout of *OsNOP2* affected several yield-related traits that together contributed to higher grain yield.

To assess whether and how the genetic diversity of OsNOP2 might affect yield across diverse rice accessions, we performed haplotype analysis of the *OsNOP2* coding sequence together with evolutionary analysis in the 3K (RG) dataset [24] (Fig. S6a). This analysis identified six haplotypes, among which, Nipponbare carried Hap2. We noted that Hap4 included a frameshift mutation, which introduced an N-terminal premature stop codon that resulted in a truncated (170 amino acid) protein (Fig. S6b). We found Hap4 has higher grain width and 1000-grain weight compared to Hap2, which may be an elite haplotype (Fig. S6c). Notably, *OsNOP2* transcript levels were markedly lower in accessions carrying Hap4 compared with its expression in Nipponbare (Fig. S6e). Further analysis showed that Hap4 accessions had higher grain yield than those carrying Hap2 resulting from improving panicle and grain phenotypes (Fig. S6d, f–l). These data suggested that loss of OsNOP2 function positively regulates yield.

#### *OsNOP2* knockout positively regulates rice growth and development

To better understand the mechanism through which *OsNOP2* knockout might lead to increased grain development and yield, we first conducted morphological and histological analysis of different tissues involved in grain development across growth stages to quantify cellular-level changes associated with loss of OsNOP2 function. At the grain-filling stage, *OsNOP2*-KO plants had significantly increased plant height (8.0% to 17.3%) and internode length compared with WT (Fig. S7). At the seedling stage, *OsNOP2*-KO plants had significantly greater tiller bud numbers per plant (48.6% to 54.3%) (Fig. S8). Further, *OsNOP2*-KO plants had significantly higher (1.7-fold) root dry weights than WT (Fig. 1j and r). Assessment of root morphology showed that *OsNOP2*-KO plants had significant increases in both total root length and the number of lateral roots (2.0-fold and 2.1-fold, respectively), as well as total root area and root volume (1.4-fold and 1.6-fold, respectively) compared to WT (Fig. S9). In addition, propidium iodide (PI) staining and 5-ethyl-2′-deoxyuridine (EdU) staining of root tip meristems from 3-day-old *OsNOP2*-KO seedlings showed that root apical meristems were significantly longer (61.6% to 69.8%), and the number of meristem cells was also greater (56.7% to 63.0%) relative to that in WT roots (Fig. S10a–c). EdU assays to assess cell proliferation confirmed that EdU signal in root meristem was significantly greater in the *OsNOP2*-KO lines compared to WT (Fig. S10d and e), supporting the likelihood that OsNOP2 functioned in suppressing or modulating the root cell proliferation rate.

We then generated OsNOP2 complementation lines (COM) in the *OsNOP2*-KO2 background. Subsequent real-time qPCR analysis of OsNOP2 transcription levels in COM and WT plants showed similar expression levels between groups (Fig. S11). Phenotypic analysis of *OsNOP2*-COM lines showed that the increased plant height and number of effective tillers per plant observed in *OsNOP2*-KO2 plants was fully restored to that of WT. In addition, *OsNOP2*-COM lines had similar grain yields to that of WT (Fig. S12). Additionally, we constructed OsNOP2 overexpression (OE) lines in WT (Nipponbare). Further assessment of yield-related traits in *OsNOP2*-OE lines showed that increasing *OsNOP2* transcription resulted in significantly shorter shoot length and fresh root weight at the tillering stage compared to WT, as well as reduced plant height and number of effective tillers per plant at the grain-filling stage (Fig. S13). Correspondingly, grain yields were also significantly lower in *OsNOP2*-OE plants compared with WT (34.2%–38.1% lower per plant and 29.2%–31.6% lower per plot) (Fig. S14). Taken together, these results indicate that OsNOP2 functions as a negative regulator of grain yield in rice.

#### Knockout of *OsNOP2* maintains increased yield traits under both heat treatment and saline soil conditions

As OsNSUN2 has been shown to positively regulate heat tolerance in rice [11,16], we next examined the yield under heat treatment conditions. WT and *OsNOP2*-KO plants were treated with high temperature in the paddy field at the booting stage (Fig. S15a and b), and we observed that *OsNOP2*-KO lines exhibited larger panicles, more grain numbers, higher seed setting rate and higher 1000-grain weight, leading to 38.6%–40.4% higher yield per plant and 23.6%–24.0% higher grain yield per plot than WT under heat stress conditions (Fig. S15c–i). Further evaluation of yield traits under high salt conditions indicated that *OsNOP2*-KO lines exhibited larger panicles, more grain numbers, higher seed setting rate and higher 1000-grain weight under salt stress conditions (3.8‰ NaCl) compared to WT in Dongying (Fig. S16a–e). Moreover, in field tests with 2.5‰ NaCl soil conditions conducted in Tianjin, *OsNOP2*-KO2 plants showed markedly stronger phenotype than WT at both the tillering and ripping stages (Fig. S16f and g). In the harvest stage, the *OsNOP2*-KO2 line had significantly higher plant height, number of effective tillers per plant, and 1000-grain weight, resulting in an ∼36.1% increase in grain yield per plot under these saline soil field conditions (Fig. S16h–k). These results further supported the notion that *OsNOP2* knockout could maintain high grain yield under heat and salt stress conditions.

#### *OsNOP2*-KO enhances photosynthetic capacity and nitrogen utilization

**Figure 2.**
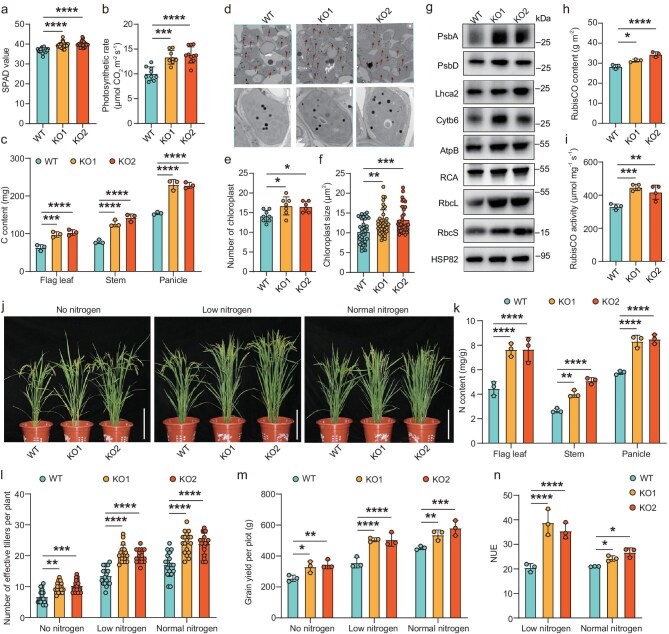
*OsNOP2* knockout promotes photosynthetic capacity and nitrogen use efficiency. (a–c) Statistical analysis of the SPAD value (a), photosynthetic rate (b) and the C content (c) of WT, KO1 and KO2 plants at the filling stage grown in the field under normal conditions. (d) TEM images of chloroplast ultrastructure from the flag leaves of WT, KO1 and KO2 plants. Scale bar: 5 μm. (e and f) Statistical analysis of the number (e) and size (f) of chloroplasts in mesophyll cells in WT, KO1 and KO2 plants. (g) Western blot analysis of the expression levels of proteins related to photosynthesis in WT, KO1 and KO2 plants. (h and i) RubisCO content (h) and RubisCO activity (i) in the fourth leaf of WT, KO1 and KO2 seedlings. (j) Single-plant phenotypes of WT, KO1 and KO2 under various nitrogen fertilization conditions [no nitrogen (0 kg ha^−1^), low nitrogen (112.5 kg ha^−1^) and normal nitrogen (225 kg ha^−1^)]. Scale bars: 25 cm. (k) The N content of WT, KO1 and KO2 plants at the anthesis stage grown in the field under normal conditions. (l–n) Statistical analysis of the number of effective tillers per plant (l), grain yield per plot (m) and NUE (n) of WT, KO1 and KO2 plants under various nitrogen fertilization conditions. Data are mean ± SD. (a and b): *n* ≥ 8 plants; (c), (k), (m) and (n): *n* = 3 biological replicates; (e and f): *n* ≥ 6 biological replicates; (h and i): *n* ≥ 3 biological replicates; (l): *n* ≥ 15 plants). *P < 0.05, **P < 0.01, ***P < 0.001, ****P < 0.0001. In (a and b), (e and f), and (h and i), *P* values are from one-way ANOVA (and non-parametric or mixed). In (c), (k), (m) and (n), *P* values are from two-way ANOVA (and non-parametric or mixed).

To further investigate the physiological processes through which *OsNOP2* knockout leads to the observed enhancement of development and yield in rice, a variety of physiological measurements were performed with *OsNOP2*-KO and WT plants. We determined pigment content and found that *OsNOP2*-KO leaves had significantly higher levels than WT (Fig. S17a). Consistently, soil–plant analysis and development (SPAD) assays also had significantly higher (7.2%–8.3%) SPAD values in *OsNOP2*-KO lines than WT at the filling stage (Fig. 2a). The photosynthesis rate of *OsNOP2*-KO plants is higher (32.8%–37.5%) than that of WT at the filling stage under field conditions (Fig. 2b). The stomatal conductance and transpiration rates showed that both indexes were significantly higher in *OsNOP2*-KO plants relative to WT (Fig. S17b and c). Consistently, swarm photosynthesis was also significantly enhanced (∼6.5%) in *OsNOP2*-KO plants compared to WT (Fig. S17d). Analysis of carbon contents indicated that *OsNOP2*-KO lines had significantly higher levels in flag leaves, panicles and stems compared to WT, with panicles showing the highest carbon allocation (Fig. 2c). These results suggested the accumulation of photosynthetic products in these organs, particularly panicles.

To investigate cellular level morphological changes that potentially contribute to the observed physiological traits, we conducted transmission electron microscopy (TEM) analysis of chloroplast ultrastructure. We found that the thylakoid membrane of *OsNOP2*-KO chloroplasts was significantly tighter than WT (Fig. S17e) at the seedling stage, and the chloroplast numbers (14.4%–15.8%) and size (15.0%–25.2%) of *OsNOP2*-KO mesophyll cells were also significantly increased compared to WT plants at the filling stage (Fig. 2d–f). Further western blot detection of several photosynthesis-related proteins showed obvious increases in light harvesting complex (LHC) proteins, PSII, cytochrome b6/f and ATP synthases in the fourth leaves of 3-week-old seedlings in *OsNOP2*-KO compared to WT (Fig. 2g). Detection of the photosynthesis marker, ribulose-1,5-bisphosphate carboxylase oxygenase (RuBisCO) [25], showed that its content increased by 10.6%–21.5% under *OsNOP2* knockout (Fig. 2h). Similarly, the RuBisCO activity also significantly increased by 26.5%–35.2% in *OsNOP2*-KO samples compared to that in WT (Fig. 2i). Taken together, these results implied that increased photosynthetic capacity could at least partially explain the observed increases in yield of *OsNOP2*-KO plants.

Due to the *OsNOP2*-KO lines having a larger root system than WT, we next evaluated the impacts of *OsNOP2* knockout on sensitivity to nitrogen in 2-week-old seedlings. Phenotypes and statistical analysis of shoot or root length showed less sensitivity in response to changing NO_3_^−^ concentrations in the *OsNOP2*-KO lines compared with WT seedlings (Fig. S18a–c). Moreover, we also treated seedlings with ^15^N-KNO_3_, and the results showed that the ^15^N content in the shoot and root of *OsNOP2*-KO seedlings was significantly higher than that of WT, and the nitrogen uptake activity and the nitrogen transport activity from root to shoot was higher in *OsNOP2*-KO seedlings compared with that in WT (Fig. S18d–g). To further verify the regulatory effect of *OsNOP2*-KO plants on nitrogen uptake and transport, we conducted experiments under various nitrogen fertilizer conditions in the field (Fig. 2j). The nitrogen content analysis showed that the *OsNOP2*-KO lines had significantly higher levels in the flag leaves, panicles and stems, and more nitrogen was transferred to the leaves and panicles, which was beneficial for improving photosynthetic rate and increasing yield (Fig. 2k). *OsNOP2* knockout plants also exhibited markedly elevated carbon and nitrogen contents across diverse tissues compared to WT controls (Fig. S19). In addition, mature *OsNOP2*-KO plants had significantly more tillers, higher grain yield and higher nitrogen use efficiency (NUE) [26] compared to WT under both low and appropriate nitrogen supply conditions (Fig. 2l–n), suggesting that *OsNOP2* knockout leads to enhanced nitrogen uptake and utilization. Overall, these results supported that lacking functional *OsNOP2* increases the photosynthesis rate and nitrogen utilization efficiency, both of which contribute to the increased yield observed in plants.

#### OsNOP2 is an mRNA m^5^C demethylase in rice

**Figure 3.**
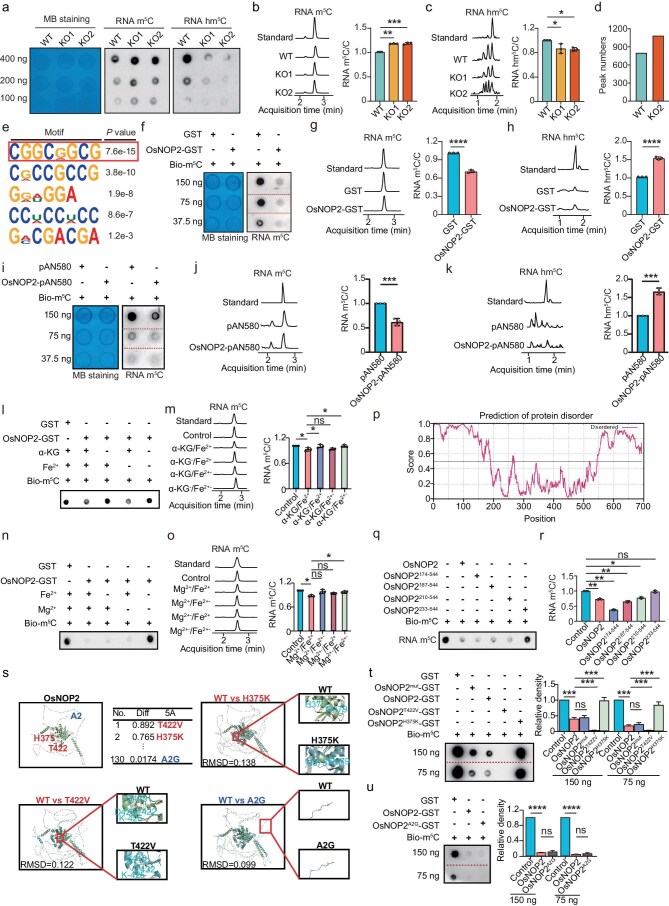
OsNOP2 is an mRNA m^5^C demethylase in rice. (a–c) Detection of total RNA m^5^C and RNA hm^5^C level from 3-week-old WT, KO1 and KO2 seedlings grown hydroponically by dot blot (a) and LC-MS/MS (b and c). MB, methylene blue staining. (d) The RNA m^5^C peak numbers within coding gene bodies in WT and KO2 seedlings. (e) Consensus sequence motifs of RNA m^5^C peaks. *P* value generated by HOMER. (f–h) Detection of demethylation of RNA m^5^C and RNA hm^5^C by purified OsNOP2-GST protein *in vitro*. Dot-blot assay (f) and LC-MS/MS quantification (g and h) confirmed demethylase activity. (i–k) Detection of the levels of RNA m^5^C and RNA hm^5^C after OsNOP2-pAN580 and pAN580 proteins were transiently expressed in rice protoplasts. Dot-blot assay (i) and LC-MS/MS quantification (j and k) confirmed demethylase activity. (l and m) *In vitro* demethylation of m^5^C by OsNOP2-GST in the presence of α-KG and Fe^2+^. Dot blot (l) and LC-MS/MS (m) show α-KG/Fe^2+^ dependence. (n and o) Replacement of Fe^2+^ with Mg^2+^ abolished OsNOP2 demethylase activity, as shown by dot blot (n) and LC-MS/MS (o). (p) Prediction of intrinsic disorder regions in the OsNOP2 protein sequence. (q and r) Detection of RNA m^5^C levels of OsNOP2 different truncated forms *in vitro* demethylation by dot blot (q) and LC-MS/MS (r). (s) Structural modeling and identification of candidate active sites. Molecular dynamics simulations pinpointed T422 and H375 as important residues. Superposition analysis showed conformational disruptions upon mutation. (t and u) Detection of the demethylase activity of the mutant proteins OsNOP2^T422V^ and OsNOP2^H375K^ (t) and OsNOP2^A2G^ (u) *in vitro*; the quantification is shown on the right. Data are shown as mean ± SD. (a–c), (f–o), (q), (r), (t) and (u): *n* = 3 biological replicates. ns, no significance, *P < 0.05, **P < 0.01, ***P < 0.001, ****P < 0.0001. In (b), (c), (m), (o), (r), (t) and (u), *P* values are from one-way ANOVA (and non-parametric or mixed). In (g), (h), (j) and (k), *P* values are from two-tailed Student’s *t*-tests.

To investigate the function(s) of OsNOP2 in cells, we first conducted subcellular localization assays using an OsNOP2-GFP fusion reporter co-expressed with H_2_B-mCherry in rice protoplasts, which revealed that OsNOP2-GFP was localized in the nucleus (Fig. S20). OsNOP2 is a member of the NOL1/NOP2/NSUN RNA methyltransferase family, in which the HsNSUN2 in humans and OsNSUN2 in rice have been identified as mRNA m^5^C methyltransferases through sequencing analysis [16,19,27]. Next, we examined potential changes in RNA m^5^C levels in WT and *OsNOP2*-KO seedlings. Unexpectedly, RNA m^5^C levels were significantly higher in *OsNOP2*-KO seedlings than in WT (Fig. 3a and b). Liquid chromatography tandem mass spectrometry (LC-MS/MS) detection of m^5^C and 5-hydroxymethylcytosine (hm^5^C) modifications from WT, KO1 and KO2 seedlings showed that m^5^C levels were significantly higher in the mutant lines, and hm^5^C levels were significantly lower (Fig. S21), thus confirming that OsNOP2 functions as an mRNA m^5^C demethylase in rice. Consistent with this finding, further examination of RNA m^5^C levels in flag leaf, root and panicle tissue of mature plants also showed that RNA m^5^C levels were significantly higher in *OsNOP2*-KO plants compared to those in WT plants (Fig. S22). These trends together supported the possibility that OsNOP2 may function as an RNA m^5^C demethylase. The RNA m^5^C levels of *OsNOP2*-OE lines led to decreased RNA m^5^C levels compared with WT, and the RNA hm^5^C levels were also significantly increased, collectively supporting an RNA demethylase function of OsNOP2 (Fig. S23).

Research has identified TET2 as an Fe^2+^ and α-ketoglutarate (α-KG)-dependent RNA m^5^C dioxygenase that oxidizes m^5^C to generate hm^5^C, contributing to dynamic regulation of RNA modifications [14]. To assess whether OsNOP2 similarly regulates hm^5^C formation, we examined hm^5^C levels in *OsNOP2*-KO seedlings. Both dot-blot and LC-MS/MS analyses revealed significantly reduced RNA hm^5^C levels in *OsNOP2*-KO lines (Fig. 3a and c). We therefore conducted RNA m^5^C methylated immunoprecipitation and sequencing (MeRIP-seq) of the WT and *OsNOP2*-KO2 transcriptomes in 3-week-old seedlings, which respectively identified 801 and 1087 (*P* < 0.05) putative m^5^C sites (Fig. 3d, Table S16). We then employed MEME Suite [28] to analyze motifs specifically bound by OsNOP2 in the MeRIP-seq data (Fig. 3e), and the top-ranked motif was chemically synthesized as a substrate in *in vitro* activity assays. Enzymatic activity assays *in vitro* with purified recombinant OsNOP2-GST and in rice protoplasts demonstrated that OsNOP2 could indeed mediate RNA m^5^C demethylase activity (Fig. 3f–k).

We conducted comparisons between human NOP2 and OsNOP2 that revealed obvious evolutionary divergence and structural differences (Figs S24 and S25a–c). The amino acid sequence similarity between OsNOP2 and human NOP2 is approximately 38% (Fig. S24). Superposition of predicted 3D structures indicated an RMSD value of 1.592, indicating that there were structural differences between the two proteins. *In vitro* enzyme activity assays showed that purified human NOP2 does not exert demethylase activity and that OsNOP2 does not exert methyltransferase activity (Fig. S25d and e). Previous studies have examined two cysteines in human NOP2 that function in product release and catalysis [28], and we found that mutating these two cysteines in OsNOP2 to alanine (OsNOP2^mut^) did not affect binding affinity for m^5^C-modified RNA substrates (Fig. S25f). Therefore, the similarity between the OsNOP2 and human NOP2 has obvious difference, further suggesting that the two have distinct functional roles.

Further *in vitro* assays revealed that demethylation activity was abolished in the absence of α-KG, whereas Fe^2+^ was dispensable (Fig. 3l and m). To test the hypothesis that Mg^2+^ compensates for Fe^2+^ in OsNOP2 function, we assessed the effect of Mg^2+^ deficiency. Mg^2+^ deficiency alone had little impact, but the combined deficiency of Fe^2+^ and Mg^2+^ nearly abolished demethylation activity, suggesting a synergistic requirement for divalent cations (Fig. 3n and o). Together, these results demonstrate that OsNOP2 acts as an mRNA m^5^C demethylase in rice.

#### T422 and H375 of OsNOP2 are key regulatory residues for demethylase activity

To identify the functional domain of OsNOP2 responsible for RNA m^5^C recognition, we performed bioinformatic analysis to predict the intrinsically disordered regions within the protein. The results indicated that the region spanning amino acids 174–544 is structured rather than disordered (Fig. 3p). To further define the minimal region required for m^5^C binding, we generated a series of OsNOP2 truncation mutants and performed dot-blot assays using a biotin-labeled m^5^C RNA probe. The results showed that deletion of amino acids 174–209 did not affect demethylase activity, whereas truncation beyond residue 232 led to a near-complete loss of enzymatic function, suggesting that this internal region is critical for OsNOP2 activity (Fig. 3q and r). Repeating these *in vitro* enzymatic assays and mass spectrometry analyses with proteins expressed and purified in eukaryotic (*Spodoptera frugiperda*) cells yielded results consistent with our previous findings obtained through a bacterial expression system (Fig. S26).

As TET family proteins are DNA 5mC demethylases, and TET2 is also responsible for RNA m^5^C demethylation in mammals [14,15], we conducted dot-blot assays and mass spectrometry analyses of DNA 5mC levels in KO1, KO2 and WT rice seedlings *in vivo*, and evaluated OsNOP2-GST demethylation activity towards DNA Bio-5mC and RNA Bio-m^5^C *in vitro*. No difference in DNA 5mC levels was detected between mutant and WT plants, nor was any demethylation observed in Bio-5mC substrates *in vitro* (Fig. S27). These results demonstrated that OsNOP2 does not exhibit DNA 5mC demethylase activity. In addition, high-performance liquid chromatography (HPLC) detection of all nucleosides (m^5^C, hm^5^C, A, U, C and G) with each of the truncation variants confirmed that no contamination with alternative nucleases contributed to the observed demethylation activity (Fig. S28).

To investigate the sites contributing to the demethylase activity of OsNOP2, we assessed conservation of residues at each site using position-specific amino acid probability (PSAP) analysis [29]. Through analysis, we obtained the diff values for the conversion of a specific amino acid in the protein to another amino acid. Higher diff values generally indicate that the mutation has caused significant structural changes in the protein, which may affect its stability or function. In contrast, lower diff values suggest that the mutation has a smaller impact, leading to less pronounced changes in the protein’s function or structure [30]. Based on diff values, we then selected three amino acid substitutions with the highest and lowest values for designing mutant proteins (Tables S17 and S18). Comparison of the predicted 3D protein structures before and after the amino acid substitutions with AlphaFold 3 showed that a threonine residue at position 422 (T422) was likely to form two hydrogen bonds with glycine at position 419 (G419) and additional hydrogen bonds with a lysine at position 425 (K425) and an aspartic acid at position 426 (D426). Substituting T422 with valine resulted in an RMSD value of 0.122 between OsNOP2 and OsNOP2^T422V^, with the hydrogen bond at G419 reduced to a single bond, and the hydrogen bond with D426 lost altogether in the variant. Upon substituting an isoleucine residue 375 (H375) with K, no substantial changes in hydrogen bonding were observed, and the RMSD value between WT and OsNOP2^H375K^ was 0.138 (Fig. 3s). *In vitro* enzymatic activity assays showed that the OsNOP2^T422V^ mutant exhibited an obvious increase in demethylase activity (∼48%), whereas the OsNOP2^H375K^ mutant almost completely lost demethylase activity compared with WT (Fig. 3t). Additionally, mutant proteins such as A2G exhibited no substantial changes in RNA m^5^C demethylation activity from WT (Fig. 3u).

To further validate the role of these amino acid sites in OsNOP2 protein function in the plant, we employed base editors (BEs) to introduce the T422V and H375V residue conversions in OsNOP2 protein. After obtaining homozygous lines for each point mutant, phenotypic analysis indicated that OsNOP2^T422V^ mutant seedlings had significantly lower than WT seedlings, while the OsNOP2^H375K^ mutant had higher plant height (Fig. S29). The changes in RNA m^5^C modification levels were consistent with the results of our detection using the *in vitro* assays mentioned above (Fig. 3t). These results further supported OsNOP2 functioning as an RNA m^5^C demethylase in rice, with T422 and H375 sites playing a key role in its function.

#### OsNOP2 knockout enhances translation efficiency of its target genes in rice

**Figure 4.**
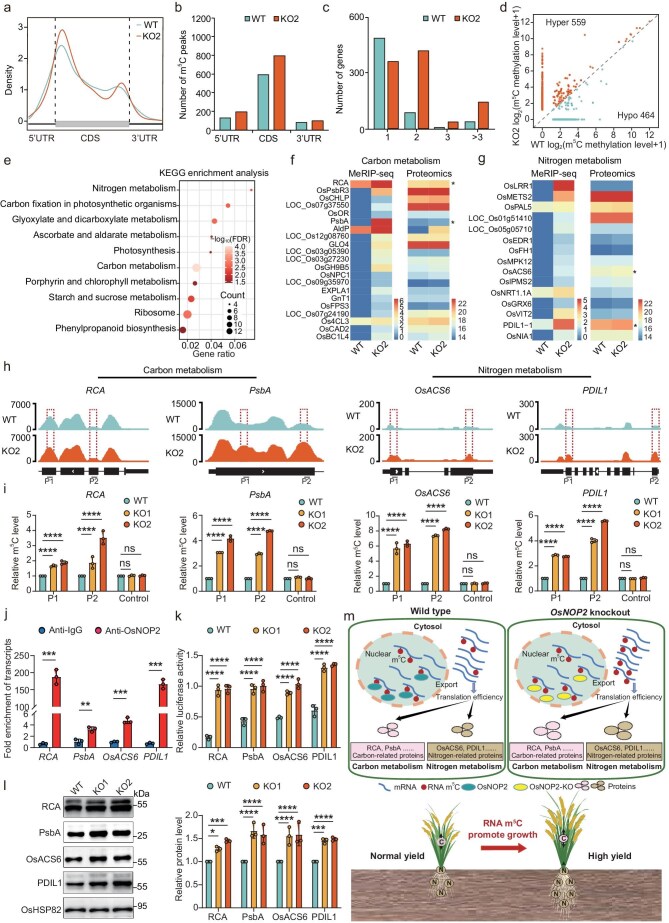
RNA m^5^C enhances translation efficiency of the target transcripts. (a and b) The distribution (a) and number (b) of RNA m^5^C peaks within coding gene bodies divided into 5′UTR, CDS and 3′UTR from 3-week-old WT and KO2 seedlings. (c) Statistics of the number of genes bearing 1, 2, 3 and >3 RNA m^5^C peaks. (d) Scatter plot of transcripts modified by RNA m^5^C in WT and KO2 seedlings. Hyper-m^5^C-modified transcripts are in red; hypo-m^5^C-modified transcripts are in blue. (e) KEGG analysis of hyper-m^5^C-modified transcripts. (f and g) Heatmap of transcripts related to carbon metabolism and nitrogen metabolism in WT and KO2 seedlings from MeRIP-seq and proteomics analysis. Stars denote the four m^5^C-regulated target genes (from MeRIP-seq) that were also detected in proteomics analysis. (h) Browser representation of the m^5^C reads of *RCA, PsbA, OsACS6* and *PDIL1* detected in MeRIP-seq displayed by integrative genomics viewer (IGV) tracks. The red box marked with P1 and P2 represents the experimental verification regions. (i) The relative RNA m^5^C level of *RCA, PsbA, OsACS6, PDIL1* and *Control* detected by MeRIP-qPCR. (j) RIP-qPCR showing OsNOP2 bound to *RCA, PsbA, OsACS6* and *PDIL1* by using anti-rabbit IgG and anti-OsNOP2 antibodies, respectively. (k) The relative luciferase activity of RCA, PsbA, OsACS6 and PDIL1. (l) Western blot analysis of the accumulation of RCA, PsbA, OsACS6 and PDIL1 of WT, KO1 and KO2 seedlings. The protein levels were normalized with HSP82 and ImageJ was used for quantification. (m) Schematic model summarizing the functions of OsNOP2 as an mRNA demethylase. Data are mean ± SD. (i–l): *n* = 3 biological replicates. ns, no significance, *P < 0.05, **P < 0.01, ***P < 0.001, ****P < 0.0001. *P* values are from two-way ANOVA (and non-parametric or mixed).

In light of our above finding of OsNOP2 RNA m^5^C demethylase activity, we next applied m^5^C-MeRIP-seq in WT and *OsNOP2*-KO2 3-week-old seedlings to explore target transcripts potentially mediated by OsNOP2. Comparison of m^5^C sites in the 5′UTR, CDS and 3′UTR regions of mRNAs between WT and *OsNOP2*-KO2 seedlings showed generally similar distributions of m^5^C sites between corresponding regions across genotypes, with a higher frequency in the CDS than UTR regions (Fig. 4a). However, the overall number of peaks was higher in each of these transcript regions in KO2 samples compared to WT (Fig. 4b, Table S16). Further analysis of m^5^C sites in the WT showed that 77.87% of the m^5^C-containing mRNAs had only one m^5^C site, 14.54% had two m^5^C sites, and 7.59% of such m^5^C-containing mRNAs had three or more m^5^C sites, whereas 37.68%, 43.63% and 18.68% of transcripts had one, two or three or more m^5^C sites, respectively, in the *OsNOP2*-KO2 transcriptome (Fig. 4c), suggesting that OsNOP2 might only demethylate specific target transcripts.

Assessment of global methylation across all m^5^C-modified mRNAs identified 1084 OsNOP2-dependent m^5^C sites spanning 559 hyper-m^5^C-modified transcripts in *OsNOP2*-KO2 plants compared to WT (Fig. 4d, Table S19) (*P* < 0.05). Subsequent Gene Ontology (GO) analysis of these hyper-m^5^C-modified transcripts showed enrichment for terms including ‘carbohydrate metabolic process’ and ‘organonitrogen compound biosynthetic process’ (Table S20). Further Kyoto Encyclopedia of Genes and Genomes (KEGG) analysis similarly suggested that the hyper-m^5^C-modified transcripts were enriched in pathways related to ‘carbon metabolism’, ‘photosynthesis’, ‘nitrogen metabolism’ and ‘ribosome’ (Fig. 4e, Table S21). Taken together, these findings indicated that the OsNOP2-dependent m^5^C modification of mRNA may play an important role in carbon metabolism and nitrogen metabolism.

Previous studies have reported that RNA m^5^C participates in regulating mRNA stability and mRNA translation [16,17,31]. We therefore conducted RNA-seq (Table S21) to identify genes with altered expression due to loss of OsNOP2 function. This analysis uncovered 636 differentially expressed genes (DEGs; |FC| ≥ 2, and *P* < 0.05) between WT and *OsNOP2*-KO2 transcriptomes. However, only six of these DEGs were modified by RNA m^5^C, and there was no significant correlation between RNA m^5^C level and abundance of these DEG transcripts (Fig. S30). To exclude the possibility that RNA m^5^C negatively affects the stability of transcripts, we conducted RNA decay assays for these six genes. These assays showed that all six genes had comparable mRNA decay rates between *OsNOP2* knockout and WT lines (Fig. S31). We subsequently carried out proteomics analysis to evaluate whether alterations in RNA m^5^C modification could potentially influence the translation level of some OsNOP2 target transcripts (Table S22). Correlation analysis between hyper-m^5^C-modified transcripts and their corresponding proteins showed that RNA m^5^C level was indeed positively correlated with protein abundance (R = 0.2; *P* = 0.00081) (Fig. S32). KEGG analysis showed that these hyper-m^5^C-modified transcripts were generally enriched in processes related to either ‘carbon metabolism’ or ‘nitrogen metabolism’. To confirm that blocking RNA m^5^C demethylation by OsNOP2 enhanced translation efficiency of its target genes, we also examined polysome loading of the target transcripts by Ribo-seq analysis. We found that global ribosome occupancy (Fig. S33a and b), as well as ribosome occupancy of OsNOP2-dependent target gene mRNAs, was significantly higher in KO2 lines than WT, as was translation efficiency (Fig. S33c–e). Examination of carbon metabolism and nitrogen metabolism (Fig. S33f and g) subsets, as well as individual genes in these respective subsets showed even more prominent increases in translation efficiency in KO2 plants compared to WT (Fig. S33h and i). Heatmap visualization showed that the majority of such ‘carbon metabolism’- or ‘nitrogen metabolism’-related proteins accumulated to higher levels in *OsNOP2*-KO2 plants compared to WT (Fig. 4f and g). These results suggested that increasing m^5^C modification level may promote translation efficiency of hypermethylated transcripts related to carbon metabolism and nitrogen metabolism.

Returning to our above MeRIP-seq data from WT and *OsNOP2*-KO rice, we identified that hyper-m^5^C-modified mRNAs related to photosynthesis included *RCA, PsbA, OsLIR1, OsCHLP, OsPORA* and *OsCAO1* [32–37], while nitrogen-related hypermethylated mRNAs were transcribed from *OsACS6, PDIL1, Fd-GOGAT1* and *OsNIA* [38–41]. RNA m^5^C immunoprecipitation qPCR (RIP-qPCR) assay for these analyte transcripts confirmed the trends observed in the m^5^C-MeRIP-Seq analysis (Fig. 4h and i, Fig. S34). We selected two genes related to ‘carbon metabolism’ (*RCA* and *PsbA*) and two genes related to ‘nitrogen metabolism’ (*OsACS6* and *PDIL1*) for further research. Further RIP-qPCR assays using an OsNOP2-specific polyclonal antibody in WT and *OsNOP2*-KO1/KO2 seedlings indicated that OsNOP2 could bind to each of these four transcripts (Fig. 4j). Further luciferase (LUC) trans-activation assays in which *LUC* was fused to gene fragments containing RNA m^5^C motifs driven by CaMV35S showed that expression of the functional RNA m^5^C fragments of each of these four transcripts resulted in significantly higher LUC activity in *OsNOP2*-KO protoplasts compared to their LUC signal in WT protoplasts (Fig. 4k). Immunoblotting also indicated that RCA, PsbA, OsACS6 and PDIL1 protein levels were significantly higher in *OsNOP2*-KO seedlings than WT (Fig. 4l). These results thus demonstrated that disrupting OsNOP2-dependent mRNA m^5^C demethylation could enhance translation efficiency of the transcripts related to carbon metabolism and nitrogen metabolism (Fig. 4m).

#### Knockout of *OsNOP2* promotes grain yield in LG31 and XS134 varieties

**Figure 5.**
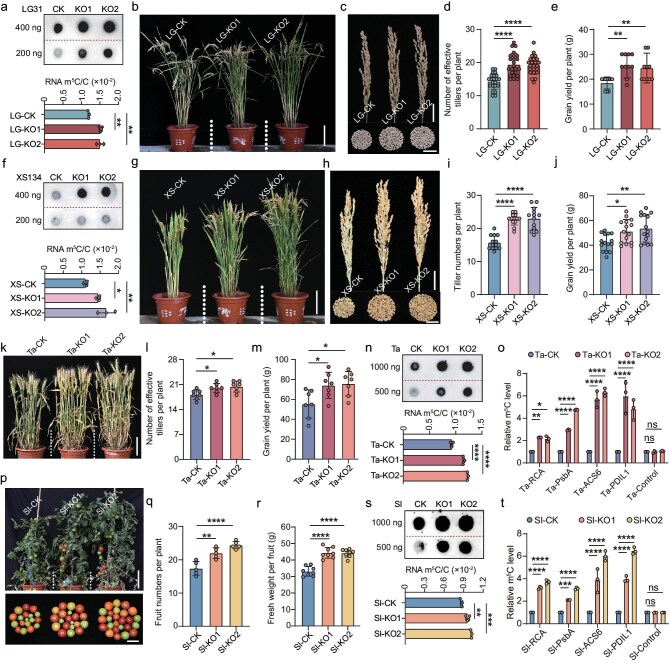
The demethylase and yield regulation of OsNOP2 orthologs are conserved in different rice varieties, wheat and tomato. (a and f) Detection of total RNA m^5^C level of flag leaves from rice of LG31 and XS134 backgrounds by dot blot (top) and LC-MS/MS (bottom). (b and c) Phenotype of single-plant (b) and panicle (c) from LG31 plants in Langfang in 2023. Scale bars: 20 cm (b) and 3 cm (top) and 1 cm (bottom) (c). (d and e) Statistical analysis of the number of effective tillers per plant (d) and grain yield per plant (e). (g and h) Phenotype of single-plant (g) and panicle (h) from XS134 plants in Langfang in 2023. Scale bars: 20 cm (g) and 3 cm (top) and 1 cm (bottom) (h). (i and j), Statistical analysis of the number of effective tillers per plant (i) and grain yield per plant (j). (k) Phenotype of single-plant from wheat. Scale bar: 20 cm. (l and m) Statistical analysis of the number of effective tillers per plant (l) and grain yield per plant (m). (n) Detection of total RNA m^5^C level of flag leaves from wheat by dot blot (top) and LC-MS/MS (bottom). (o) The relative RNA m^5^C level of *Ta*-*RCA, Ta*-*PsbA, Ta*-*OsACS6, Ta*-*PDIL1* and *Ta*-*Control* detected by MeRIP-qPCR. (p) Phenotype of single-plant (top) and total fruit from a single plant (bottom) from tomato. Scale bars: 20 cm (top) and 3 cm (bottom). (q and r) Statistical analysis of fruit numbers per plant (q) and fresh weight per fruit (r). (s) Detection of total RNA m^5^C level of flag leaves from tomato by dot blot (top) and LC-MS/MS (bottom). (t) The relative RNA m^5^C level of *Sl*-*RCA, Sl*-*PsbA, Sl*-*OsACS6, Sl*-*PDIL1* and *Sl*-*Control* detected by MeRIP-qPCR. Data are mean ± SD. (a), (f), (n), (o), (s) and (t): *n* = 3 biological replicates; (d) and (e): *n* ≥ 10 plants; (i) and (j): *n* ≥ 15 plants; (l) and (m): *n* = 7 plants; (q): *n* = 5 plants; (r): *n* = 8 fruits. ns, no significance, *P < 0.05, **P < 0.01, ***P < 0.001, ****P < 0.0001. In (a), (d–f), (i), (j), (l–n) and (q–s), *P* values are from one-way ANOVA (and non-parametric or mixed). In (o) and (t), *P* values are from two-way ANOVA (and non-parametric or mixed).

Next, we tested whether *OsNOP2* knockout could also increase RNA m^5^C levels and grain yields in other rice varieties. For this purpose, we induced *OsNOP2*-KO by CRISPR-Cas9 gene editing in two widely cultivated, high-yield varieties, LG31 (Fig. S35a) and XS134 (Fig. S36a). After generating homozygous lines for each genotype (*OsNOP2*-LGKO and *OsNOP2*-XSKO), dot-blot and LC-MS/MS analysis showed that both *OsNOP2*-LGKO and *OsNOP2*-XSKO had significantly increased RNA m^5^C levels (26%–27.2% and 30.7%–50.7%) compared to the corresponding LG31 and XS134 plants, respectively (Fig. 5a and f). *OsNOP2*-LGKO plants had a significantly higher SPAD value and photosynthesis rate than LG31 at the filling stage under field conditions (Fig. S35b and c). Phenotypic analysis of yield-related traits indicated that *OsNOP2*-LGKO plants had significantly increased the number of effective tillers per plant (34.2% to 35.6%), primary branch (16.9% to 30.7%), secondary branch (24.9% to 28.7%), grain numbers per panicle (15.9% to 18.5%), grain weight per panicle (35.6% to 45.6%) and 1000-grain weight (26.8% to 27.9%) compared to LG31, although there was no significant change in panicle length (Fig. 5b–d and Fig. S35d–i). Moreover, *OsNOP2*-LGKO plants had 32.4%–39.5% significantly higher grain yield per plant compared to LG31 (Fig. 5e).

Similarly, SPAD value and photosynthesis rate were also significantly higher in *OsNOP2*-XSKO plants compared to XS134 at the filling stage (Fig. S36b and c). Phenotypic analysis of the *OsNOP2*-XSKO lines showed that the number of effective tillers per plant (44.4%–45.9%), panicle length (6.6%–7.4%), secondary branch (62.3%–67.4%), grain numbers per panicle (19.9%–21.3%), seed setting rate (∼84.0%), grain weight per panicle (62.1%–73.0%) and 1000-grain weight (11.2%–19.9%) were all significantly increased compared to XS134 under field conditions in Langfang in 2023 (Fig. 5g–i, Fig. S36d–i). Grain yield per plant was also significantly greater in *OsNOP2*-XSKO plants than that in XS134 under field conditions in Hainan, in 2023 (21.2%–26.8%) (Fig. 5j). Notably, the *OsNOP2*-LGKO and *OsNOP2*-XSKO lines both exhibited significantly improved grain size, including grain length, grain width, grain area and grain perimeter (Fig. S35j and k, Fig. S36j and k). Thus, these results demonstrated that *OsNOP2* knockout could confer similar improvements in growth and yield across different elite rice lines under field conditions, supporting its potential for regulation in rice production.

#### Wheat and tomato NOP2 orthologs exhibit demethylase function and improve yield-related traits

To test whether OsNOP2 orthologs in other crops displayed functional conservation with its role in rice, we used gene editing to generate OsNOP2 ortholog knockout lines in the wheat variety Kenong199 (KN199), and the Ailsa Craig (AC) tomato background. To exclude functional redundancy from TaNOP2 homologs in the hexaploid wheat subgenomes, we induced double knockout of *TaNOP2*-aabb homologs. Genotyping by PCR and Sanger sequencing confirmed that we successfully obtained homozygous knockout lines in both wheat and tomato (Figs S27a and S38a).

The *TaNOP2*-KO plants had significantly increased the number of effective tillers per plant (∼25.9%), plant height (7.5%–9.8%), panicle length (10.0%–13.8%), grain numbers per panicle (34.6%–49.2%), grain weight per panicle (64.6%–82.0%) and 1000-grain weight (16.1%–18.4%) compared with KN199 (Fig. 5k and l, Fig. S37b–h). Ultimately, knockout of NOP2 orthologs in wheat resulted in boosting of the grain yield per plant by ∼33% compared to KN199 (Fig. 5m). In tomato, we observed significant increases in multiple agronomic traits of tomato, including fruit numbers per plant (from 26.4% to 40.2%), fresh weight per fruit (from 33.2% to 33.8%), plant height (from 18.6% to 19.5%) and fresh root weight per plant (from 61.4% to 79.0%) compared with AC (Fig. 5p–r, Fig. S38b–e). Additionally, we found that photosynthesis rate, stomatal conductance and transpiration rate were significantly improved in *TaNOP2*-KO and *SlNOP2*-KO plants compared with KN199 and AC, respectively (Figs S37j–l and S38f–h). Detection of RNA m^5^C modification levels by dot blot and LC-MS/MS showed that m^5^C levels were significantly increased in the *TaNOP2*-KO and *SlNOP2*-KO lines compared to that in KN199 and AC control plants, respectively (Fig. 5n and s). Subsequent immunoprecipitation of m^5^C-modified mRNAs using an anti-m^5^C antibody in wheat and tomato showed enrichment for m^5^C modifications on *RCA, PsbA, ACS6* and *PDIL1* transcripts in the OsNOP2 ortholog knockout lines, which were further validated by real-time qPCR (Fig. 5o and t). Collectively, these findings indicated that OsNOP2 orthologs also functioned as RNA m^5^C demethylases in wheat and tomato, and knockout of these NOP2 orthologs could improve multiple yield-related traits across monocot and dicot crop species.

## DISCUSSION

In this study, we found that OsNOP2 functions as a novel RNA m^5^C demethylase that participates in determining photosynthetic capacity and nitrogen use efficiency in rice by mediating mRNA m^5^C modifications (Fig. 4m). Loss of OsNOP2 function leads to enhanced grain yield in multi-plots, under high temperature conditions, and in saline soils compared with WT. More importantly, *OsNOP2* knockout in the elite rice varieties, LG31 and XS134, or knockout of its orthologs in wheat and tomato, could similarly enhance yield in these plants. Overall, our study demonstrates how modulation of an epigenetic regulatory gene can improve multiple yield-related traits and increase yield even under heat- and salt-stress conditions. Moreover, our findings suggest that mRNA demethylases may present an attractive target for genetic improvement that may show high functional conservation across crops.

RNA m^5^C modifications have been extensively studied in animals and are known to regulate mRNA stability, mRNA translation and interactions between proteins and RNA molecules [2,4]. NOP2 is known to function as an m^5^C methyltransferase, and impacts on cell proliferation and links to various cancers in human [12]. The OsNSUN2 regulates high temperature through mediating RNA m^5^C levels [16]. We have found that OsNOP2 is the first m^5^C demethylase that controls multiple rice yield traits in rice. Knockout of *OsNOP2* led to significantly increased translation efficiency of *RCA, PsbA, OsACS6* and *PDIL1* transcripts, which participate in carbon and nitrogen metabolism. Findings in our current study indicate that OsNOP2-mediated RNA m^5^C modification can regulate RNA translation efficiency, which affects photosynthetic capacity and nitrogen utilization. In addition, structural analysis together with site-directed mutagenesis showed that OsNOP2 residues T422 and H375 are required for its function. An expanded, landscape perspective of the mechanisms underlying the function of m^5^C modifications in regulating target mRNAs, and modulating the RNA m^5^C demethylase activity of OsNOP2 through targeted protein engineering may enable fine-tuning of RNA methylation levels to improve crop yields under various environmental conditions.

The trade-off between yield and stress tolerance is among the most crucial issues in crop breeding. Notably, previous studies of m^6^A regulation in crops—including heterologous expression of the human fat mass and obesity-associated protein FTO to decrease m^6^A levels—have reported substantial increases in grain yield in multiple crops, as well as effects on drought stress response in rice [42]. Epigenetic regulatory genes that are conserved across various crops have been proposed as a valuable resource for breeding high-yield crop lines with heat and salt tolerance [26]. In this study, we found that RNA m^5^C demethylase activity of OsNOP2 exerts broad regulatory effects on plant growth and yield-related traits that are conserved across monocot and dicot crops. Given the success of this strategy in rice, wheat and tomato, it is likely that NOP2 modulation may have potential for breeding strategies in other crops. Thus, our study highlights the benefits of increasing RNA m^5^C levels to increase yield and stress adaptation in crops. Designing *NOP2* knockouts or screening its natural non-functional alleles increase the RNA m^5^C level, which is a promising strategy to improve crop yield.

## Supplementary Material

nwag102_Supplemental_Files

## Data Availability

Raw sequence data generated during this study have been deposited in the National Center for Biotechnology Information (NCBI) BioProject database under accession number PRJNA1157433. The proteomics mass spectrometry and ribosome profiling datasets have been deposited in the ProteomeXchange Consortium via the iProX partner repository under accession numbers PXD057871 and PRJCA053645, respectively. All the other data are available from the corresponding authors upon request.

## MATERIALS AND METHODS

Detailed Materials and Methods are available in the Supplementary data.
