# Supplementary material for: Elevating RNA m5C methylation provides a promising strategy for crop productivity
Source: Natl Sci Rev. 2026 Feb 12;13(10):nwag102. doi: 10.1093/nsr/nwag102 (PMC13249431; doi:10.1093/nsr/nwag102)
Supplement: nwag102_Supplemental_Files [file nwag102_supplemental_files.zip › supplementary methods and figures.docx]

**Elevating** **RNA m^5^C methylation provides a promising strategy for crop productivity**

Xiulan Li^1†^, Cong Li^1†^, Xiangyu Wang^1†^, Liwen Yang^1†^, Weijun Guo^1^, Yichao Mao^1^, Hanlin Liu^1^, Dongwei Li^1^, Shuangyong Yan^2^, Yong Zhang^3^, Xiaofeng Gu^1*^, Li Pu^1*^

^1^Biotechnology Research Institute, Chinese Academy of Agricultural Sciences; Beijing 100081, China

^2^Tianjin Key Laboratory of Crop Genetics and Breeding, Tianjin Crop Research Institute, Tianjin Academy of Agricultural Sciences; Tianjin 300384, China

^3^Chongqing Key Laboratory of Plant Resource Conservation and Germplasm Innovation, Integrative Science Center of Germplasm Creation in Western China (Chongqing) Science City, School of Life Sciences, Southwest University, Chongqing 400715, China

∗Corresponding authors. E-mail: [guxiaofeng@caas.cn](mailto:guxiaofeng@caas.cn); [puli@caas.cn](mailto:puli@caas.cn)

^†^These authors contributed equally to this work

**Methods**

**Transgene constructs**

*OsNOP2* knock-out lines were generated using CRISPR-Cas9 in the *Nipponbare* (WT), Longgeng31 (LG31), and Xiushui134 (XS134) backgrounds, as previously described [1-3]. Transgenic plants were confirmed by sequencing PCR products amplified with primers (OsNOP2-KO-F/R). The full-length coding sequence (CDS) of *OsNOP2* was amplified from *Nipponbare* cDNA and cloned into the binary vector p23A-Act1 (digested with *Sma*I and *Xba*I) to create the overexpression construct pAct1::*OsNOP2*. This plasmid was introduced into *Nipponbare* via *Agrobacterium* strain EHA105 [4].

To generate complementation (COM) lines, the full *OsNOP2* sequence (including the promoter and CDS) was amplified from *Nipponbare* genomic DNA and cloned into p23A (digested with *Kpn*I), resulting in the pOsNOP2::*OsNOP2* vector. This construct was introduced into the *OsNOP2-KO2* background (Cas9-free).

CRISPR-Cas9 KO lines were also developed for wheat (*Triticum aestivum*, Kenong199) [5] and tomato (*Solanum lycopersicum*, Ailsa Craig) [6]. Transgenic plants were confirmed by sequencing PCR products amplified with primers (Ta-F/R and Sl-F/R). All primers used are listed in Table 8.

**Plant growth conditions**

Homozygous rice seeds used in hydroponic and field experiments were at least T3 generation. Hydroponic seedlings were grown in modified Kimura B solution (replenished every three days) under short-day conditions (8 hours light/16 hours dark) at 28°C with 300 µmol m⁻² s⁻¹ light intensity and 70% relative humidity in a greenhouse.

Field experiments were conducted in Langfang and Sanya from 2019 to 2023. Langfang (39°36′28″N, 116°36′53″E, Hebei Province) features a warm temperate monsoon climate, while Sanya (18°23′23″N, 109°11′48″E, Hainan Province) has a tropical oceanic monsoon climate. Growing seasons were May to October in Langfang and December to April in Sanya. Plot sizes and planting arrangements varied over the years (details in Tables S2-7). Border plants were excluded from analyses to minimize edge effects.

In 2023, wheat was grown in a greenhouse under short-day conditions (12 hours light/12 hours dark) at 22–24°C in Beijing. Field experiments were conducted from January to June 2024 at the Institute of Crop Science, CAAS, Beijing. Each line was planted in 1.5-m-long rows with 30 cm spacing between rows. Tomato plants were grown in a greenhouse under a 16 hours light/8 hours dark photoperiod at 24 ± 2°C with 70% relative humidity and 300 ± 100 µmol m⁻² s⁻¹ light intensity. Six plants per line were grown in pots spaced 30 cm apart.

Nitrogen use efficiency (NUE) was evaluated in rice under three nitrogen treatments (0, 112.5, 225 kg N ha⁻¹) at the Huzhou Experimental Field, Zhejiang University, from May to October 2023. Urea was applied at the tillering (40%) and heading (60%) stages. Plots were randomized with a spacing of 25 cm between rows and plants, with at least 60 plants per line.

**Abiotic stress phenotypic evaluation**

To evaluate the growth performance of WT, KO1, and KO2 plants under high-temperature stress during the reproductive phase, a plastic greenhouse was erected after the flowering stage and maintained until grain maturity. The study was conducted in Langfang from May to October 2022 and 2023. High-temperature stress was applied during two stages: tillering to jointing and booting to flowering. Each line was planted in plots of 6 rows × 10 plants with 20 cm row spacing and 15 cm plant spacing. At least three plots were used as biological replicates, with all lines completely randomized in each plot.

For salt stress evaluation, WT, KO1, and KO2 plants were cultivated in 3.8‰ salt concentration paddy fields in Dongying, Shandong Province, and their phenotypes and agronomic traits were analyzed. To further assess salt stress tolerance, WT and KO2 plants were grown in 2.5‰ salt concentration paddy fields in Wuqing District, Tianjin, and their yield traits were evaluated. Sodium chloride (NaCl) was supplied throughout the entire developmental cycle. Each line was planted in plots of 6 rows × 10 plants with 20 cm row spacing and 15 cm plant spacing in Dongying and in plots of 202 rows × 10 plants in Wuqing. At least three plots were cultivated as biological replicates, with all lines randomized in each plot.

**RNA Extraction and reverse transcription quantitative PCR (RT-qPCR)**For gene expression analysis, dot-blot, and LC-MS/MS assays, samples were collected from shoots and roots of 3-week-old hydroponically grown seedlings and from leaves, stems, roots, and panicles of varying lengths (5 cm, 10 cm, and 15 cm) during the booting stage from field-grown plants. All samples were immediately frozen in liquid nitrogen and stored at -80°C.

Total RNA was extracted using an RNAprep Pure Micro Kit (TIANGEN, Cat# DP420) following the manufacturer’s protocol. RNA quantification was performed using a NanoDrop 2000 spectrophotometer (Thermo Fisher Scientific, USA). For qRT-PCR analysis of *OsNOP2* expression in different tissues, 1 µg total RNA was reverse-transcribed using the HiScript III 1st Strand cDNA Synthesis Kit (Vazyme, Cat# R312-02). RT-qPCR was conducted with SYBR Green mix (Vazyme, Cat# Q712-03) on an Applied Biosystems 7500 Fast Real-Time PCR system. *Ubq* (*LOC_Os03g13170*) served as the internal reference gene for normalization. Primers used are detailed in Table S12.

**RNA *in situ* hybridization**

RNA *in situ* hybridization was performed following a standard protocol [7]. Briefly, leaf tissues were fixed with formaldehyde, paraffin-embedded, and sectioned into 8-μm-thick slices using a Lecia sliding microtome (RM2265). The sections were dewaxed, digested with proteinase K (Roche), and dehydrated using an ethanol gradient. RNA probes were applied for hybridization, followed by incubation with anti-digoxigenin-AP Fab fragments (Roche). Signal visualization was performed using NBT/BCIP stock solution (Roche).

**PI staining**

PI staining was carried out with modifications to a previously reported method [8]. Root tips of 4-day-old WT, KO1, and KO2 rice seedlings were stained with PI for 1 minute, rinsed with deionized water for 30 seconds, and imaged using a Zeiss LSM 900 laser scanning confocal microscope. Root apical meristem length and meristem cell length were determined from confocal microscopy images using ImageJ software.

**EdU staining**

EdU staining was performed using a Click-iT EdU Alexa Fluor 488 HCS assay kit (Invitrogen, Cat# C10350) following the manufacturer’s protocol with minor modifications. Four-day-old WT, KO1, and KO2 seedlings were incubated in 20 μM EdU solution for 2 hours, fixed with 3.7% formaldehyde solution in PBS (pH 7.2) containing 0.1% Triton X-100, and subjected to vacuum infiltration for 20 minutes. EdU detection cocktail was applied for 30 min. Images were captured using the GFP channel of a microscope, and EdU-stained cells in root meristems were quantified using ImageJ software.

**Grain filling rate**

Grain filling rate was measured following a previously described method [9] with minor modifications. At the early heading stage, florets were marked on the surface of glumes with a black pen for two consecutive days. Fresh grains were collected at 7-day intervals post-flowering (Days 7, 14, 21, 28, and 35), dried at 105℃ to constant weight, hulled manually, and weighed. For each line, 100 grains were used as replicates, and three replicates were performed. Grain filling rate (GFR) at each stage was calculated as: *GFR_i_* = (*GW_i_ - GW_i-1_*)/7, where *GFR_i_* is the grain filling rate (mg grain^-1^ d^-1^), and *GW_i_* is the weight of dried grains (mg grain^-1^) at stage i (i = 1, 2, 3, 4, 5).

**Carbon and nitrogen content determination**

Rice plants grown in the field were sampled at the filling stage. Various organs, including flag leaves, stems, and panicles, were harvested and heated in an oven at 105°C for 30 minutes. The samples were then dried at 70°C for 3 days. The carbon and nitrogen contents were measured using thoroughly homogenized samples with the Elementar Vario EL Cube analyzer (Elementar, Germany).

**RubisCO content and activity measurements**

RubisCO content and activity were measured as previously described [10], with minor modifications. Briefly, the fourth leaf of 3-week-old rice seedlings was used for determination. To measure RubisCO content, 15 μg of soluble protein extracted from approximately 5 cm^2^ of leaf area was loaded onto 16% SDS-PAGE gels and separated by electrophoresis at 80 V for 3 hours. The gels were stained for 4 hours with 0.25% (w/v) Coomassie Brilliant Blue R-250 dissolved in 45% (v/v) methanol and 10% (v/v) acetic acid, followed by destaining using a solution of 20% methanol and 7% acetic acid. The large (55 kDa) and small (15 kDa) subunits of RubisCO were excised, incubated in 1 mL formamide at 50°C with shaking (180 rpm) overnight, and the absorbance of the eluate at 595 nm was measured. RubisCO content was quantified using a bovine serum albumin (BSA) calibration curve.

RubisCO’s initial activity was determined using a Plant Ribulose Diphosphate Carboxylase (RubisCO) kit (# G0602F24). Samples (~50 mg) were ground in 1 mL pre-cooled extraction buffer (50 mM Tris-HCl, pH 8.0, 0.5 mM EDTA, 10 mM MgCl_2_, 5 mM dithiothreitol, and 1% (w/v) PVPP) and incubated at 4℃ for 10 minutes. After centrifugation at 12,000 rpm at 4°C for 2 minutes, 50 µL of supernatant was mixed with reaction buffer (50 mM Tris-HCl, pH 8.0, 10 mM MgCl_2_, 1 mM EDTA, 20 mM NaHCO_3_, 5 mM dithiothreitol, 0.2 mM NADH, 5 mM ATP, 10 U Ml^-1^ creatine phosphokinase, 10 U mL^-1^ 3-phosphoglycerate phosphokinase, 10 U mL^-1^ glyceraldehyde-3-phosphate dehydrogenase, and 5 mM phosphocreatine). Absorbance at 340 nm was monitored within 60 seconds after adding 20 μL of 10 mM RuBP. Total RubisCO activity was measured by activating the enzyme for 10 minutes in reaction buffer without RuBP at 25℃, followed by adding 20 μL of 10 mM RuBP.

**Photosynthetic parameters measurement**

Photosynthetic parameters, including photosynthetic rate, stomatal conductance, and transpiration rate, were measured using the LI-6800 system (Biosciences, USA). Measurements were performed on field-grown rice and wheat plants at the heading stage and greenhouse-grown tomato plants at the flowering stage. Conditions were consistent across field and greenhouse experiments, except for light intensity. The measurement conditions included 400 μmol mol^-1^ CO_2_ concentration, 28 ± 1°C leaf temperature, 55–75% relative humidity, and a flow velocity of 500 μmol s^-1^. Each line had at least three replicates.

**SPAD value acquisition**

Chlorophyll content was measured using the SPAD Chlorophyll Analyzer (SPAD-502, Konica Minolta). After unrolling the main stem leaves of WT, KO1, and KO2 rice plants, 3–5 plants were randomly selected from each plot. SPAD values of the flag leaves were measured three times during the booting stage. The average SPAD value of flag leaves per plot was calculated and analyzed.

**Pigments measurement**

To measure pigments, 0.1 g of fresh rice leaves (without midveins) was cut into pieces, homogenized, and soaked in 95% ethanol to a final volume of 5 mL. Samples were kept at room temperature in darkness for 48 hours. The optical density of the extract was measured at 663 nm, 645 nm, and 470 nm using a spectrophotometer, and the contents of chlorophyll a, chlorophyll b, and carotenoids were calculated per unit mass.

**Transmission electron microscopy**

Rice leaves were cut into 0.1 mm × 0.2 mm sections and fixed in 2.5% (v/v) glutaraldehyde solution. Samples were vacuumed at 0.8–1.0 MPa until the leaves settled at the bottom of 2 mL tubes. Ultrathin sections (50–70 nm) were prepared and observed using a transmission electron microscope (Hitachi H-7650 TEM). Chloroplast size and number were quantified using ImageJ software.

**^15^N-nitrate uptake activity and root-to-shoot transport assays.**

^15^N-nitrate uptake and transport assays were performed as previously described [11]. Two-week-old WT, KO1, and KO2 seedlings grown under low nitrogen (200 μM) and high nitrogen (5 mM) conditions were nitrogen-starved for 3 days prior to ^15^N treatment. Seedlings were rinsed in 0.1 mM CaSO_4_ for 1 minute and transferred to nutrient solutions containing 200 μM or 5 mM ^15^N-KNO_3_ (Sigma-Aldrich) for 3 hours. Roots and shoots were separated, dried at 70°C for 3 days, and weighed. Nitrogen uptake activity was calculated as the amount of ^15^N absorbed by roots per unit dry weight per unit time, and the root-to-shoot transport ratio was determined by the ratio of ^15^N accumulation in shoots to that in roots.

**NUE determination**

Approximately 15 plants per line were harvested at maturity. Individual plants were threshed and dried, and NUE was calculated as the ratio of total grain yield to nitrogen applied, as previously described [12] .

**Subcellular Localization of OsNOP2**

To determine the subcellular localization of *OsNOP2*, the full-length cDNA of *OsNOP2* was amplified and inserted into the pAN580 vector digested with *Xba*I and *Sma*I, generating the 35S:*OsNOP2*:*GFP* fusion vector. Both the fusion vector and the empty vector were transiently co-transformed into rice protoplasts alongside pMcherry (35S:*OsMADS3*:*RFP*, as a marker) [13] at a 1:1 ratio. Rice protoplast extraction and PEG transformation were performed following established protocols with minor modifications [14]. Transfected protoplasts were incubated overnight at 28°C and visualized using confocal laser scanning microscopy (LSM 900). Primers used for constructing the fusion vector are listed in Table S9.

**Dot Blot Analysis of m^5^C and hm^5^C Levels**

Dot blot analysis was carried out as described previously with minor modifications. Purified RNA was denatured at 95℃ for 3 minutes, immediately chilled on ice, and serially diluted. RNA samples were spotted onto a Hybond-N^+^ membrane (Amersham), followed by UV crosslinking. After blocking the membrane with 5% non-fat milk in PBST, it was incubated with an anti-m^5^C antibody (Diagenode, MAb-081-010, 1:1,000) for 2 hours at room temperature. The secondary antibody, horseradish peroxidase-conjugated anti-mouse IgG (Santa Cruz), was applied for 1 hour. Signal detection was performed using an ECL Western Blotting Detection Kit (Thermo). The visualized membrane was staining with Methylene Blue.

**Detection of the m^5^C/C and hm^5^C/C Ratio by LC–MS/MS Analysis**

LC–MS/MS analysis was conducted according to established protocols [15]. Briefly, 1 µg of purified RNA was enzymatically digested into single ribonucleosides, followed by purification using a spin filter. Samples were analyzed using an Agilent 6490 triple quadrupole mass spectrometer.

**Vector Construction and Recombinant Protein Expression in *Escherichia coli***

The full-length sequence and site-directed mutagenesis variants of *OsNOP2* were cloned into the pGEX-4T-1 vector using *Bam*HI*/Eco*RI restriction sites, resulting in GST-tagged constructs. The recombinant plasmids were transformed into *Escherichia coli* Transetta (DE3) cells. Protein expression was induced at an OD_600_ of 0.6–0.8 using 0.2 mM IPTG at 4°C overnight. Purification was performed with Glutathione-Sepharose Resin according to the manufacturer’s instructions. Purified proteins (OsNOP2-GST, OsNOP2^mut^-GST, OsNOP2^T422V^-GST, OsNOP2^H375K^-GST, OsNOP2 ^A2G^-GST, OsNSUN2-GST, NOP2-GST, and Empty-GST) were analyzed via SDS-PAGE (6% gel). Primer sequences used in this experiment are provided in Table S9.

**RNA m^5^C demethylation assay**

The RNA m^5^C demethylation assay followed an adapted protocol [16]. A synthetic RNA probe (5′-GGCGAUGCGGm^5^CGGCGGAGGCCG-3′) was incubated in a reaction mixture containing 1 mM HEPES (pH 7.0), 1 mM KCl, 10 mM MgCl_2_, 100 mM Ascorbic acid, 100 mM α-Ketoglutaric acid, 100 mM (NH_4_)_2_ Fe (SO_4_)_2_·6H_2_O, 1 μg RNA probe, and 500 ng purified protein. Reactions were incubated at 23°C for 30 min. Empty-GST protein was used as a negative control. Reaction products were validated by dot blot analysis using an RNA m^5^C antibody (Diagenode, MAb-081-010, 1:1,000). Experiments were repeated three times. The RNA probe sequence is listed in Table S10.

**RNA m^5^C methylation assay**

The RNA m^5^C methylation assay utilized an MTase-Glo™ Methyltransferase Assay kit (Promega, Cat# V7601) with modifications. A 5′-GGCGAUGCGGCGGCGGAGGCCG-3′ RNA probe was synthesized *in vitro* (Integrated DNA Technologies). The 20 μL reaction mixture included 5 μL 4 × reaction buffer (1 M Tris-HCl pH 8.0, 5 M NaCl, 0.5 mM EDTA, 1 M MgCl_2_, 10% BSA, 1 M DTT, and 40 U mL^-1^ RNase inhibitor), 0.4 uL SAM, 1 μg RNA probe, and 500 ng purified protein. The solution was incubated at 23°C for 1 hour. Empty-GST protein served as a negative control. Products were confirmed by dot blot analysis using an RNA m^5^C antibody (Diagenode, MAb-081-010, 1:1,000). Experiments were conducted in triplicate. RNA probe details are provided in Table S10.

**RNA EMSA**

RNA EMSA was performed using a LightShift® Chemiluminescent RNA EMSA Kit (20158X, Thermo) and carried out as described previously [17] with some modifications. In brief, 1 µL synthetic RNA with m^5^C (100 nM final concentration) and 2 µg purified protein (Empty-GST, OsNOP2-GST, and OsNOP2^mut^-GST) were mixed and incubated in binding buffer (50 mM Tris-HCl pH 7.5, 100 mM NaCl, 0.4 mM EDTA, 0.1% NP-40, and 40 U mL^-1^ RNasin, 1 mM DTT, 50% glycerol, 5 ng μL^-1^ BSA) for 30 minutes at 22℃. Then, 1 μL glutaraldehyde (0.2% final concentration) was added into the RNA-protein complex and incubated for 15 minutes at 22℃. The RNA-protein mixture was mixed with 5 µL 5 × Hi-Density TBE Sample buffer (2 mL 5 × TBE buffer, 1.5 g Ficoll ^®^ 400, 1 mL 1% Bromophenol blue, 1 mL 1% Xylene Cyanole, RNase-free water to 10 mL) and separated from 6% TBE gel on ice for 2 hours at 80 V. The gel was transferred to a positively charged nylon membrane. Detection was performed per a LightShift® Kit instructions. RNA probe sequences are provided in Table S10.

**Construction of stable transgenic lines with site-directed mutagenesis of amino acids**

The construction of point mutant protein-stable lines was performed as described previously [17]. The primers used to identify the stable transgenic lines with site-directed mutagenesis of amino acids are listed in Table S11.

**m^5^C-meRIP-Seq and m^5^C-meRIP-qPCR**

m^5^C-meRIP-seq was carried out as described previously [18] with minor modifications. In brief, 300 μg of total RNA was fragmented into ~100-nt pieces using freshly prepared fragmentation buffer (800 μL RNase-free water, 100 μL 1 M Tris-HCl, pH 7.0, and 100 μL 1 M ZnCl₂, pH 7.0). The fragmented RNA was precipitated with reaction buffer (1/10 volume of 3 M NaAc, pH 5.2; glycogen at 100 µg·mL⁻¹ final concentration; and 2.5 volumes of 100% ethanol) and incubated at −80℃ overnight. A 20-μL aliquot of the fragmented RNA was reserved as an input control.

For immunoprecipitation, the reaction mixture consisted of 385 μL fragmented RNA, 5 μL RNase inhibitors (Promega), 5 μL ribonucleoside vanadyl complexes (Sigma-Aldrich), 100 μL 5 × IP buffer (0.5 mL Tris-HCl, pH 7.4; 1.5 mL 5 M NaCl; 0.5 mL 10% Igepal CA-630; and RNase-free water to 10 mL), and 5 μL m^5^C-specific antibody (Diagenode, Cat# C15200081, 1.24 μg·μL⁻¹). The mixture was incubated at 4 ℃ for 2 hours. Protein G beads (Thermo Fisher Scientific) were pre-blocked with 1 × IP buffer containing 1% BSA (0.5 mg·mL⁻¹) for 2 hours at room temperature and then incubated with the reaction mixture at 4℃ for an additional 2 hours to allow binding.

After washing twice, the RNA bound to the beads was eluted twice with 100 μL of freshly prepared 1 × elution buffer (90 μL 5 × IP buffer, 150 μL 20 mM 5-methylcytosine hydrochloride (Sigma-Aldrich), 7 μL RNase inhibitors, and 203 μL RNase-free water to 450 μL). The eluates and input control were pooled, precipitated with 2.5 volumes of 100% ethanol, and incubated at −80℃ overnight for subsequent experiments.

For m^5^C-meRIP-Seq, libraries were prepared using a NEBNext Ultra Directional RNA Library Prep Kit for Illumina (NEB) and sequenced on the Illumina HiSeq 2500 platform. To validate the results from m^5^C-meRIP-Seq, m^5^C-meRIP-qPCR was performed using the IP and input RNAs. These RNAs were reverse-transcribed with SuperScript™ III Reverse Transcriptase (Invitrogen™), and RNA m^5^C enrichment was quantified by normalizing to input. The primers used for verification are listed in Table S12.

**Peak Calling and Analysis**

Raw sequencing data were cleaned by removing low-quality reads, adapters, and rRNA sequences. The processed reads were aligned to the *Oryza sativa* reference genome (*Nipponbare*-BRI) [19] using Bowtie2 with default parameters (https://bowtie-bio.sourceforge.net/bowtie2/index.shtml). Only uniquely mapped reads were retained for peak detection. Peaks were identified using MACS2 [20] with an effective genome size of 380, 699, 722, comparing IP sample reads against input sample reads (*P* ≤ 0.05). Peaks were categorized into 5′UTR, CDS, and 3′UTR regions, and their sequences were extracted for m^5^C motif identification using MEME-ChIP [21].

**RNA-sequencing analysis**

Total RNA was extracted from the leaves of three-week-old rice seedlings grown in hydroponic culture and from panicles at different developmental stages (5 cm, 10 cm, and 15 cm). Libraries were prepared using an NEBNext Ultra Directional RNA Library Prep Kit for Illumina (NEB) following the manufacturer’s protocol. Libraries were sequenced on the Illumina HiSeq 4000 platform.

Adapter-containing reads, poly-N sequences, and low-quality reads were filtered out, and the resulting clean reads were mapped to the *Oryza sativa* genome (*Nipponbare*-BRI) [22] using Bowtie2 (<https://bowtie-bio.sourceforge.net/bowtie2/index.shtml>). Mapped reads were assembled using Cufflinks v2.1.1 [23], and gene counts were obtained with HTSeq v0.6.1 [24]. Gene expression levels were calculated as FPKM values using Cuffdiff. Differentially expressed genes were identified based on a threshold of |FC| ≥ 2 and *P* < 0.05.

**Ribosome sequencing**

Ribosome sequencing (Ribo-seq) was conducted using a GenSeq® Ribo Profile Kit (GenSeq, Inc.) following the manufacturer’s protocols. Briefly, cycloheximide-treated cells were lysed using lysis buffer and subjected to nuclease digestion. Ribosome-protected mRNA fragments were subsequently isolated via size exclusion chromatography. The RNA fragments were size-selected by polyacrylamide gel electrophoresis (PAGE) and processed for ribosomal RNA (rRNA) depletion. The purified RNA fragments then underwent end repair, ligation with 3’ adapters, and reverse transcription to generate cDNAs. The resulting cDNA libraries were purified by PAGE, circularized, and amplified via PCR. The final libraries were purified and sequenced on a NovaSeq platform (Illumina). After removing adapter sequences and low-quality reads from raw sequencing reads with cutadapt (v1.9.3), the clean reads were then aligned to the *Oryza sativa* genome (*Nipponbare*-BRI) reference genome using TopHat2. Gene-level read counts were obtained using HTSeq (v0.9.1), and data normalization was performed with edgeR.

**Proteomics Design and Analysis**

Proteins were extracted from the shoots of three-week-old WT and KO2 seedlings as described previously [24]. Briefly, samples were ground in liquid nitrogen and precipitated with TCA/acetone buffer, followed by resuspension in SDT buffer (4% SDS, 100 mM Tris-HCl, pH 7.6). The lysates were sonicated, boiled for 15 minutes at 95℃, and centrifuged at 12,000 rpm for 40 minutes. Protein concentrations were determined using a BCA assay.

For SDS-PAGE, 15 μg protein per sample was mixed with 5 × loading buffer, boiled at 95℃ for 5 minutes, and separated on a 4%-20% SDS-PAGE gel (180 V, 45 minutes). Protein bands were visualized using Coomassie Brilliant Blue R-250.

For DIA experiments, peptide samples were desalted using C18 cartridges and dissolved in 0.1% formic acid. Peptide concentrations were quantified by measuring UV absorbance at 280 nm. DIA experiments were conducted on a Vanquish Neo liquid chromatography system coupled to an Orbitrap™ Astral™ mass spectrometer (Thermo Scientific).

Peptide identification and quantification were performed using DIA-NN 1.8.1, with data filtered by a false discovery rate (FDR) of ≤ 1%. Protein identification was reported at a 99% confidence level.

***OsNOP2* RNA Immunoprecipitation (RIP) assay**

RNA Immunoprecipitation (RIP) was conducted following previously described protocols [25] with minor modifications. Three-week-old *Oryza sativa* (WT) seedlings were ground in liquid nitrogen, divided into two portions, and resuspended in 5 mL·g⁻^1^ lysis buffer (50 mM Tris-HCl, pH 7.4; 100 mM KCl; 2.5 mM MgCl_2_; 0.1% NP-40; 400 U·mL⁻^1^ RNase inhibitor; and 2 × protease inhibitor cocktail, Roche). The samples were incubated at 4℃ for 30 minutes with gentle rotation, followed by centrifugation at 12,000 rpm for 20 minutes.

The clarified lysates were pre-cleared for 20 minutes at 4℃ with 30 µg·mL⁻¹ Protein A (Roche). Subsequently, 50 µL of lysate was set aside as the input control, while the remaining lysate was incubated with either 4 µg·mL^-1^ anti-OsNOP2 (Beijing Enris Biotechnology Co., Ltd) or anti-IgG (Agrisera, Cat# AS10916) for 1 hour at 4℃ with rotation. Protein A (150 µg·mL⁻^1^) was added to the mixture and incubated for 3 hours at 4℃ with rotation. The beads were washed three times with lysis buffer, and the nucleic acid was recovered by treating the samples with three volumes of proteinase K solution (100 mM Tris-HCl, pH 7.4; 10 mM EDTA; 150 mM NaCl; 2% SDS; 0.2 µg·µL^-1^ proteinase K, Roche) at 65℃ for 15 minutes. RNA was extracted using an RNeasy Plant Mini Kit (QIAGEN, Cat# 154041662) according to the manufacturer’s protocol.

The extracted RNA was subjected to qPCR analysis. The ΔCt value between the 1% input and the immunoprecipitated RNA was calculated, and relative enrichment was normalized to input RNA. The primers used for verification are listed in Table S13.

**Luciferase Activity Analysis System in rice protoplasts**

The luciferase (LUC) activity assay was employed to investigate whether RNA m^5^C modification affects protein synthesis. The cDNA fragments of *RCA*, *PsbA*, *OsACS6*, and *PDIL1* were amplified from WT plants and fused into the PGreen II-0800-LUC vector as reporters. These constructs were transformed into WT, KO1, and KO2 rice protoplasts, respectively. Protoplast transformation was conducted following the subcellular localization protocol, and a Luciferase® Reporter Assay System (Promega) was used to measure LUC activity.

The ratio of LUC (firefly luciferase) to Ren (Renilla luciferase) was calculated to represent relative activity [26]. LUC signals were detected using a Tanon 5200 chemiluminescence imager. Each experiment was performed in triplicate. The primers used for verification are provided in Table S14.

**RNA stability**

To assess RNA stability, seven-day-old *OsNOP2*-KO1, -KO2, and wild-type *Nipponbare* (WT) seedling grown in hydroponic medium were exposed to 10 μM actinomycin D or 10 μM dimethyl sulfoxide (DMSO) vehicle. Shoots were harvested at 0, 8, and 24 h of treatment. Total RNAs were then from each sample, and relative expression was calculated by normalizing gene expression in actinomycin D-treated samples against that in DMSO-treated vehicle controls. Primers used to assess RNA stability are listed in Table S15.

**Western Blot Assay**

Western Blot Assay was performed as previously reported [10] with slight modifications. Total protein was extracted from the newly unfolded leaves of three-week-old rice seedlings. For proteomic verification, the samples were consistent with the sequencing data. Proteins were extracted in 500 µL of extraction buffer (50 mM Tris-HCl, pH 7.4; 150 mM NaCl; 1 mM DTT; 1 mM PMSF; 2 mM EDTA; 0.1% Triton X-100; and 1 × protease inhibitor cocktail, Roche).

A total of 60 µg of protein per sample was separated by SDS-PAGE and transferred to PVDF membranes (Amersham, GE Healthcare). Membranes were blocked with 5% skim milk in TBST buffer (8 g NaCl; 0.2 g KCl; 50 mL 1 M Tris-HCl, pH 7.4; 500 µL Tween; deionized water to 1 L) for 2 hours at room temperature.

The membranes were incubated with primary antibodies, including anti-Lhca2 (Agrisera, Cat# AS01006), anti-Lhcb2 (Agrisera, Cat# AS01003), anti-RbcL (Agrisera, Cat# AS03037), anti-RbcS (Agrisera, Cat# AS07259), anti-Cytb6 (Agrisera, Cat# AS06119), anti-PsbD (Agrisera, Cat# AS06146), anti-RCA (Agrisera, Cat# AS132634), anti-PsbA (Agrisera, Cat# AS05084), anti-OsACS6 (BGI, Cat# AbP80728-A-SE), and anti-PDIL1 (BGI, Cat# AbP80053-A-SE), at the recommended dilutions for 2 hours at room temperature.

Following this, the membranes were incubated with the goat anti-rabbit secondary antibody (Transgen, Cat# HS101-01) and the goat anti-mouse secondary antibody (Transgen, Cat# HS201-01) diluted 1:5,000 for 1 hour at room temperature. Anti-HSP82 (BGI, Cat# AbM51099-31-PU) was used as a loading control. Protein bands were visualized using the ECL Western Blotting Detection Kit (Thermo Fisher).


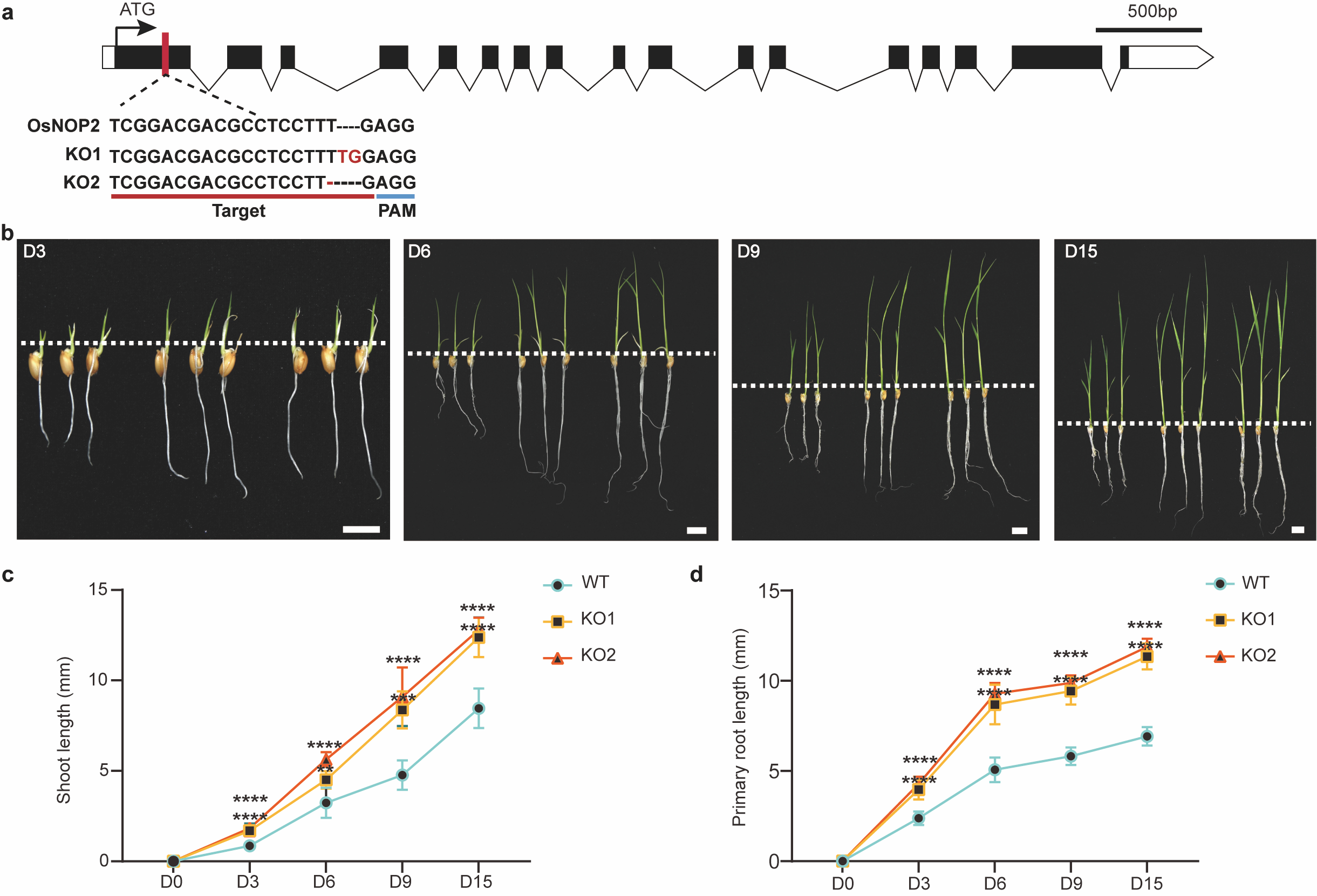


**Fig. S1 OsNOP2 is a negative regulator for** **seedling development. a**, The gene structure and mutation sites of KO1 and KO2. PAM, protospacer adjacent motif. **b**, Phenotype of seedlings at different developmental stages (Day 3, Day 6, Day 9, and Day 15). **c-d**, Statistical analysis of the shoot length (**c**) and primary root length (**d**) at different developmental stages in WT, KO1, and KO2 seedlings. Data are mean ± S.D. (n > 10 seedlings). ***P* < 0.01, ****P* < 0.001, *****P* < 0.0001; *P* values are from one-way ANOVA (and nonparametric or mixed).


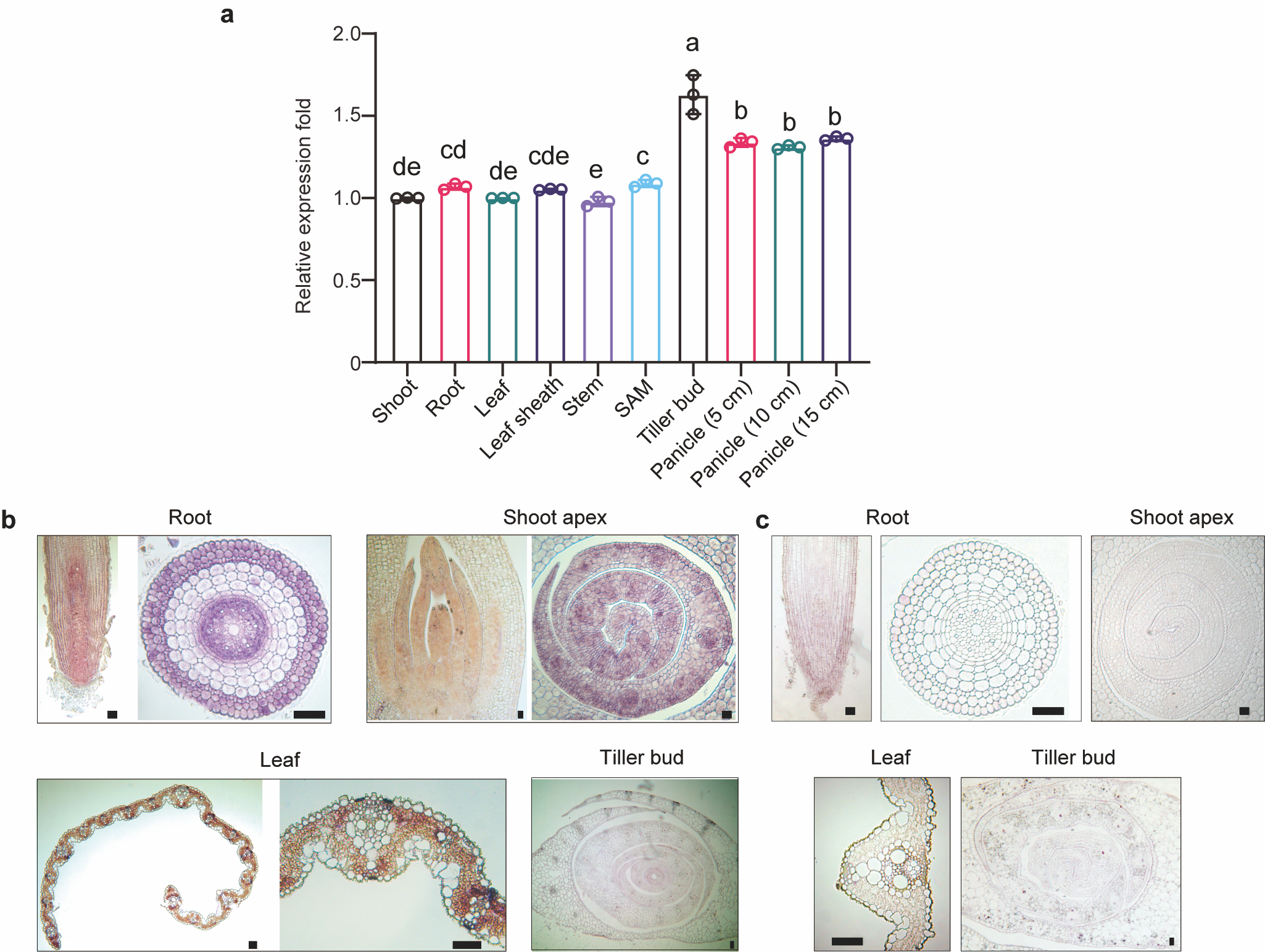


**Fig. S2 The expression patterns of *OsNOP2* in different tissues.** **a**, RT-qPCR shows the expression levels of the *OsNOP2* in different tissues (shoot, root, leaf, leaf sheath, stem, SAM, tiller bud, and panicles of different length). **b-c**, *OsNOP2* expression was validated by *in situ* hybridization assay in different tissues (root, shoot apex, leaf, and tiller bud) (**b**) and the sense probe as a negative control (**c**). Scale bar, 50 μm. Data are mean ± S.D. (**a**, n = 3 biological replicates). Letters indicate statistical significance among tissues by two-tailed Student’s t-tests.
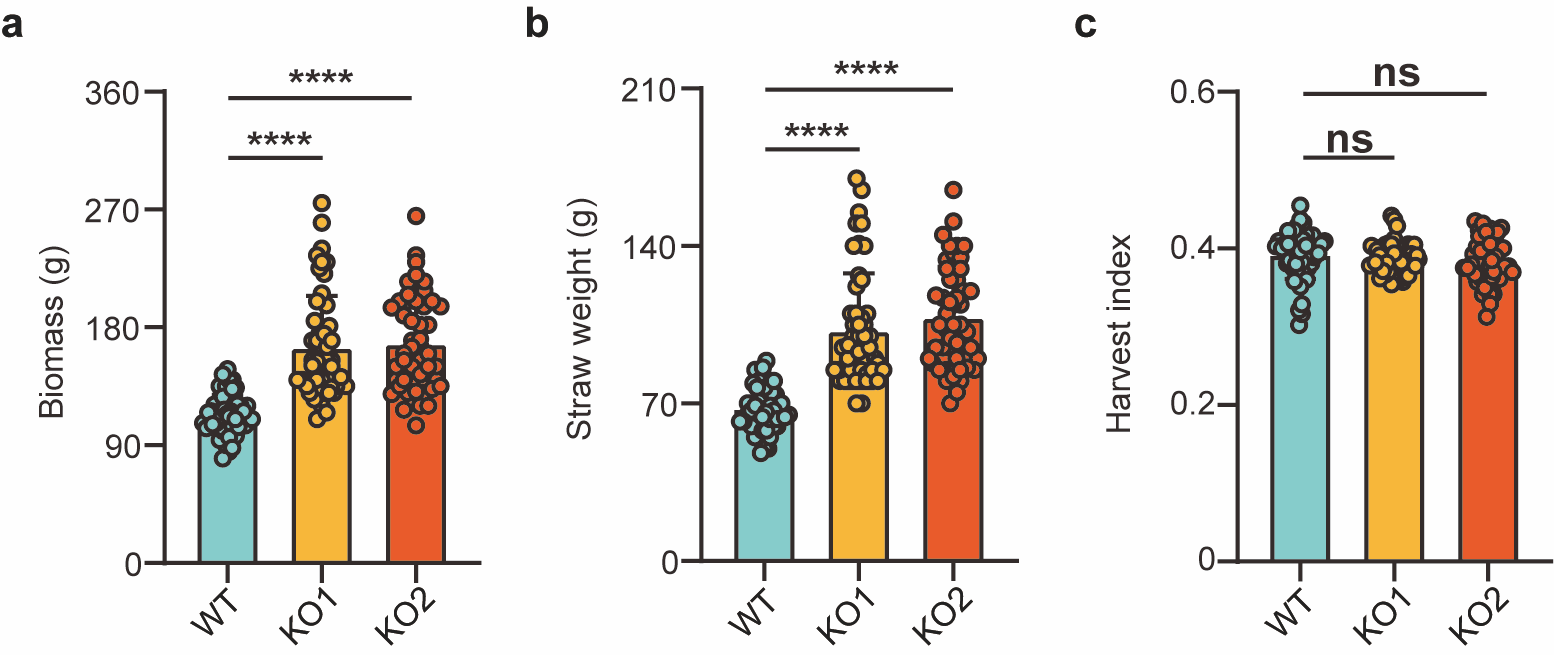


**Fig. S3** ***OsNOP2* knockout alters relevant parameters of rice biomass. a-c**, Statistical analysis of biomass (**a**), straw weight (**b**), and harvest index (**c**) from WT, KO1, and KO2 plants at the mature stage. Data are mean ± S.D. (n = 30 plants). ns, no significance, *****P* < 0.0001; *P* values are from one-way ANOVA (and nonparametric or mixed).


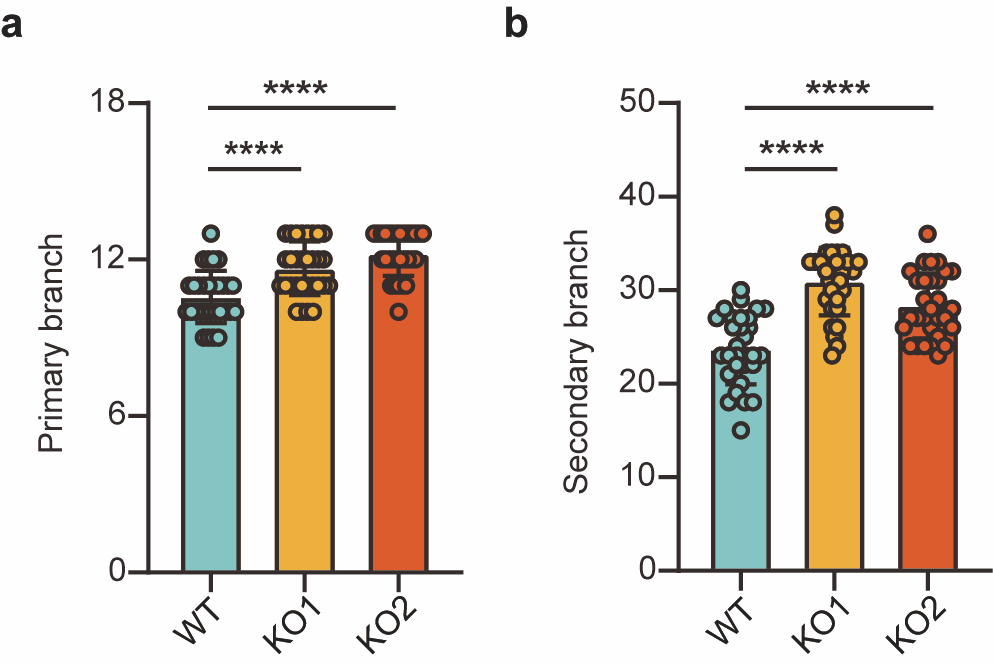


**Fig. S4 *OsNOP2* knockout increases relevant parameters of panicle traits. a-b**, Statistical analysis of primary branch (**a**) and secondary branch (**b**) from WT, KO1, and KO2 plants at the mature stage. Data are mean ± S.D. (**a-b**, n = 30 panicles). *****P* < 0.0001; *P* values are from one-way ANOVA (and nonparametric or mixed).


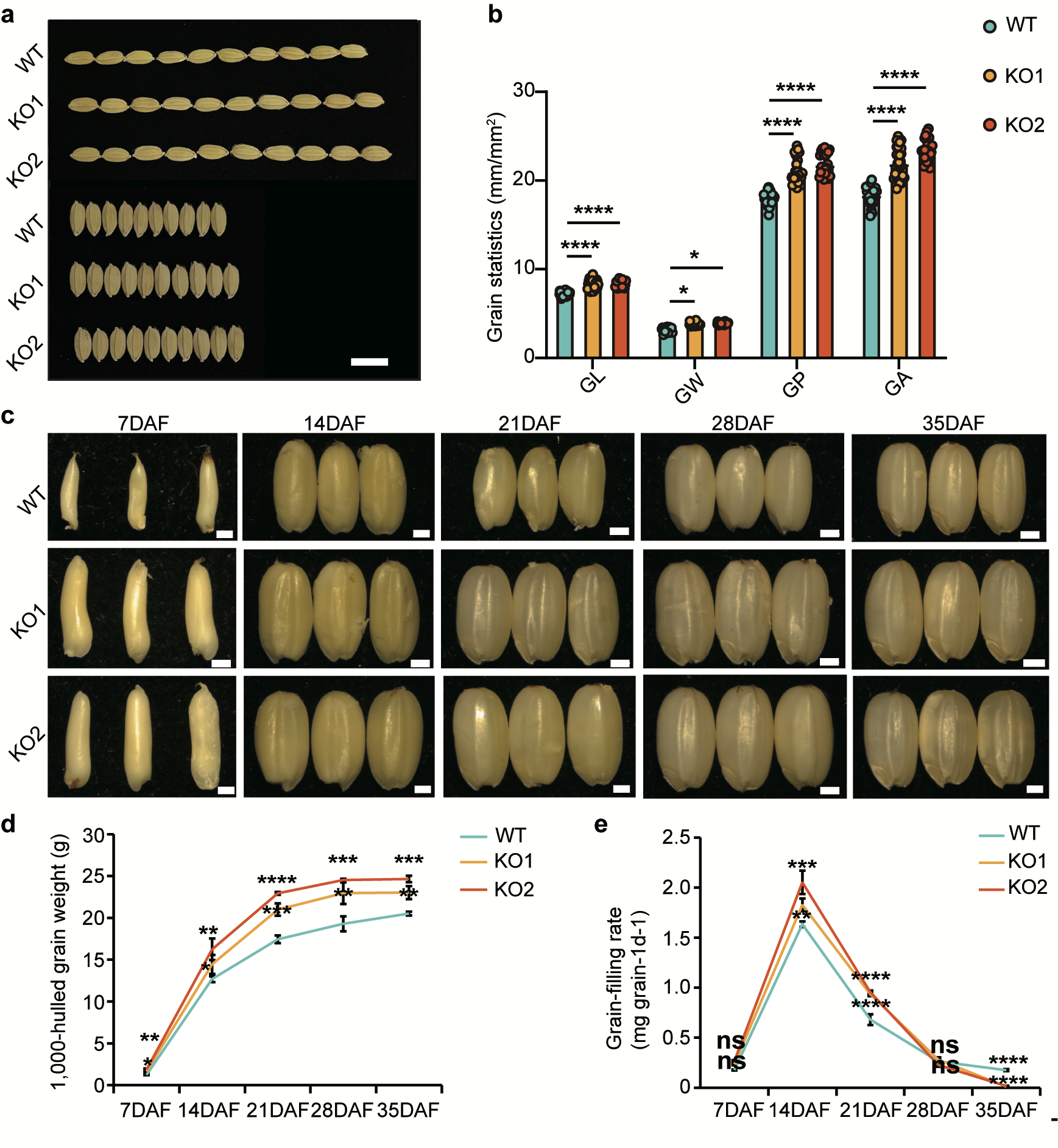


**Fig. S5 *OsNOP2* knockout promotes grain size and rice grain filling rate. a**, Grain morphologies of WT, KO1, and KO2 plants. Scale bar, 1 cm. **b**, Statistical analysis of grain length (GL), grain width (GW), grain perimeter (GP), and grain area size (GA) from WT, KO1, and KO2 plants. **c**, Morphological changes in brown rice grains of WT, KO1, and KO2 plants at five stages. Scale bar, 2 mm. **d**, Statistical analysis of brown rice weight and grain-filling rate changes of WT, KO1, and KO2 plants at five stages. Data are mean ± S.D. (**b**, n = 50 grains; **d-e**, n = 3 biological replicates). ns, no significance, **P* < 0.05, ***P* < 0.01, ****P* < 0.001, *****P* < 0.0001; *P* values are from one-way ANOVA (and nonparametric or mixed).


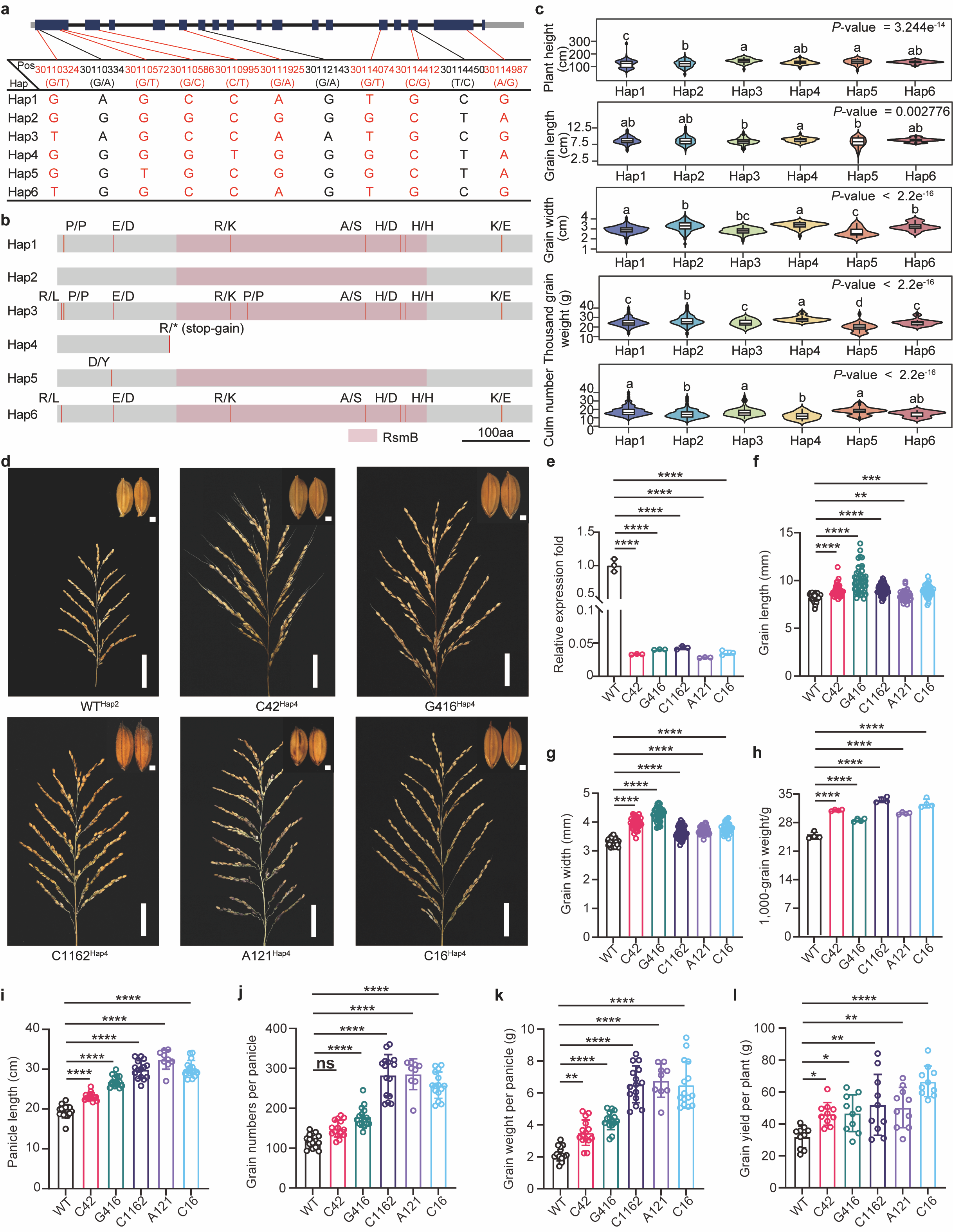


**Fig. S6 Genetic diversity of *OsNOP2* in the 3K RG dataset. a**, Haplotypes of *OsNOP2* in 2,753 accessions of 3K RG (rare haplotypes of < 100 accessions are not shown) using 11 SNPs in the CDS region. **b**, The protein structures of six haplotypes. The reference and the alternative amino acid are represented by the abbreviation before and after slash, respectively. Stop-gain indicates the early termination of translation. **c**, Performance distribution of different haplotypes of *OsNOP2* on plant height, grain length, grain width, thousand grain weight and culm number in 3K RG. Different letters on the boxplots indicate statistically significant differences (*P* < 0.01, Duncan’s new multiple range test). **d**, Panicle and grain morphology of some accessions from the Hap2 (WT) and Hap4 (C42, G416, C1162, A121, and C16). Scale bar, 3 cm. **e**, Relative expression levels of *OsNOP2* in WT^Hap2^, C42^Hap4^, G416^Hap4^, C1162^Hap4^, A121^Hap4^, and C16^Hap4^ plants. **f-l**, Statistical analysis of grain length (**f**), grain width (**g**), 1,000-grain weight (**h**), panicle length (**i**), grain numbers per panicle (**j**), grain weight per panicle (**k**), and grain yield per plant (**l**) of the accessions from the Hap2 and Hap4. Data are mean ± S.D. (**e**, n = 3 biological replicates; **f-g**, n ≥ 20 grains; **h**, n = 4 biological replicates; **i-k**, n = 15 panicles; **l**, n = 10 plants). ns, no significance, **P* < 0.05, ***P* < 0.01, ****P* < 0.001, *****P* < 0.0001; *P* values are from one-way ANOVA (and nonparametric or mixed).


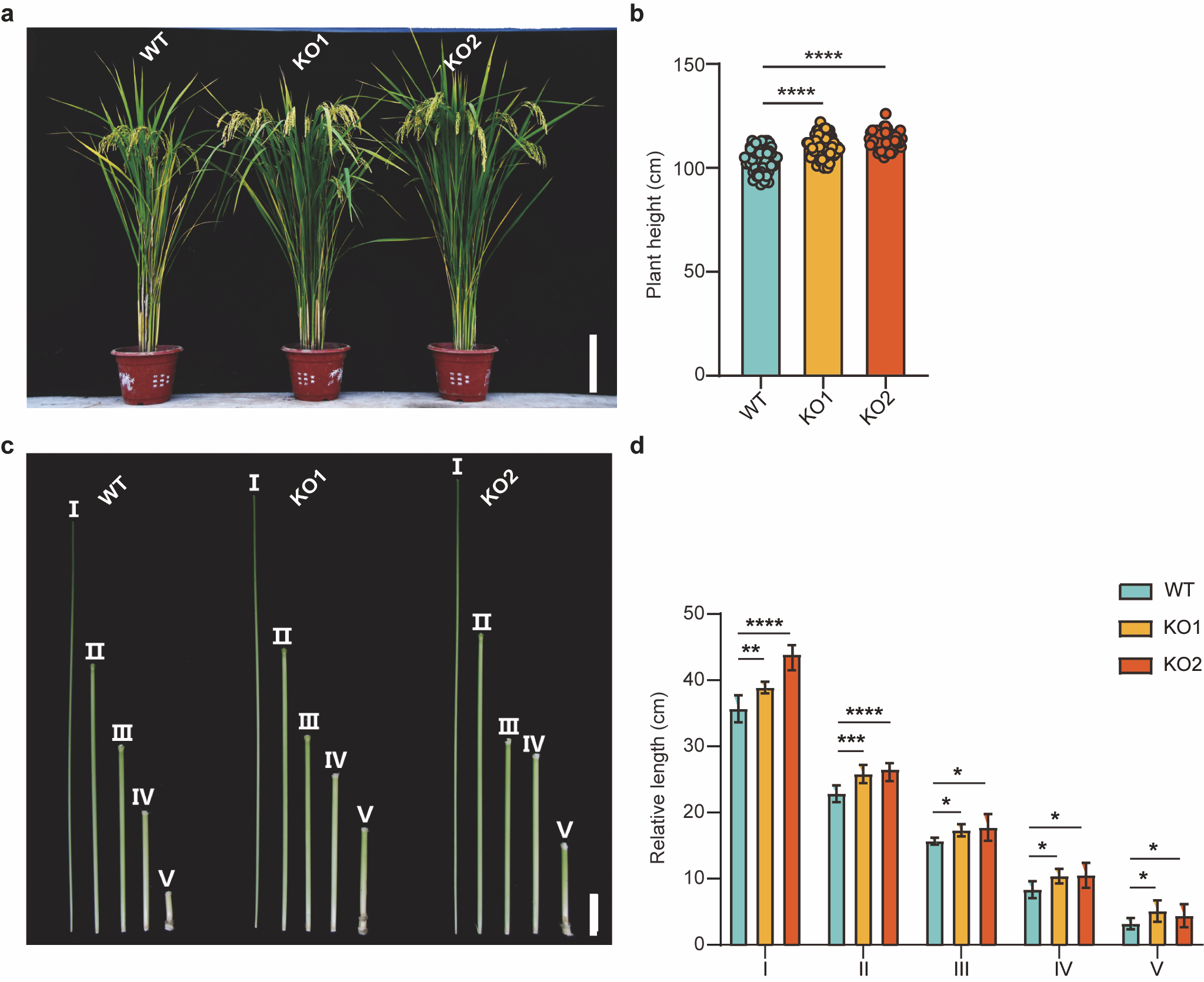


**Fig. S7 *OsNOP2* knockout alters rice morphology. a**, **c**, Phenotype of single-plant (**a**) and internode (**c**) of WT, KO1, and KO2 plants at the grain-filling stage. Scale bars, 15 cm (**a**) and 3 cm (**c**). **b**, **d**, Statistical analysis of the plant height (**b**) and relative length of internodes (**d**) of WT, KO1, and KO2 plants at the mature stage. Data are mean ± S.D. (**b**, n = 30 plants; **d**, n = 10 plants). **P* < 0.05, ***P* < 0.01, ****P* < 0.001, *****P* < 0.0001; *P* values are from one-way ANOVA (and nonparametric or mixed).


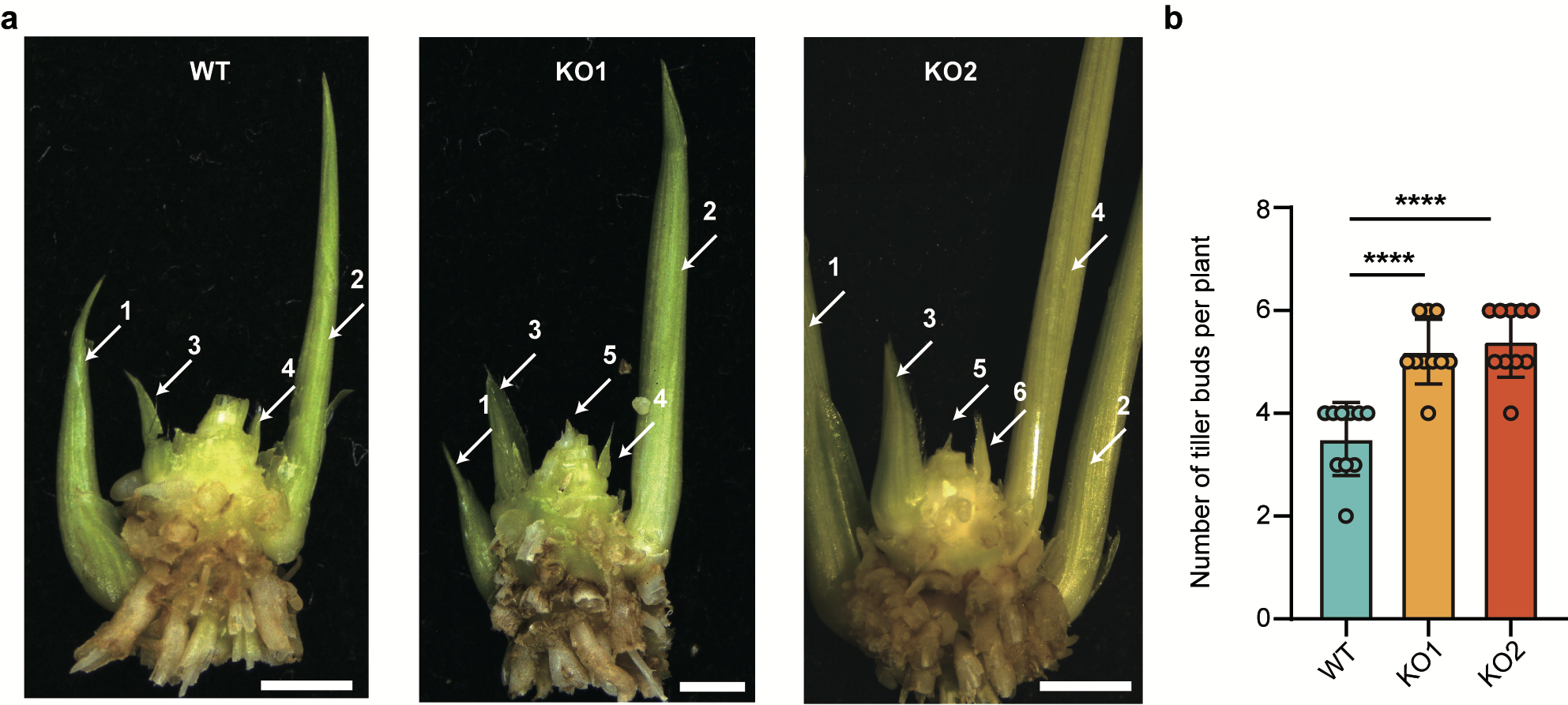


**Fig. S8 *OsNOP2* knockout shows simultaneously increased number and size of tiller buds.** **a**, Phenotypes of tiller buds in 2-month-old seedlings from WT, KO1, and KO2 plants. Scale bar, 1 mm. **b**, The number of tiller buds of WT, KO1, and KO2 plants were counted. Data are mean ± S.D. (n = 10 plants). *****P* < 0.0001; *P* values are from one-way ANOVA (and nonparametric or mixed).


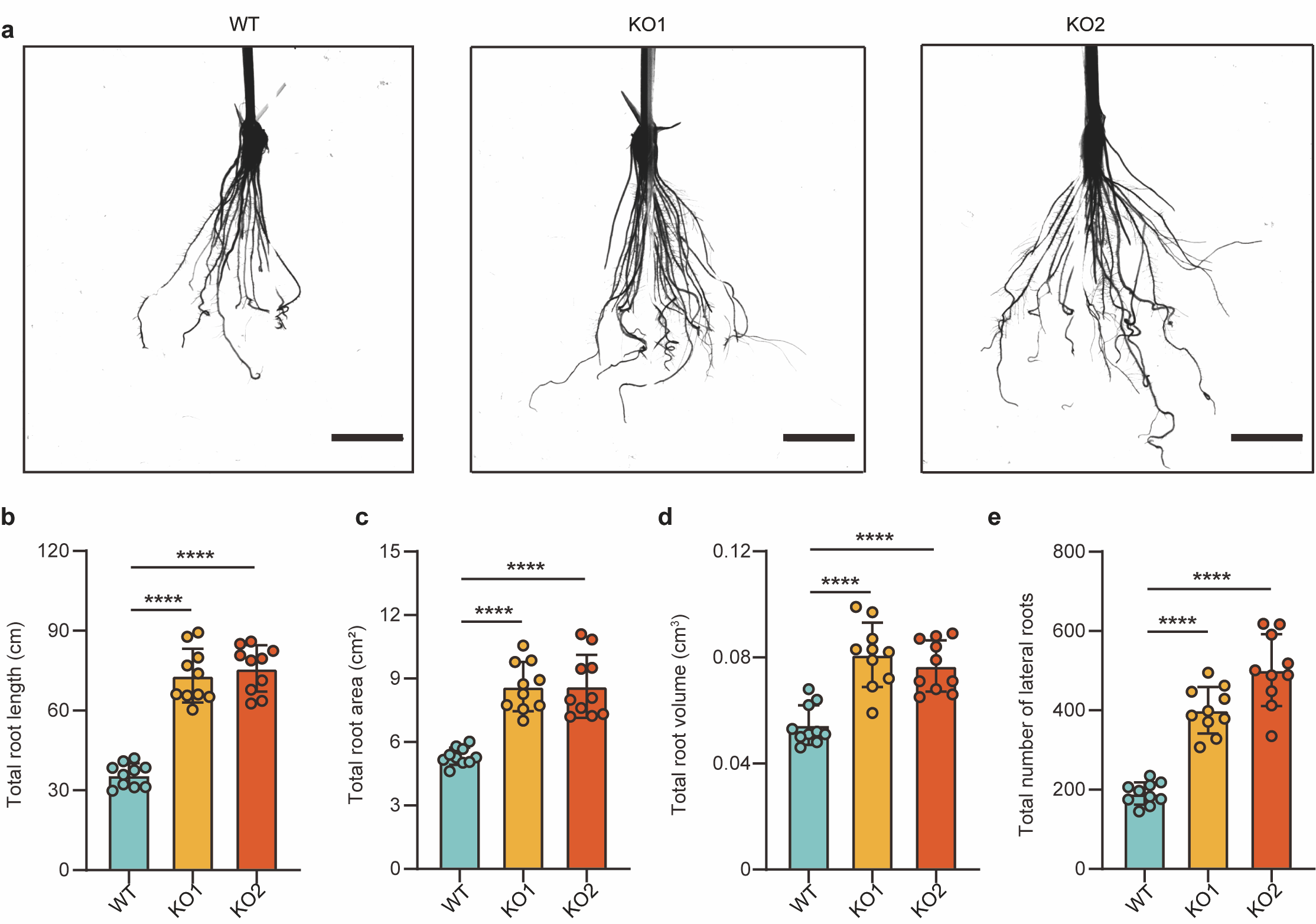


**Fig. S9 *OsNOP2* knockout improves the phenotype and relevant parameters of root morphological. a**, The scan images of WT, KO1, and KO2 roots at seedling stage hydroponically grown in the greenhouse by using EPSON scanner (Epson Expression 1600pro). Scale Bar, 2 cm. **b-e**, Statistical analysis of total root length (**b**), total root area (**c**), total root volume (**d**)**,** and total number of lateral roots (**e**) of WT, KO1, and KO2 seedlings by using WinRHIZO software (Regent Instruments). Data are mean ± S.D. (**b-e**, n = 10 plants). *****P* < 0.0001; *P* values are from one-way ANOVA (and nonparametric or mixed).


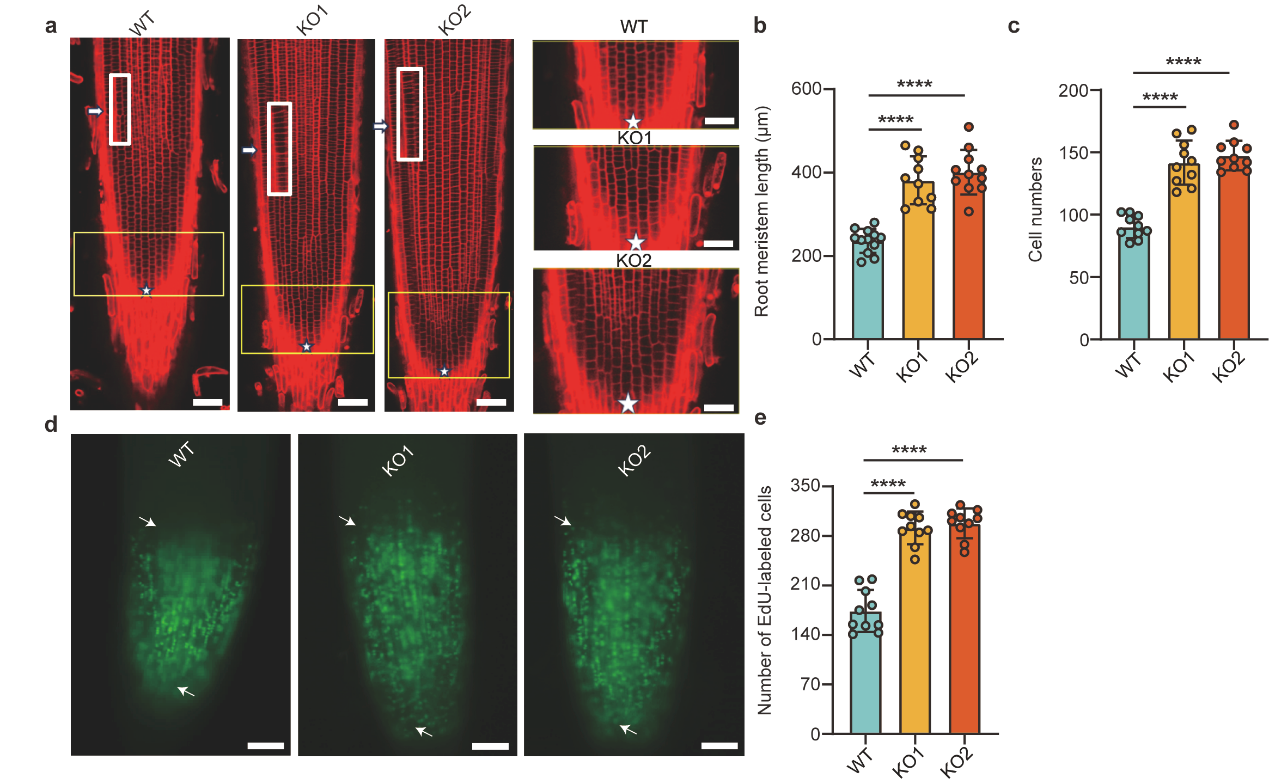


**Fig. S10 *OsNOP2* knockout positively regulates root meristem cell proliferation. a**, PI staining of root tips in 3-day-old WT, KO1, and KO2 rice seedlings. **b-c**, Quantitative analysis of root meristem length (**b**) and cell numbers (**c**) within the 1/3 length of the root meristem. Arrowheads and asterisks indicate range of roots meristems and the first enlarged cell is shown in the magnified view within the white box. Cell numbers were calculated from the 1/3 length of the root meristem as indicated by the yellow boxes and magnified on the right. Scale bar, 50 μm. **d**, Longitudinal view of EdU-labeled cells in root meristems of 3-day-old WT, KO1, and KO2 rice seedlings. Scale bar, 50 μm. **e**, Statistical analysis of numbers of EdU-labeled cells in root tips were counted. Arrowheads indicate range of roots meristems. Data are mean ± S.D. (**b**, n = 10 plants; **c**, n = 10 cells; **e**, n = 10 plants). *****P* < 0.0001; *P* values are from one-way ANOVA (and nonparametric or mixed).


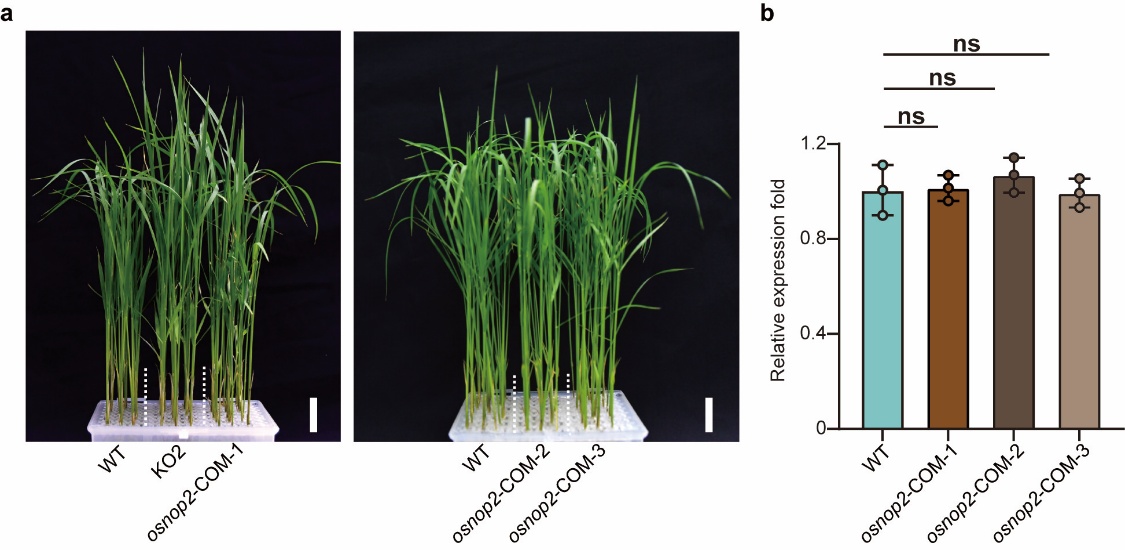


**Fig. S11 Comparison of *OsNOP2* transcript levels and phenotype between complementation, knockout, and wild-type lines. a**, Phenotypes of three-week-old WT, *OsNOP2*-KO2, and *OsNOP2*-COM (three independent COM lines) seedlings grown under hydroponic conditions. Scale bar, 5 cm. **b**, Relative expression levels of *OsNOP2* in the WT, KO2, and COM lines. Data are means ± S.D. (**b**, n = 3 biological replicates). ns, no significance; *P* values were determined by one-way ANOVA (and nonparametric or mixed).


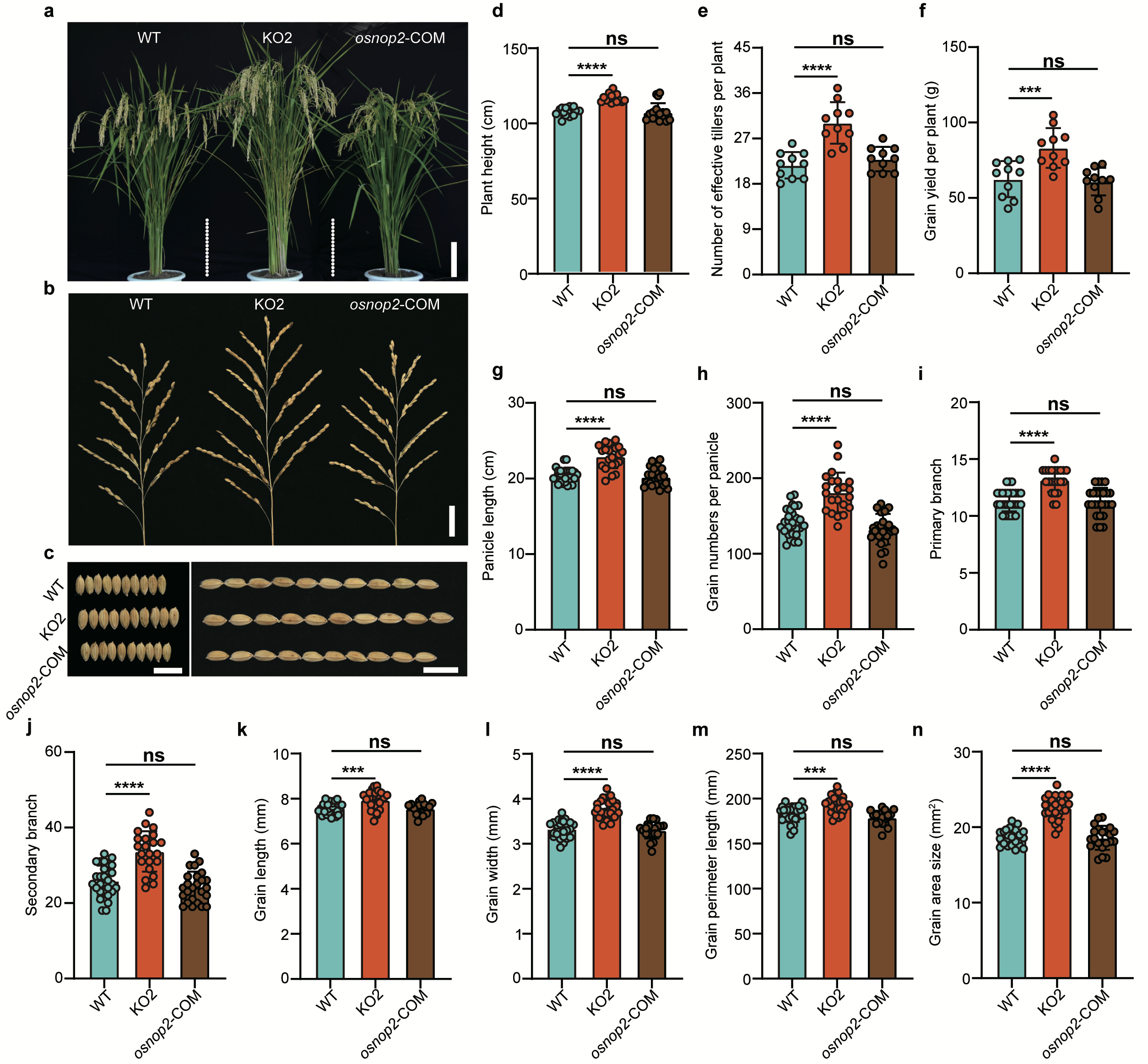


**Fig. S12 *OsNOP2* complementation plants rescue the *OsNOP2* knockout phenotype. a**-**c**, Morphology (**a**), panicle morphology (**b**), and grain morphology (**c**) of WT, KO2, and *osnop2*-COM plants. Scale bars, 10 cm (**a**), 3 cm (**b**), and 1 cm (**c**). **d-n**, Statistical analysis of plant height (**d**), number of effective tillers per plant (**e**), grain yield per plant (**f**), panicle length (**g**), grain numbers per panicle (**h**), primary branch (**i**), secondary branch (**j**), grain length (**k**), grain width (**l**), grain perimeter length (**m**), and grain area size (**n**) in WT, KO2, and *osnop2*-COM plants. Data are mean ± S.D. (**d-f**, n = 10 plants; **g-j**, n ≥ 20 panicles; **k-n**, n ≥ 20 grains). ns, no significance, ****P* < 0.001, *****P* < 0.0001; *P* values are from one-way ANOVA (and nonparametric or mixed).


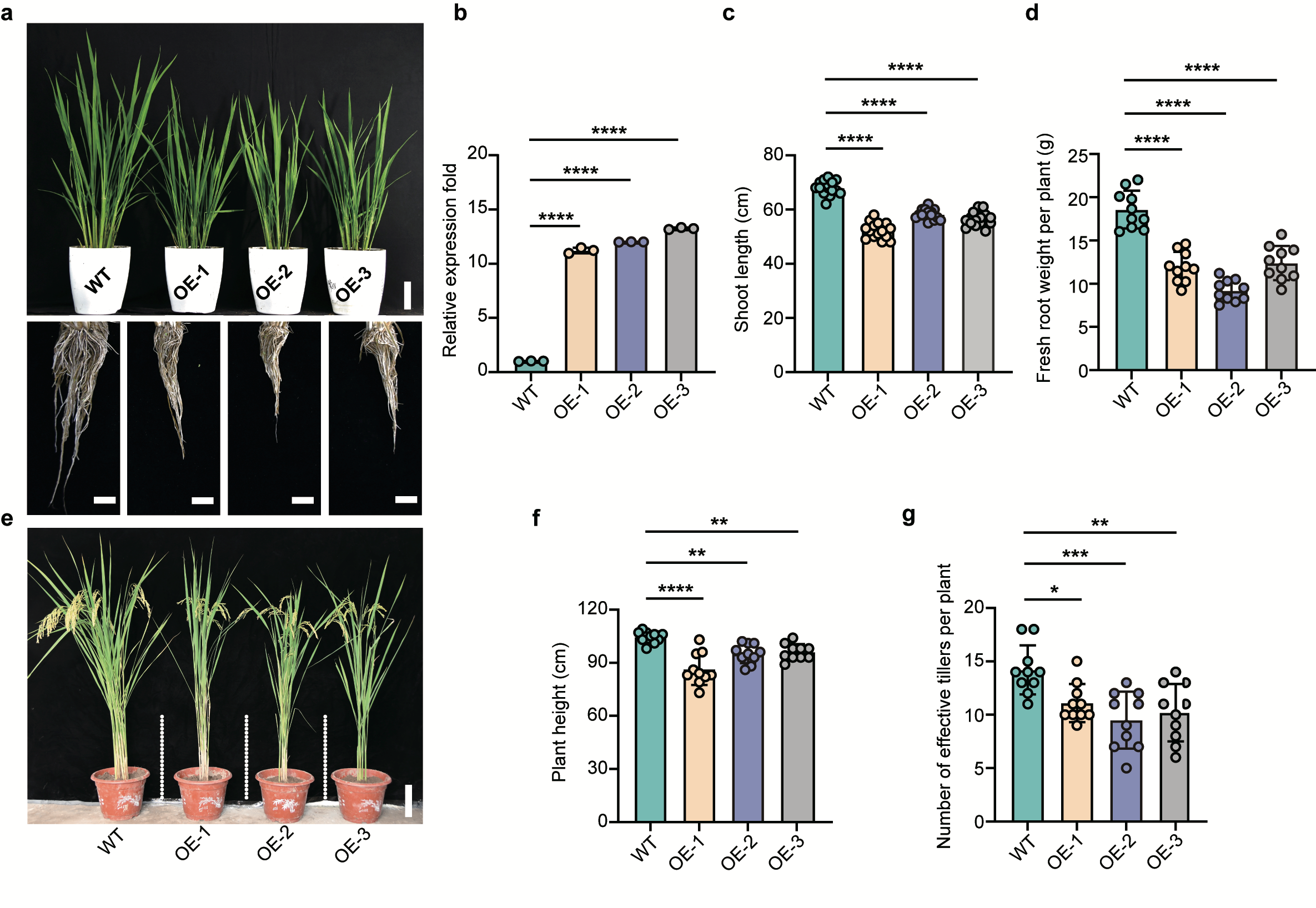


**Fig. S13 *OsNOP2* overexpression decreases the shoot length and the fresh root weight at tillering stage, and decreases the plant height and the number of effective tillers per plant at grain-filling stage. a**, Phenotypes of shoot and root in tillering-stage seedlings of WT, OE1, OE2, and OE3 plants. Scale bar, 5 cm. **b**, Relative expression levels of *OsNOP2* in the WT and *OsNOP2*-overexpressing plants (OE-1, OE-2, and OE-3). **c-d**, Statistical analysis of shoot length (**c**) and fresh root weight per plant (**d**) of WT, OE1, OE2, and OE3 plants were measured. **e**, Morphology of WT, OE1, OE2, and OE3 plants at the grain-filling stage. Scale bar, 10 cm. **f-g**, Statistical analysis of plant height (**f**) and the number of effective tillers per plant (**g**) from WT, OE1, OE2, and OE3 plants. Data are mean ± S.D. (**b**, n = 3 biological replicates; **c-d**, and **f-g**, n = 10 plants). **P* < 0.05, ***P* < 0.01, ****P* < 0.001, *****P* < 0.0001; *P* values are from one-way ANOVA (and nonparametric or mixed).


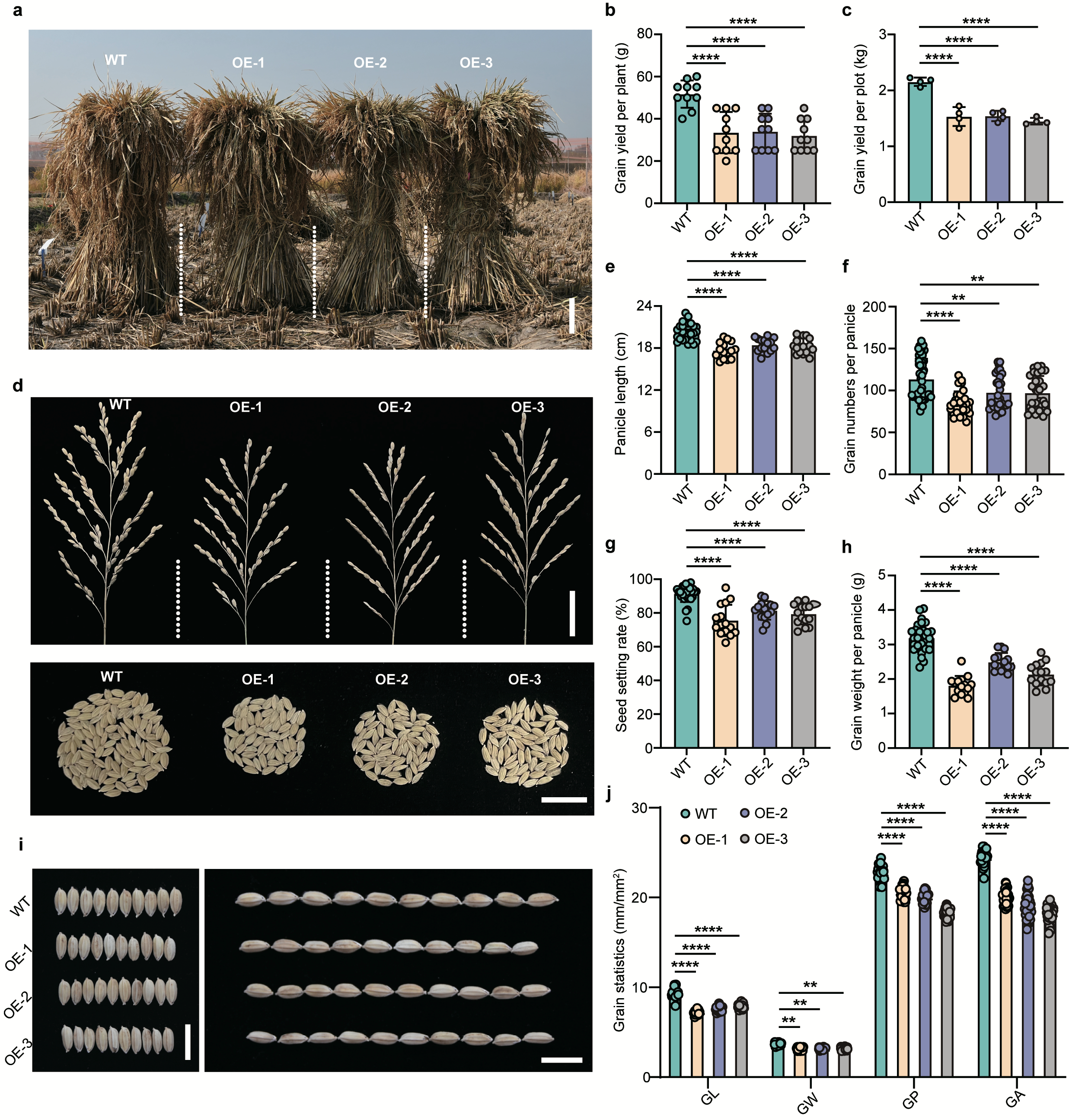


**Fig. S14 *OsNOP2* overexpression in WT plants slump grain yield. a**, Phenotypes of plot yield from WT, OE1, OE2, and OE3 plants. Scale bar, 15 cm. **b-c**, Statistical analysis of grain yield per plant (**b**) and grain yield per plot (**c**) in WT, OE-1, OE-2, and OE-3 plants. **d**, Phenotypes of panicle from WT, OE-1, OE-2, and OE-3 plants. Scale bars, 3 cm (top) and 1 cm (bottom). **e-h**, Statistical analysis of panicle length (**e**), grain numbers per panicle (**f**), seed setting rate (**g**), and grain weight per panicle (**h**) from WT, OE-1, OE-2, and OE-3 plants. **i**, Grain morphologies of WT, OE-1, OE-2, and OE-3 plants. Scale bar, 1 cm. **j**, Statistical analysis of grain length (GL), grain width (GW), grain perimeter length (GP), and grain area size (GA) of WT, OE-1, OE-2, and OE-3 plants. Data are mean ± S.D. (**b**, n = 10 plants; **c**, n = 4 paddies, each paddy contained 8 × 15 plants in the 4.5-m^2^ field; **e-h**, n = 15 panicles; **j**, n > 30 grains). ***P* < 0.01, *****P* < 0.0001; *P* values are from one-way ANOVA (and nonparametric or mixed).


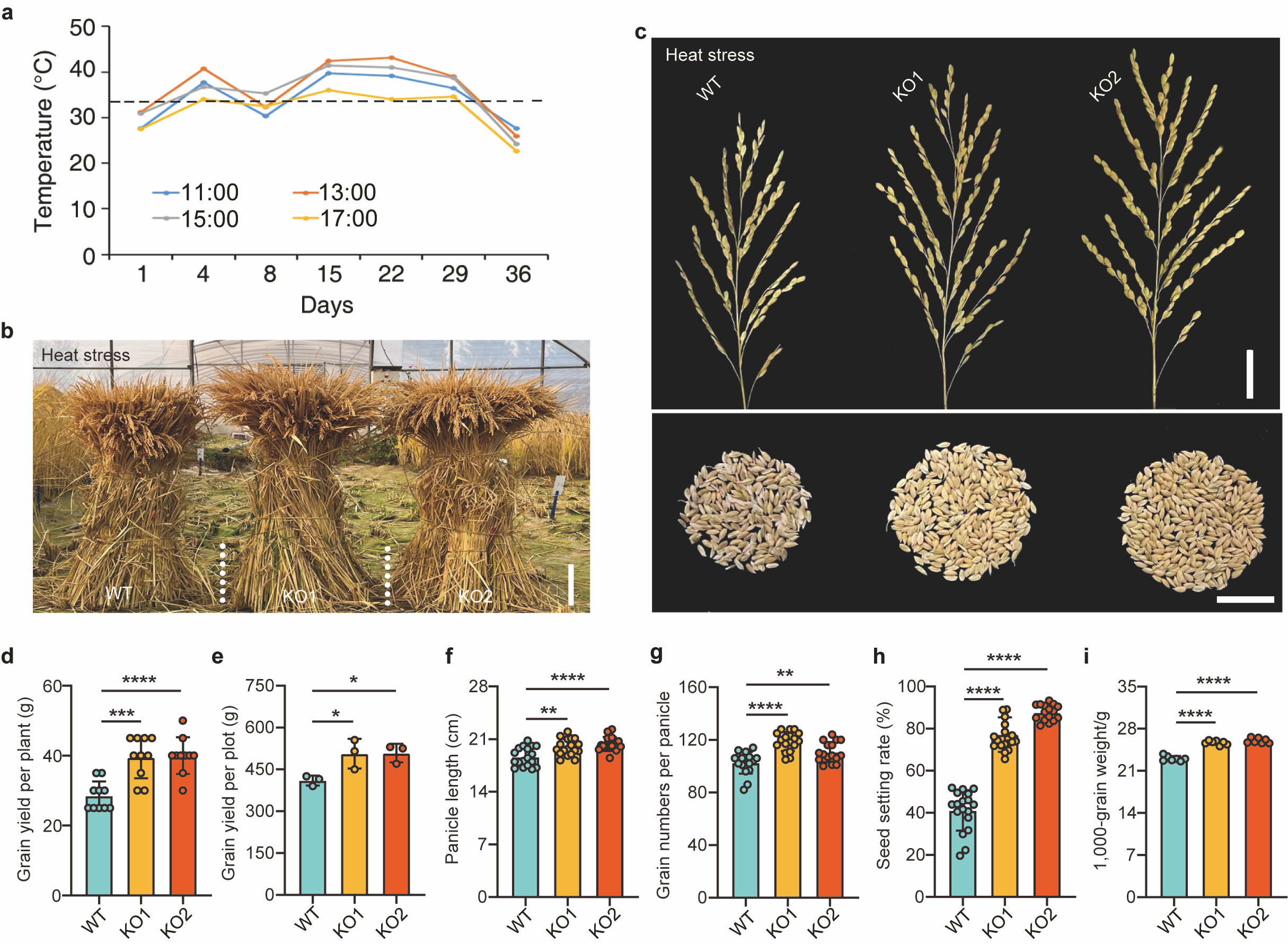


**Fig. S15 *OsNOP2* knockout maintains high grain yield under heat stress. a**, Temperature recording from the greenhouse covered with plastic film at the field was monitored. Records were collected every 2 h from 11:00 to 17:00. The black dashed line indicates the temperature of 38°C. **b-c**, Phenotypes of plot yield (**b**) and panicle (**c**) after one month of heat stress at the heading stage from WT, KO1, and KO2 plants. Scale bars, 15 cm (**b**) and 3 cm (top) and 1 cm (bottom) (**c**). **d-i**, Statistical analysis of grain yield per plant (**d**), grain yield per plot (**e**), panicle length (**f**), grain numbers per panicle (**g**), seed setting rate (**h**), and 1,000-grain weight (**i**) in WT, KO1, and KO2 plants. Data are mean ± S.D. (**d**, n = 15 plants; **e**, n = 3 paddies, each paddy contained 10 × 6 plants in the 1.8-m^2^ field; **f-h**, n = 20 panicles; **i**, n = 7 biological replicates). **P* < 0.05, ***P* < 0.01, *****P* < 0.0001; *P* values are from one-way ANOVA (and nonparametric or mixed).
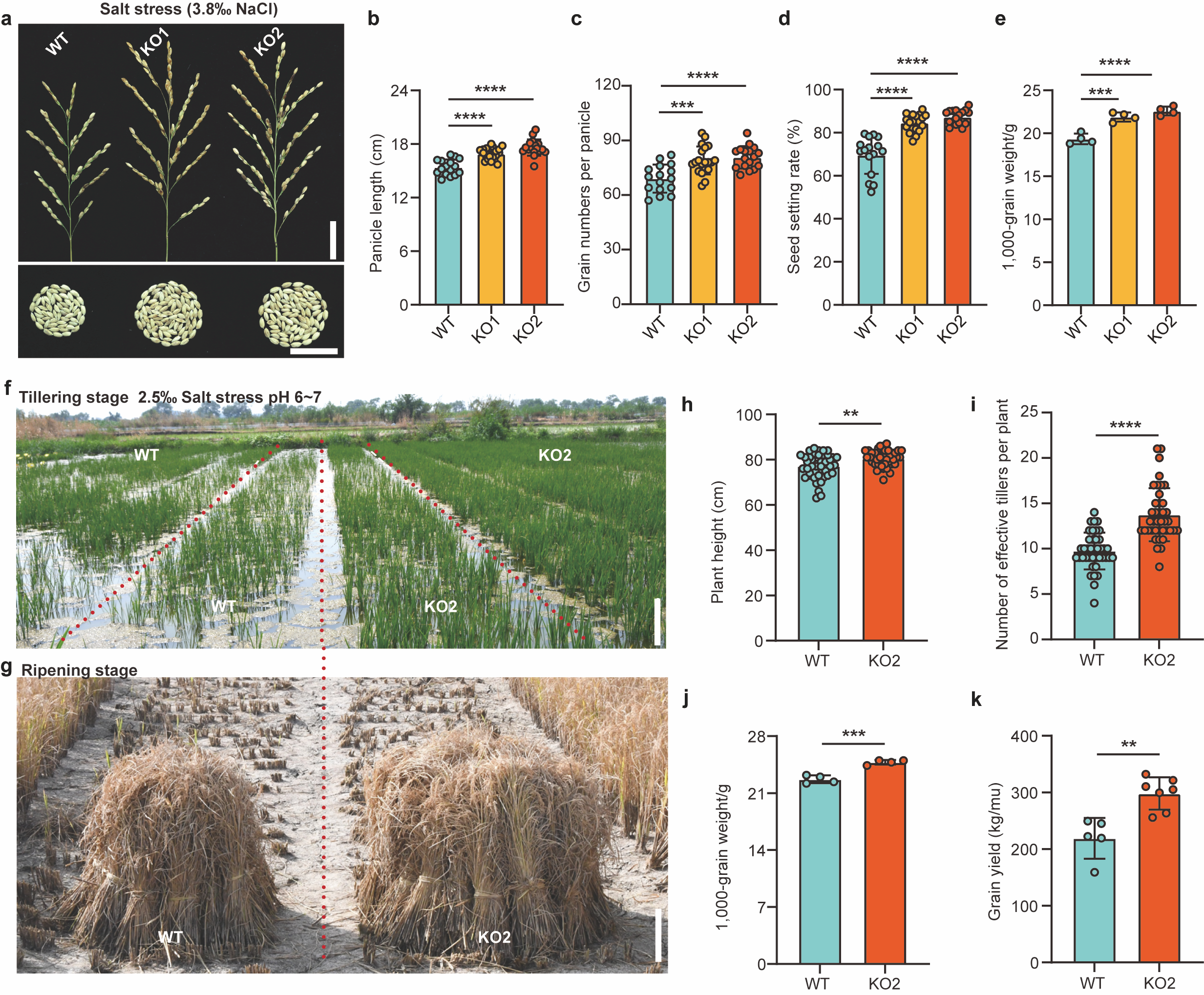


**Fig. S16 *OsNOP2* knockout remains high grain yield exposed to salt stress. a**, Phenotypes of panicle from WT, KO1, and KO2 plants grown uniformly in the field supplemented with 3.8‰ NaCl from seedling stage to ripening stage in Dongying. Scale bar, 3 cm (top) and 1 cm (bottom). **b-e**, Statistical analysis of panicle length (**b**), grain numbers per panicle (**c**), seed setting rate (**d**) and 1,000-grain weight (**e**) in WT, KO1, and KO2 plants. **f**, **g**, Field phenotypes of WT and KO2 plants grown uniformly in the field supplemented with 2.5‰ NaCl at tillering stage (**f**) and ripening stage (**g**) in Wuqing. Scale bars, 15 cm (**f**) and 20 cm (**g**). **h-k**, Statistical analysis of the plant height (**h**), number of effective tillers per plant (**i**), 1,000-grain weight (**j**), and grain yield per mu (**k**) in WT and KO2 plants. Data are mean ± S.D. (**b-d**, n ≥ 15 panicles; **e**, n = 4 biological replicates; **h-i**, n ≥ 20 plants; **j**, n = 4 biological replicates; **k**, n = 5 biological replicates). ***P* < 0.01, ****P* < 0.001, *****P* < 0.0001; *P* values are from one-way ANOVA (and nonparametric or mixed).


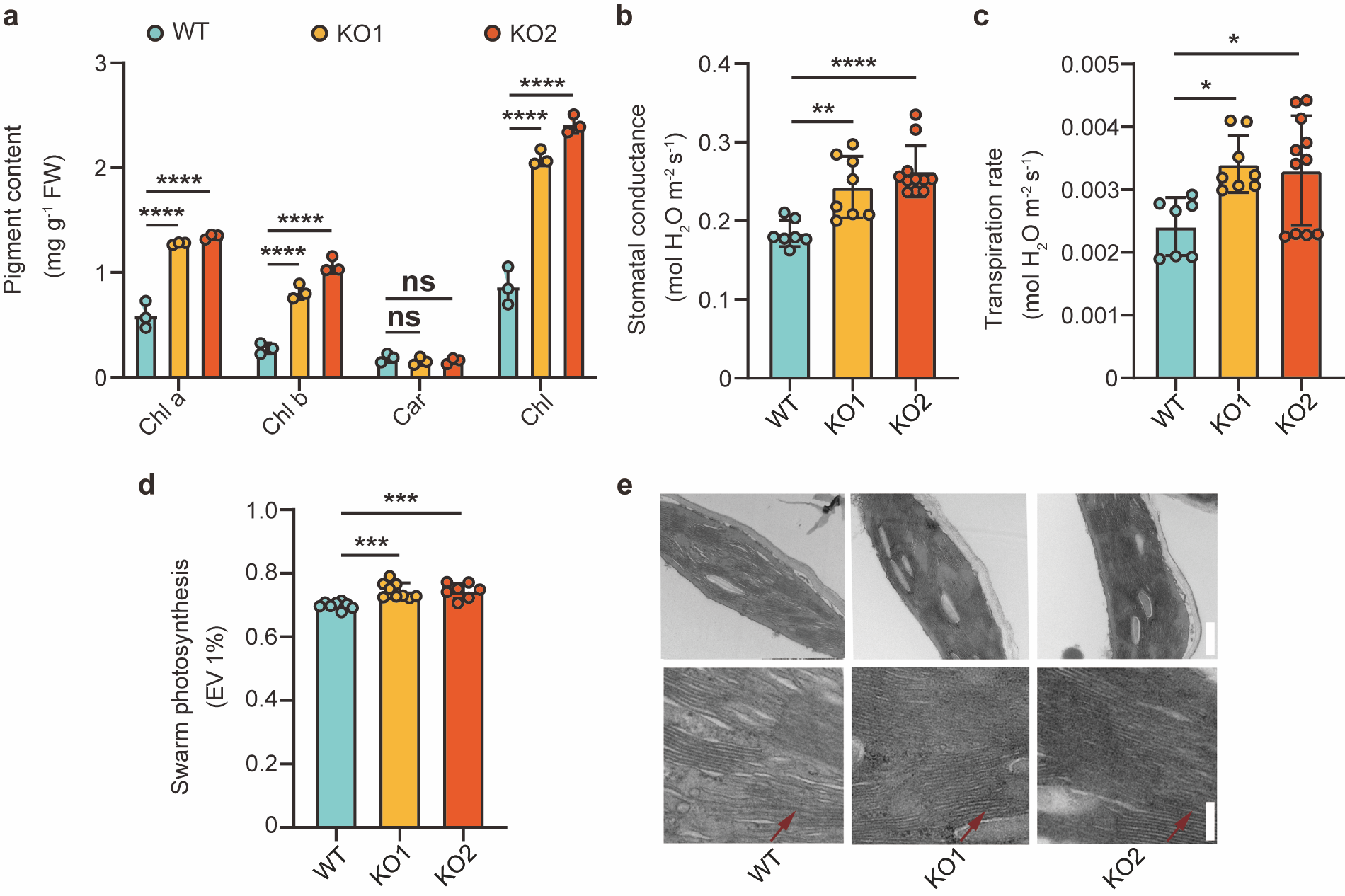


**Fig. S17 *OsNOP2* knockout regulates photosynthetic capacity. a-d**, Statistical analysis of the pigment content (**a**), stomatal conductance (**b**), transpiration rate (**c**), and swarm photosynthesis (EV1%) (**d**) in WT, KO1, and KO2 plants. Chlorophyll a (Chl a), chlorophyll b (Chl b), carotenoid (Car), and total Chlorophyll (Chl) were extracted with acetone and determined by spectrophotometry. **e**, Transmission electron micrographs of chloroplast ultrastructure from leaves of WT, KO1, and KO2 plants. Scale bar, 200 nm. The red arrow indicates collapsed thylakoids. Data are mean ± S.D. (**a**, n = 3 biological replicates; **b-d**, n ≥ 7 plants). ns, no significance, **P* < 0.05, ***P* < 0.01, ****P* < 0.001, *****P* < 0.0001. In **a**, *P* values are from two-way ANOVA (and nonparametric or mixed). In **b-d**, *P* values are from one-way ANOVA (and nonparametric or mixed).


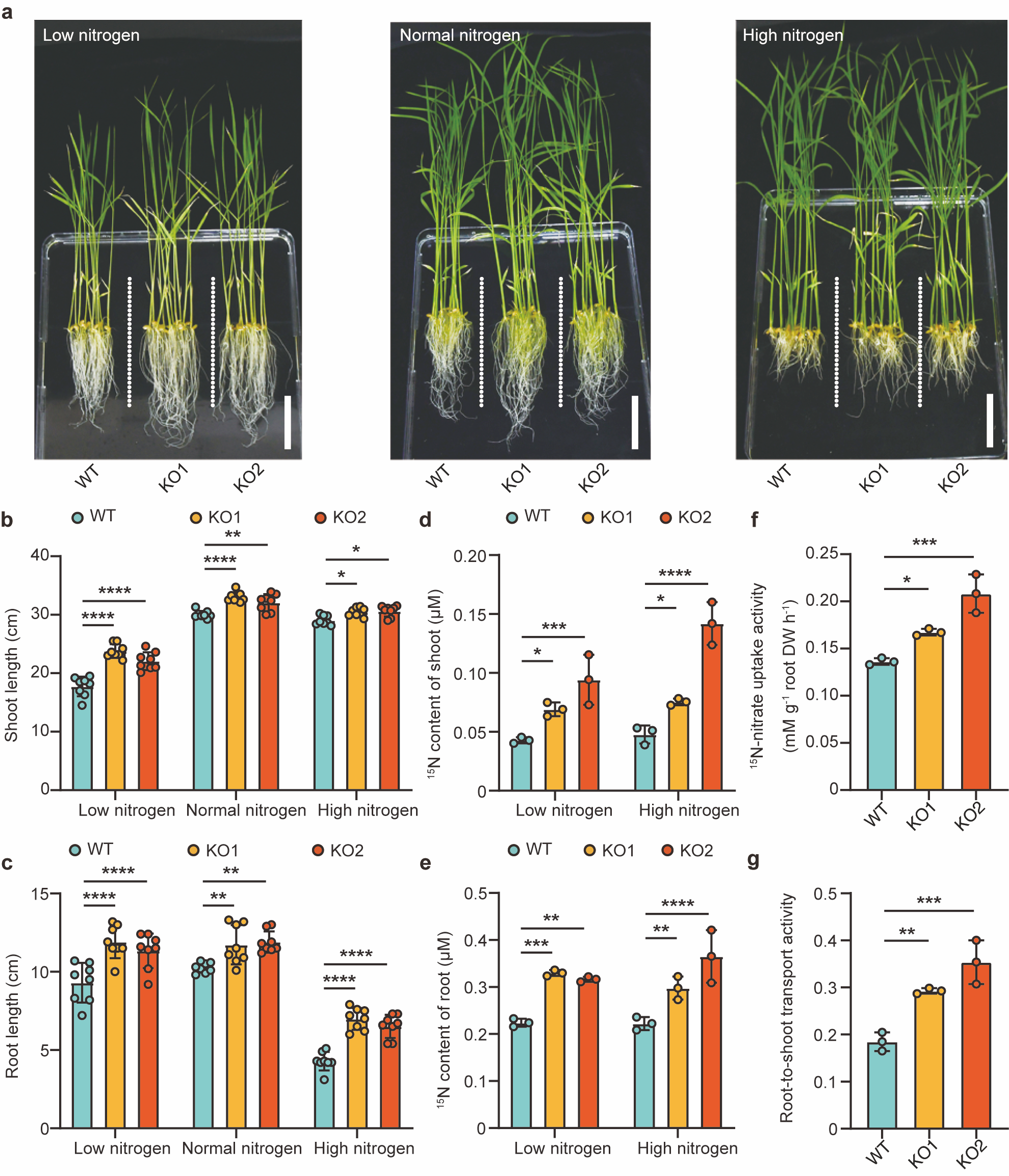


**Fig. S18 *OsNOP2* knockout positively regulates nitrogen response. a**, Phenotypes of WT, KO1, and KO2 under low nitrogen (1 mM NH_4_NO_3_), normal nitrogen (1.425 mM NH_4_NO_3_), and high nitrogen (2 mM NH_4_NO_3_). Scale bar, 5 cm. **b-c**, Statistical analysis of the shoot length (**b**) and root length (**c**) in WT, KO1, and KO2 under various nitrate conditions. **d-e**, Statistical analysis of ^15^N content of shoot (**d**) and ^15^N content of root (**e**) in WT, KO1, and KO2 plants under low nitrogen and high nitrogen conditions. **f-g**, Statistical analysis of the ^15^N-nitrate uptake activity (**f**) and the shoot-to-root transport activity (**g**) from WT, KO1, and KO2 plants. Data are mean ± S.D. (**b-c**, n = 8 plants; **d-g**, n = 3 biological replicates). **P* < 0.05, ***P* < 0.01, ****P* < 0.001, *****P* < 0.0001; In **b-e**, *P* values are from two-way ANOVA (and nonparametric or mixed). In **f-g**, *P* values are from one-way ANOVA (and nonparametric or mixed).


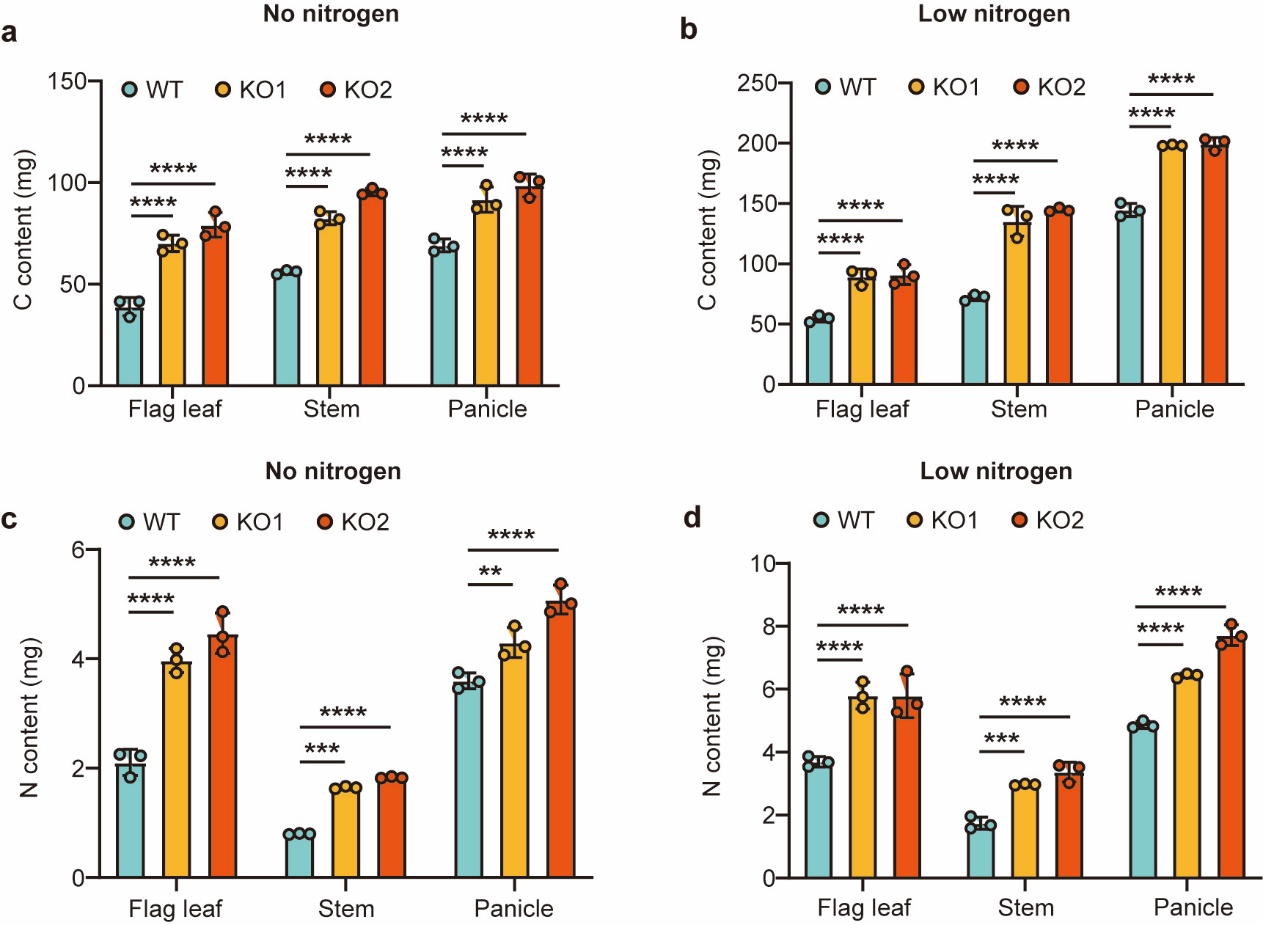


**Fig. S19** **Elevated carbon and nitrogen contents across tissues in *OsNOP2* knockout lines. a-b**, Statistical analysis of carbon (C) contents in WT, *OsNOP2-*KO1, and -KO2 plants at filling stage under field conditions with no nitrogen (N) (0 kg ha^-1^) (**a**) or low N (112.5 kg ha^-1^) supplementation (**b**). **c-d**, Statistical analysis of N contents in WT, KO1, and KO2 plants at filling stage under field conditions with no N (0 kg ha^-1^) (**c**) or low N (112.5 kg ha^-1^) supplementation (**d**). Data are means ± S.D. (n = 3 biological replicates). ***P* < 0.01, ****P* < 0.001, *****P* < 0.0001; *P* values are from two-way ANOVA (and nonparametric or mixed).


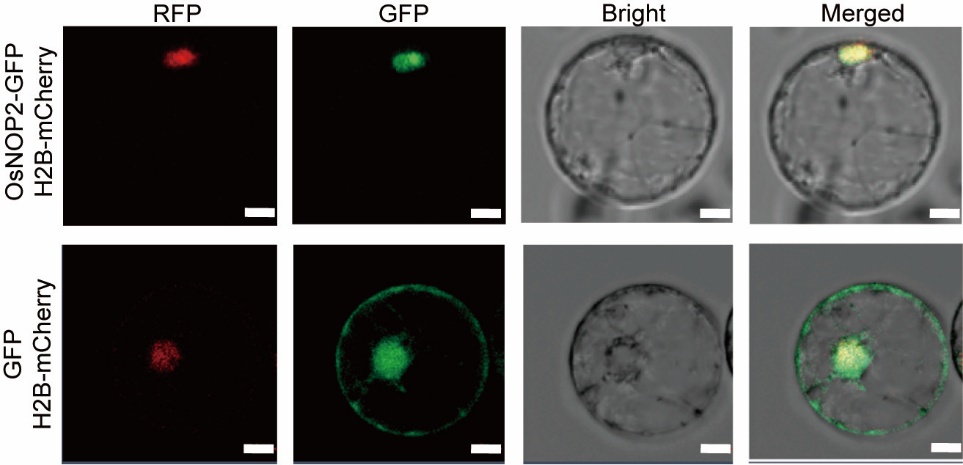


**Fig. S20 Subcellular localization of OsNOP2.** Nuclear localization of OsNOP2 in rice protoplasts. The empty vector pAN580 (GFP) was used as negative control. The nuclear protein H_2_B fused with mCherry (RFP) was used as a nuclear marker. Scale bar, 20 μm.


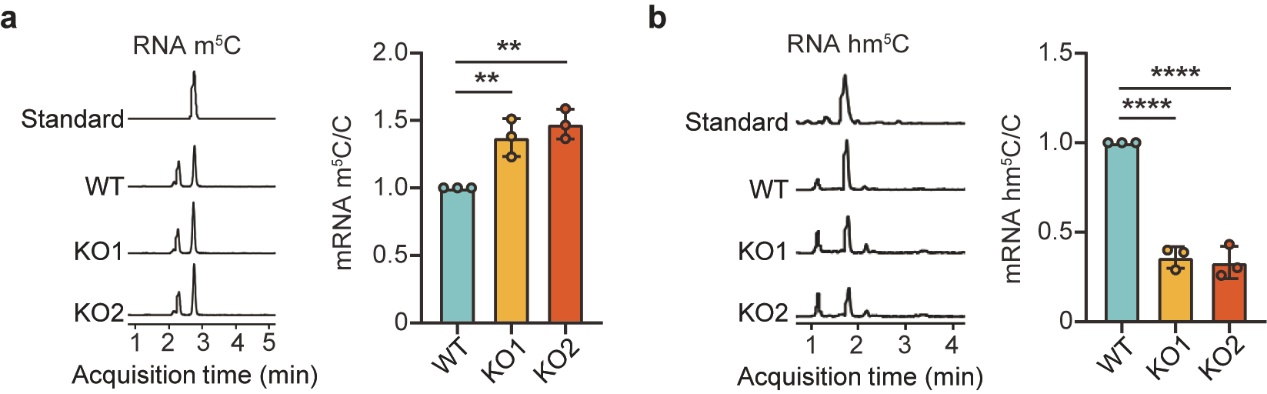


**Fig. S21** **OsNOP2 functions as an mRNA m^5^C demethylase in rice.** **a**, Detection of mRNA m^5^C levels in 3-week-old hydroponically grown WT, *OsNOP2*-KO1, and -KO2 seedlings by LC-MS/MS. **b**, Detection of mRNA hm^5^C levels in 3-week-old hydroponically grown WT, KO1, and KO2 seedlings by LC-MS/MS. Data are means ± S.D. (n = 3 biological replicates). ***P* < 0.01, *****P* < 0.0001; *P* values are from one-way ANOVA (and nonparametric or mixed).


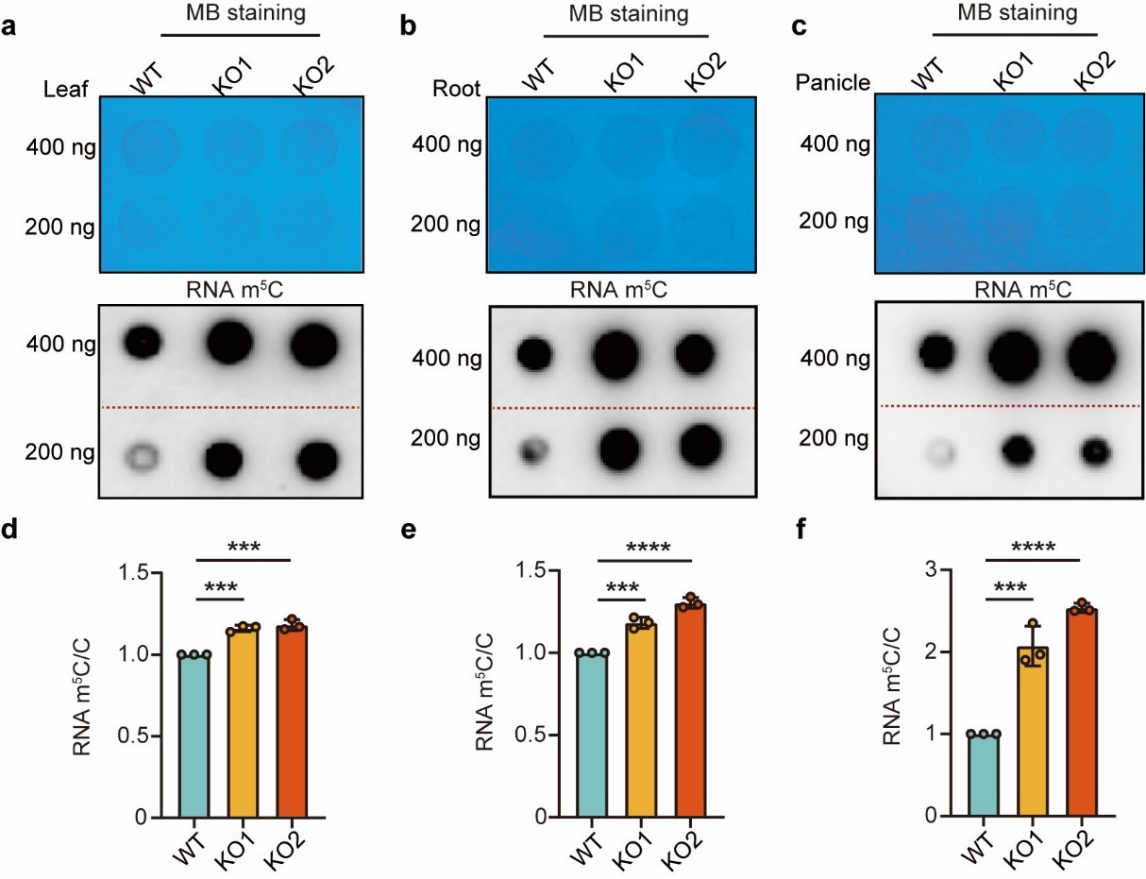


**Fig. S22 Detection of total RNA m^5^C level in different tissues. a-c**, Detection of total RNA m^5^C level of leaf (**a**), root (**b**), and panicle (**c**) in WT, KO1, and KO2 plants by dot blot. **d-f**, Detection of total RNA m^5^C level of leaf (**d**), root (**e**), and panicle (**f**) in WT, KO1, and KO2 plants by LC-MS/MS. Data are mean ± S.D. (**a-f**, n = 3 biological replicates). ***P* < 0.01, ****P* < 0.001; *P* values are from one-way ANOVA (and nonparametric or mixed).


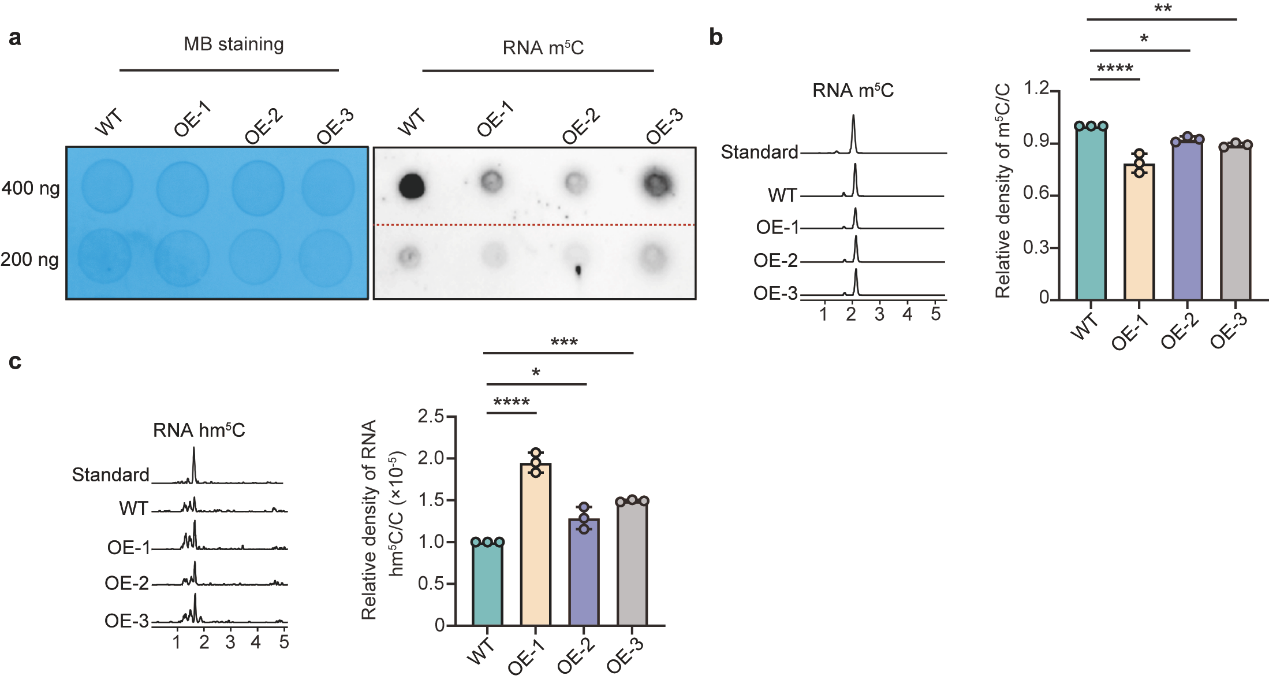


**Fig. S23 Detection of total RNA m^5^C levels in WT and *OsNOP2* overexpression plants. a**, Detection of total RNA m^5^C levels in WT and *OsNOP2* overexpression plants (OE-1, OE-2, and OE-3) by dot blot. **b-c**, LC–MS/MS assay showed detection and quantification of RNA m^5^C levels (**b**) and RNA hm^5^C (**c**) in WT and *OsNOP2* overexpression plants (OE-1, OE-2, and OE-3). Data are mean ± S.D. (n = 3 biological replicates). **P* < 0.05, ***P* < 0.01, ****P* < 0.001, *****P* < 0.0001; *P* values are from one-way ANOVA (and nonparametric or mixed).


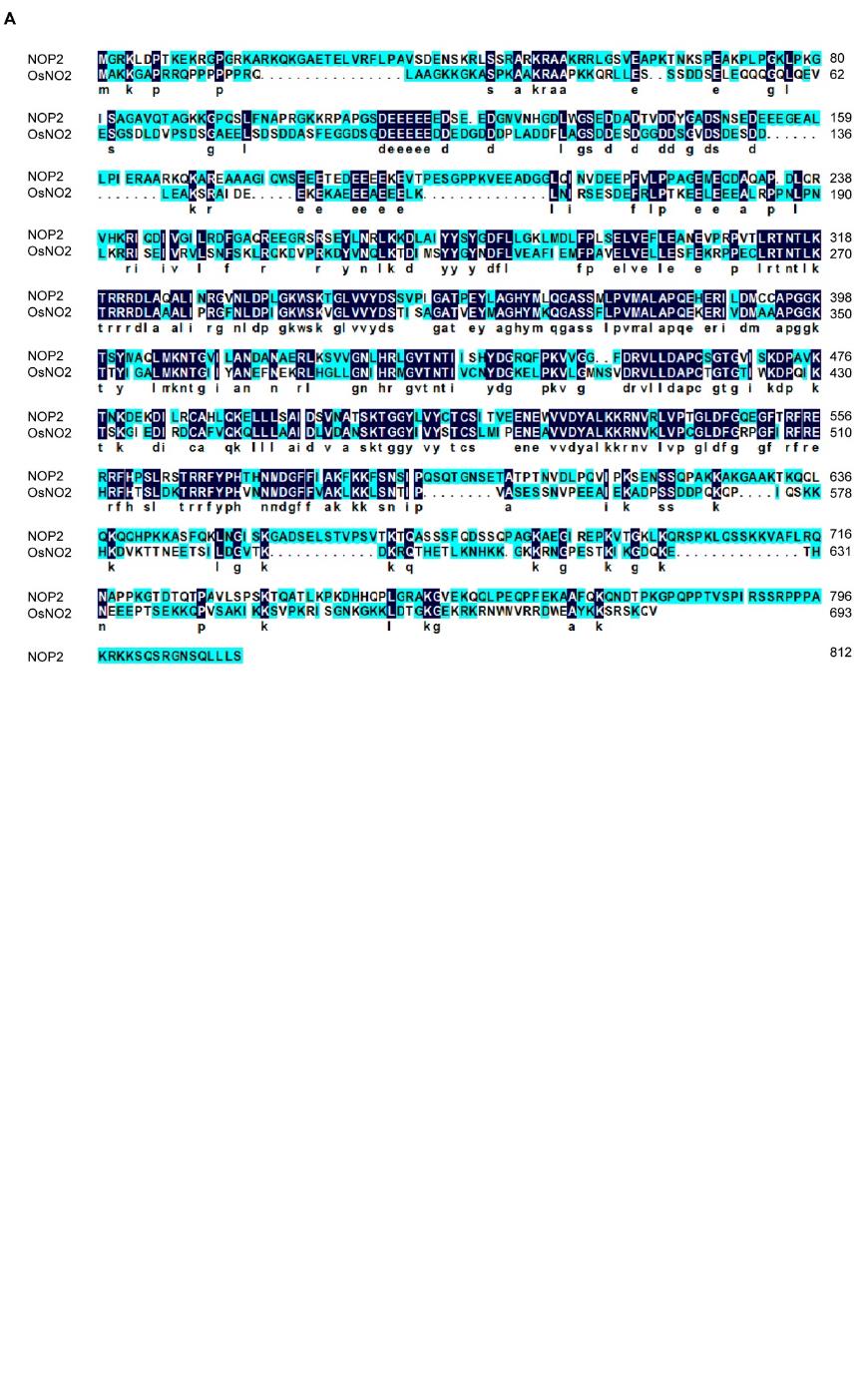


**Fig. S24 Comparison of the protein sequence of OsNOP2 and NOP2.**


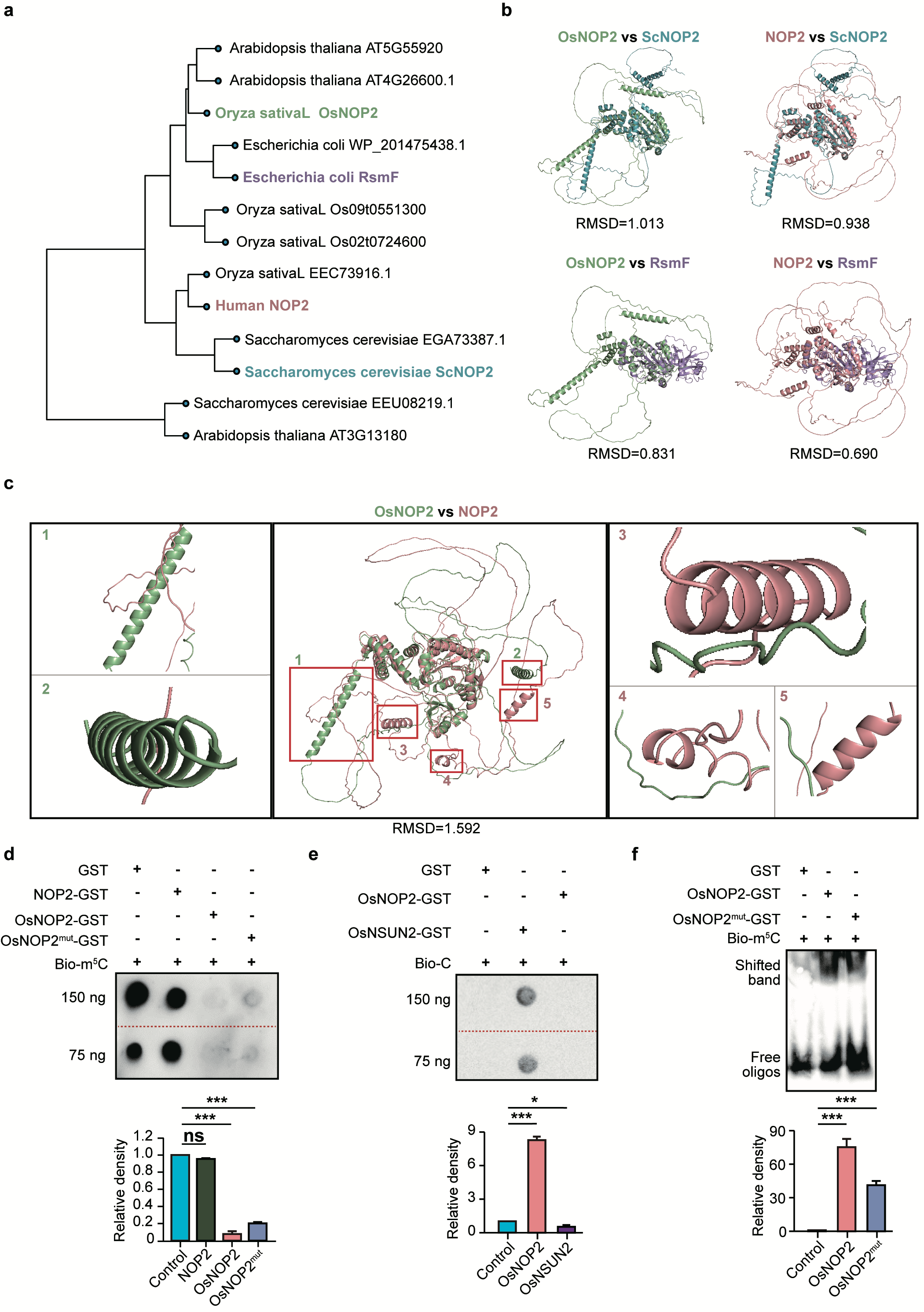


**Fig. S25 Comparison of the structures between NOP2 and OsNOP2. a**, Evolutionary tree displaying NOL1/NOP2/Sun family protein across different species. **b**, Comparison of the structures of OsNOP2 and NOP2 with ScNOP2 and RsmF proteins, respectively. **c**, Comparison of the structures of NOP2 and OsNOP2. **d**, The detection of demethylase activity of NOP2, OsNOP2, and OsNOP2^mut^ *in vitro* by dot blot, and the quantification was shown below the figure. **e**, The detection of methyltransferase activity of OsNOP2 and OsNSUN2 *in vitro* by dot blot, and the quantification was shown below the figure. **f**, The binding ability of OsNOP2 proteins to motif sequence with m^5^C modification by EMSA assay, and the quantification was shown below the figure. Data are mean ± S.D. (**d-f**, n = 3 biological replicates). ns, no significance, **P* < 0.05, ****P* < 0.001; *P* values are from two-tailed Student’s t-tests.

**
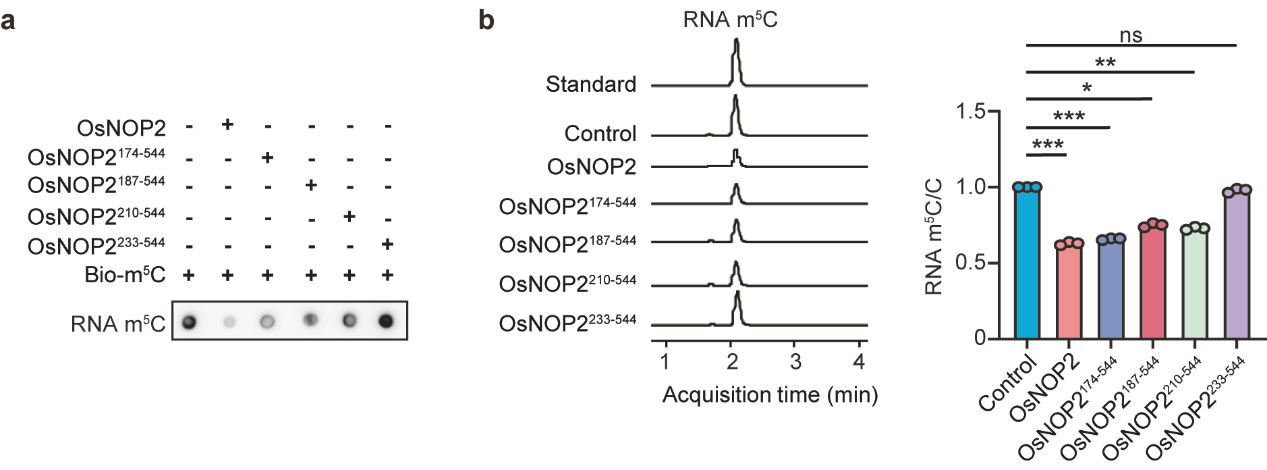
**

**Fig. S26 Assessment of m^5^C demethylation activity by OsNOP2 truncation fragments expressed in eukaryotic cells. a-b**, Dot blot (**a**) and LC-MS/MS (**b**) detection of residual RNA m^5^C levels in reactions containing Bio-m^5^C and full-length OsNOP2 or truncation fragments purified from Spodoptera frugiperda cells. Data are means ± S.D. (n = 3 biological replicates). **P* < 0.05, ***P* < 0.01, ****P* < 0.001, *****P* < 0.0001; *P* values are from one-way ANOVA (and nonparametric or mixed).


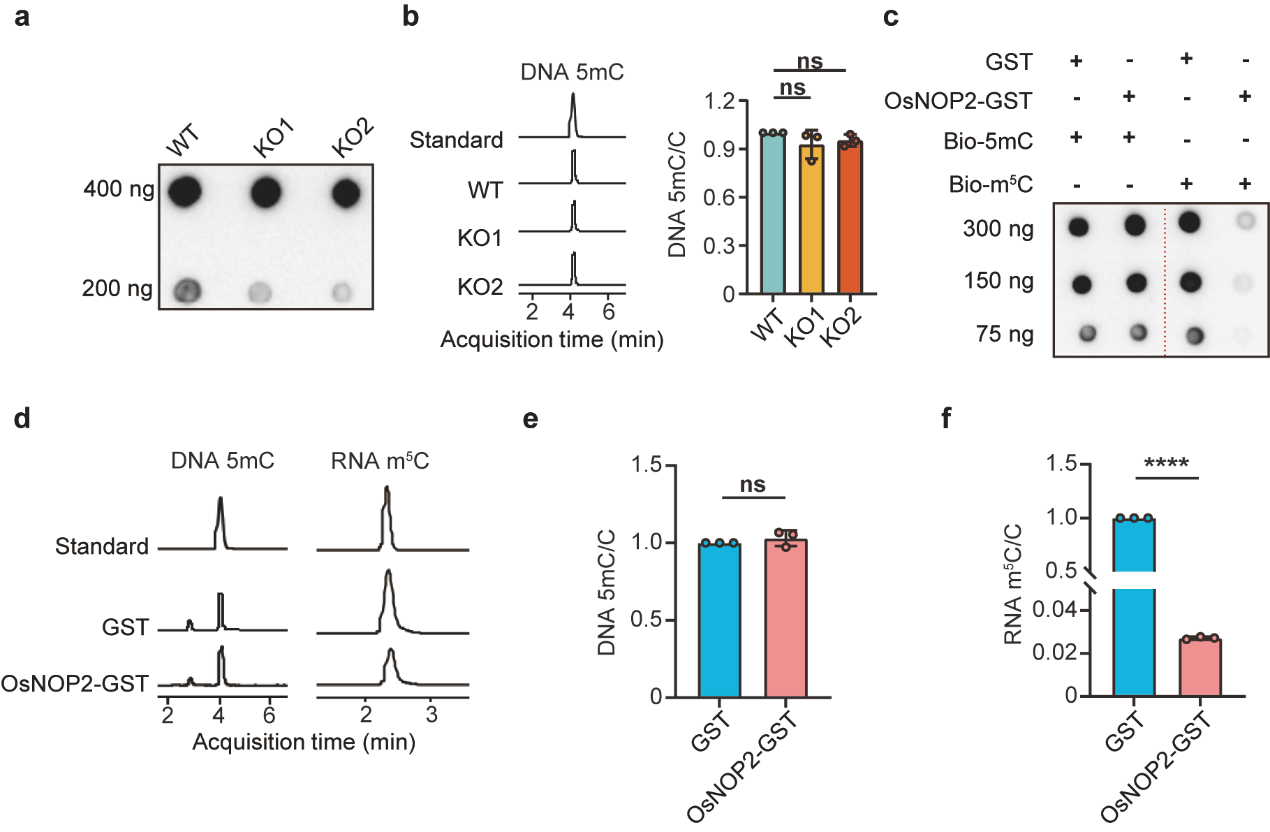


**Fig. S27 OsNOP2 exhibits no DNA 5mC demethylase activity *in vivo* or *in vitro*. a-b**, Dot blot (**a**) and LC-MS/MS (**b**) detection of genomic DNA 5mC levels in hydroponically grown 3-week-old WT, *OsNOP2*-KO1, and -KO2 seedlings. **c-f**, *In vitro* detection of DNA 5mC levels. **c**, Dot-blot assays 5mC relative abundance following OsNOP2-GST exposure to different concentrations of Bio-5mC substrate; signal intensity reflects residual DNA 5mC contents. The red dashed line separates the DNA 5mC substrate on the left from the RNA m^5^C substrate on the right. **d-f**, LC-MS/MS quantification of residual DNA 5mC contents (**e**) and RNA m^5^C contents (**f**) under the same condition. Data are means ± S.D. (n = 3 biological replicates). ns, no significance, *****P* < 0.0001; *P* values are from one-way ANOVA (and nonparametric or mixed).


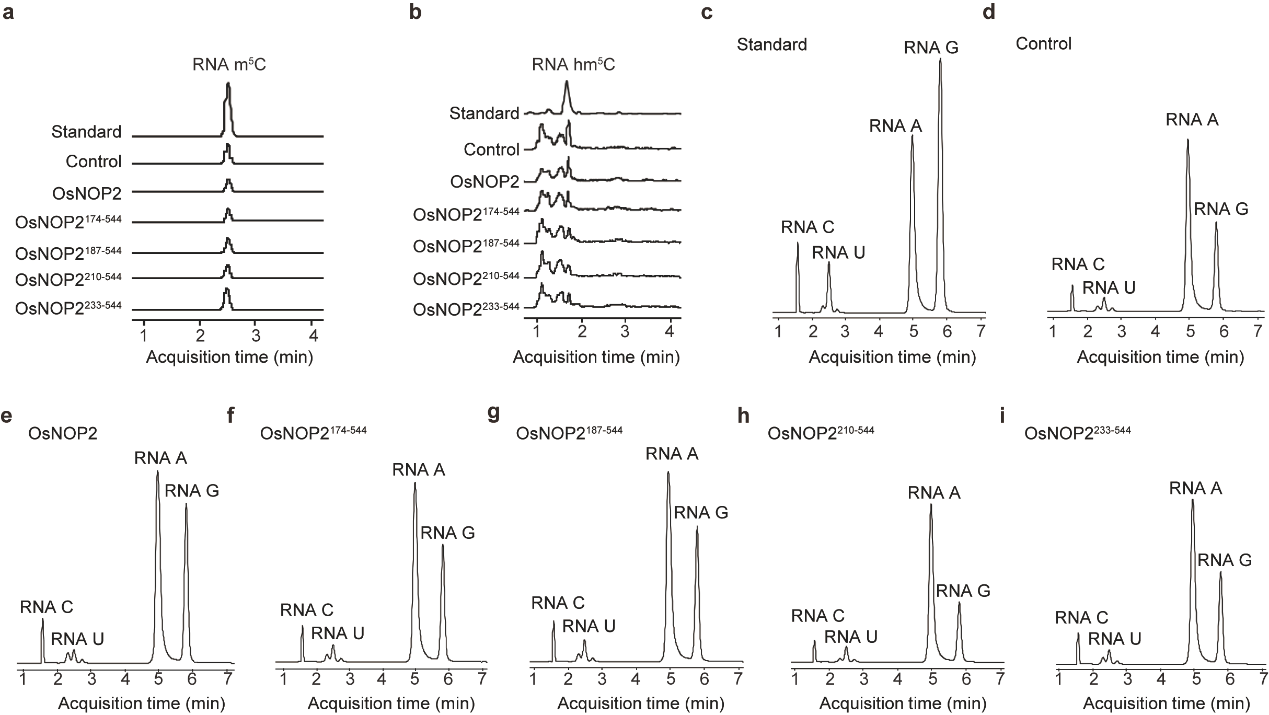


**Fig. S28 HPLC-MS/MS profiles of nucleoside standards and reaction products from *in vitro* demethylation assays. a**, **b** HPLC-MS/MS chromatograms of synthetic RNA oligonucleotides containing a single m^5^C modification used for *in vitro* demethylation assays. Peaks representing m^5^C (**a**) and hm^5^C (**b**) signals in reactions with the standard, control, wild-type OsNOP2, and indicated OsNOP2 truncation fragments. **c-i**, HPLC-MS/MS peak profiles of all major nucleosides, including m^5^C, hm^5^C, A, U, C, and G. Chromatograms for the nucleoside standards (**c**) and control reactions (**d**) for comparison with peaks detected in reactions containing wild-type OsNOP2 (e) or the respective OsNOP2 truncation fragments (**f–i**).


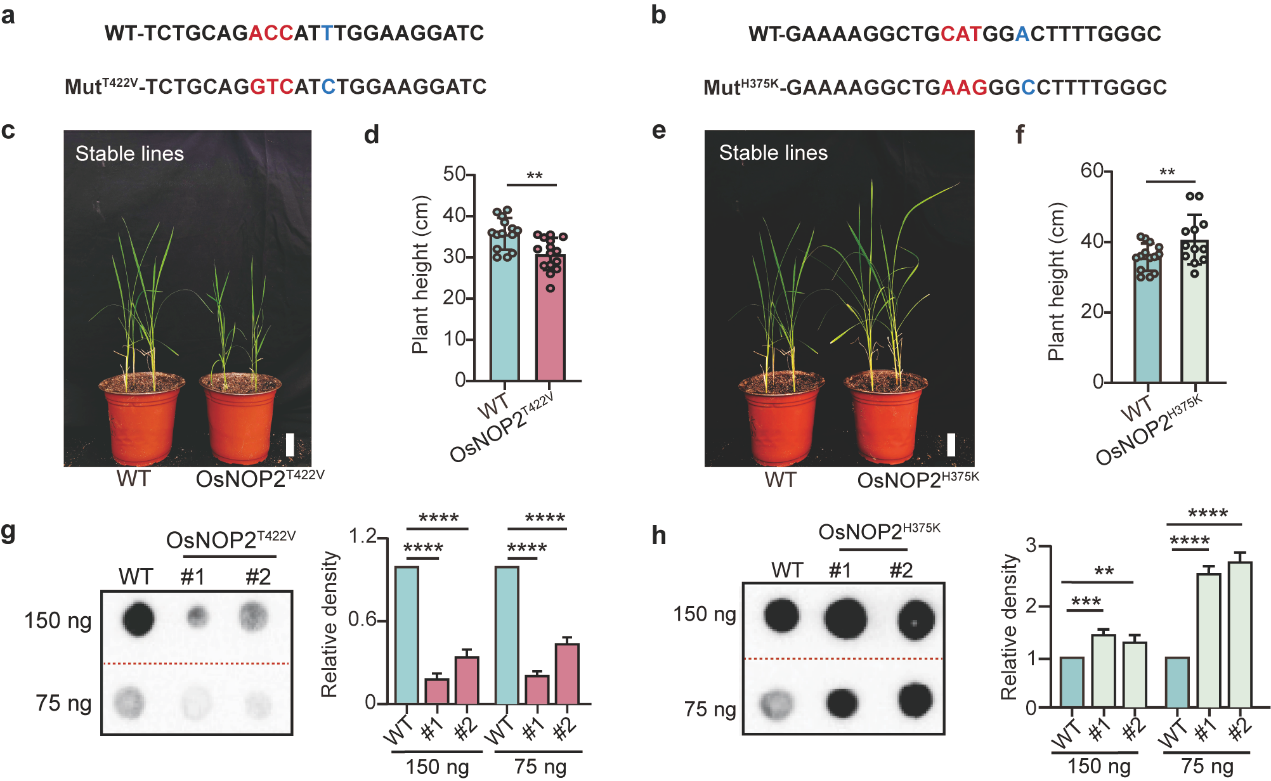


**Fig.** **S29 Creation of the stable transgenic lines with site-directed mutagenesis of amino acids. a**, **b,** The mutation sites and forms of the stable transgenic lines with site-directed mutagenesis of amino acids from OsNOP2^T422V^ (**a**) and OsNOP2^H375K^ (**b**). The red letters indicate the target mutation sites and the blue letters indicate the synonymous mutation sites. **c, e**, Phenotypes of the stable transgenic lines with site-directed mutagenesis of amino acids from OsNOP2^T422V^ (**c**) and OsNOP2^H375K^ (**e**). Scale bars, 5 cm. **d**, **f**, Statistical analysis of the plant height from stable transgenic lines (OsNOP2^T422V^) (**d**) and (OsNOP2^H375K^) (**f**) of T1 grown in the greenhouse. **g-h**, Detection of total RNA m^5^C level of 3-week-old stable transgenic lines (OsNOP2^T422V^) (**g**) and (OsNOP2^H375K^) (**h**), and the quantifications were displayed on the right of the figures, respectively. Data are shown as mean ± S.D. (**d**, n = 15 plants; **f**, n ≥ 12 plants; **g-h**, n = 3 biological replicates;). ns, no significance, **P* < 0.05, ***P* < 0.01, ****P* < 0.001, *****P* < 0.0001; In **d**, and **f**, *P* values are from two-tailed Student’s t-tests. In **g-h**, *P* values are from one-way ANOVA (and nonparametric or mixed).


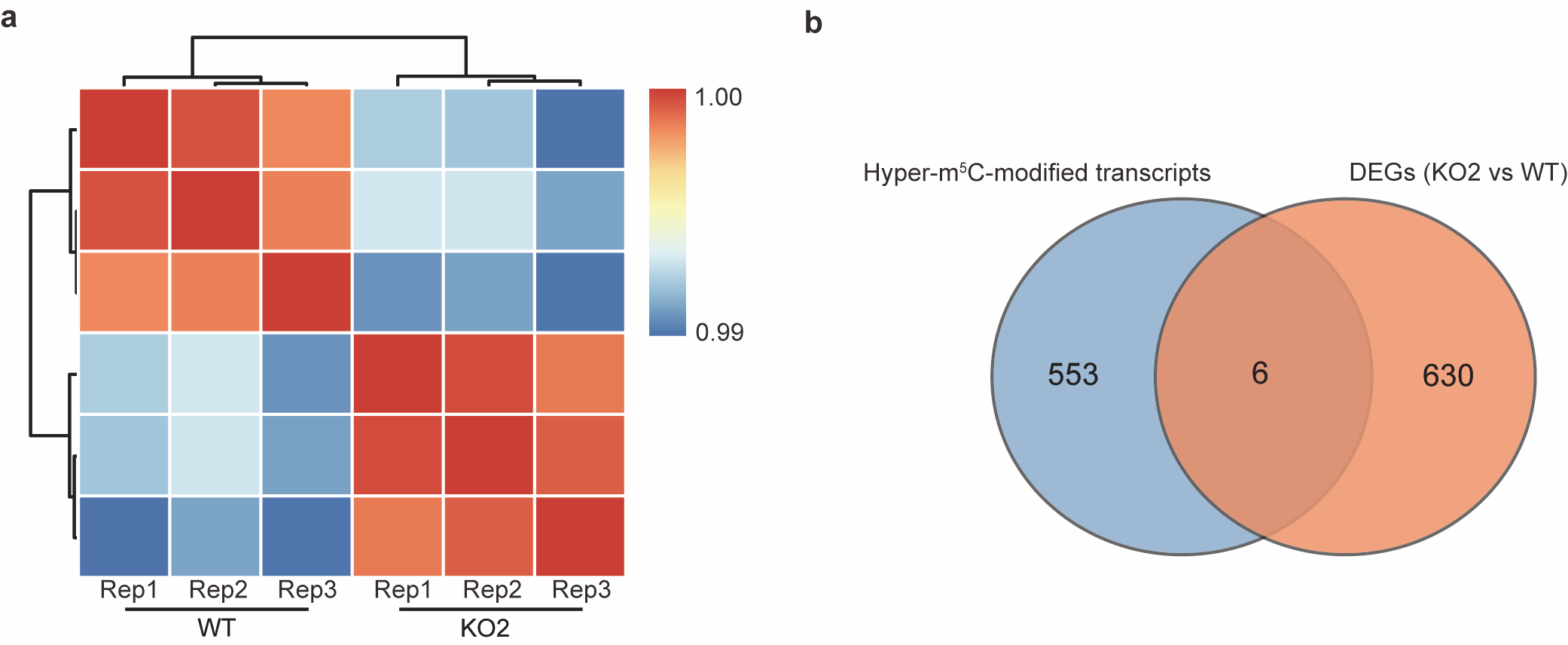


**Fig. S30 The OsNOP2-dependent RNA m^5^C modification nearly affects the transcription level of target transcripts. a**, The heatmap shows the Pearson correlation coefficient between the biological replicates. **b**, Overlap between hyper-m^5^C-modified transcripts with differentially expressed genes (DEGs) in KO2 versus WT plants.


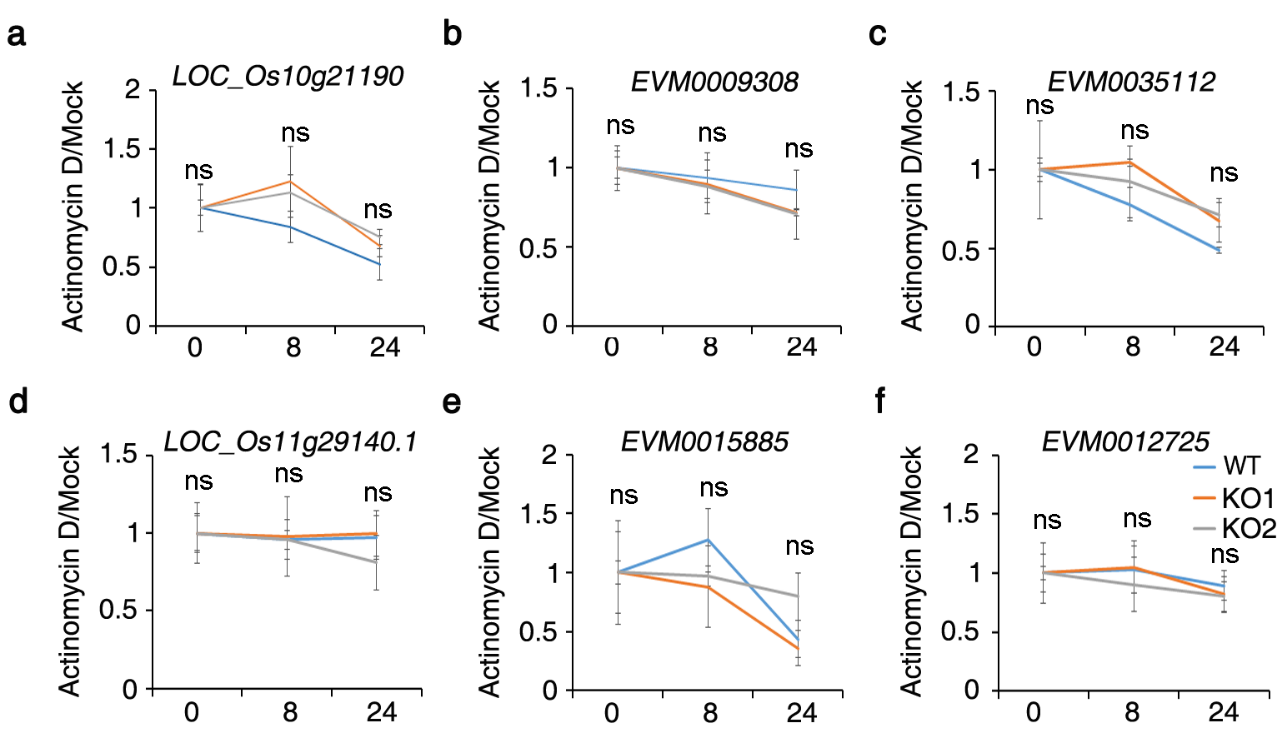


**Fig. S31 mRNA stability of** **RNA m^5^C-modified genes. a-f**, RT-qPCR analysis of transcript decay rates among six RNA m^5^C-modified genes in shoot tissues of 7-day-old *OsNOP2*-KO1, -KO2, and wild-type (WT) plants treated for 24 h with actinomycin D or the vehicle control. Relative expression was calculated by normalizing gene expression in actinomycin D-treated samples against that in DMSO-treated vehicle controls. Data represent means ± S.D. of n = 3 biological replicates. ns, no significance; *P* values were determined by two-tailed paired Student’ t-test.


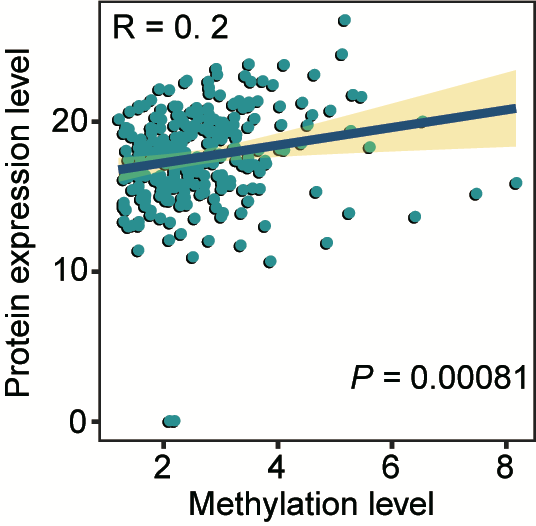


**Fig. S32 Pearson correlation coefficient of the proteomics between the biological replicates in WT and KO2 plants.**


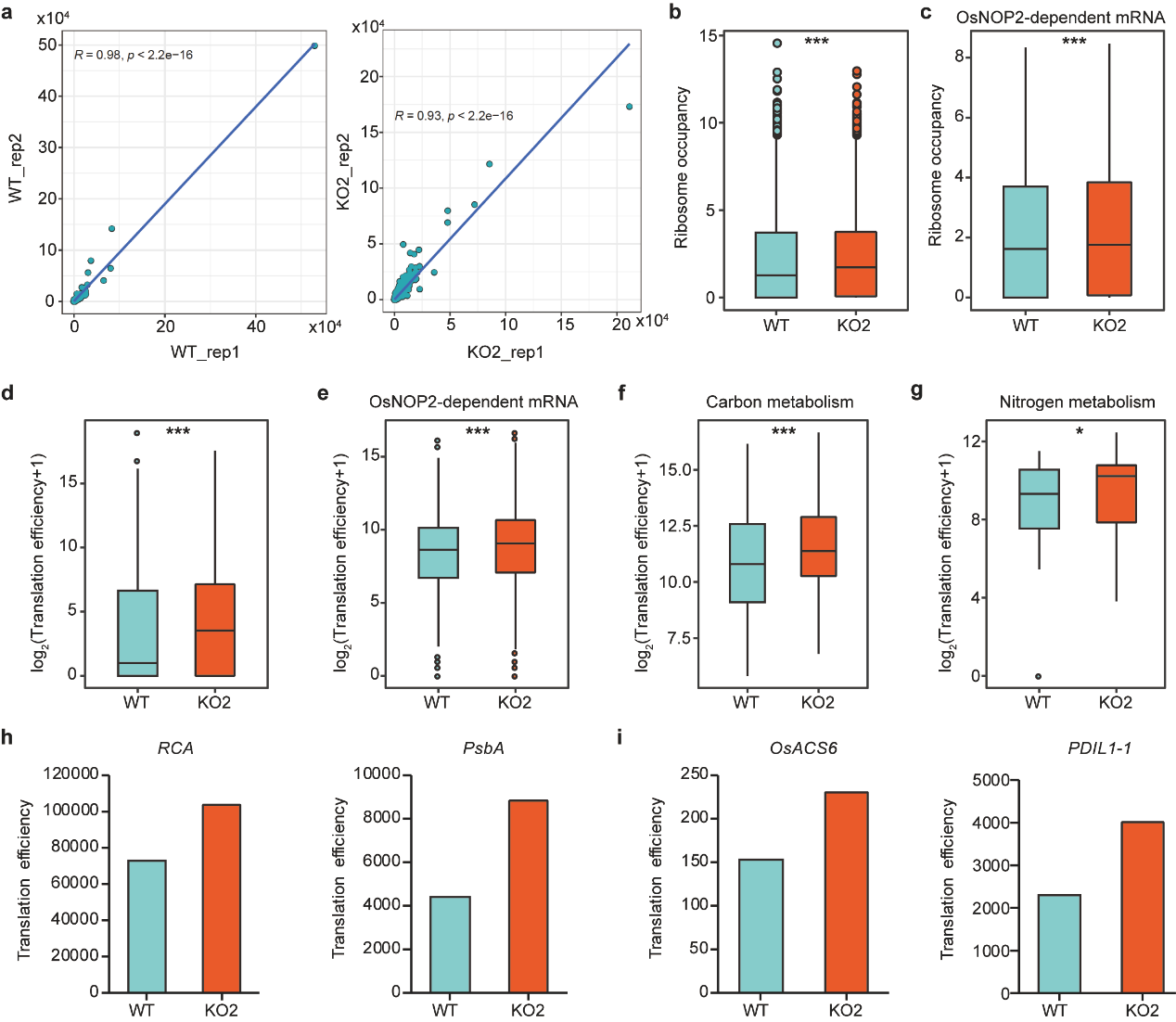


**Fig. S33 Changes in translation efficiency between WT and *OsNOP2*-KO2 plants. a**, Scatter plots of pairwise correlations for the same OsNOP2 target transcripts between replicates in WT (left) and *OsNOP2*-KO2 (right) plants. **b**, Ribo-seq analysis of global ribosome occupancy between KO2 and WT plants. **c**, Comparison of ribosome occupancy by OsNOP2 target genes between KO2 and WT plants. **d**, Comparison of global translation efficiency between KO2 and WT plants. **e**, Comparison of translation efficiency for OsNOP2-target genes between KO2 and WT plants. **f**, Comparison of translation efficiency among the carbon metabolism-related subset in KO2 and WT plants. **g**, Comparison of translation efficiency for nitrogen metabolism-related genes between KO2 and WT plants. **h-i**, Translation efficiencies of *RCA*, *PsbA* (h), *OsACS6*, and *PDIL1* (i) between KO2 and WT plants. Data are means ± S.D. (n = 2 biological replicates). **P* < 0.05, ****P* < 0.001; *P* values are from two-tailed Student’s t-tests.


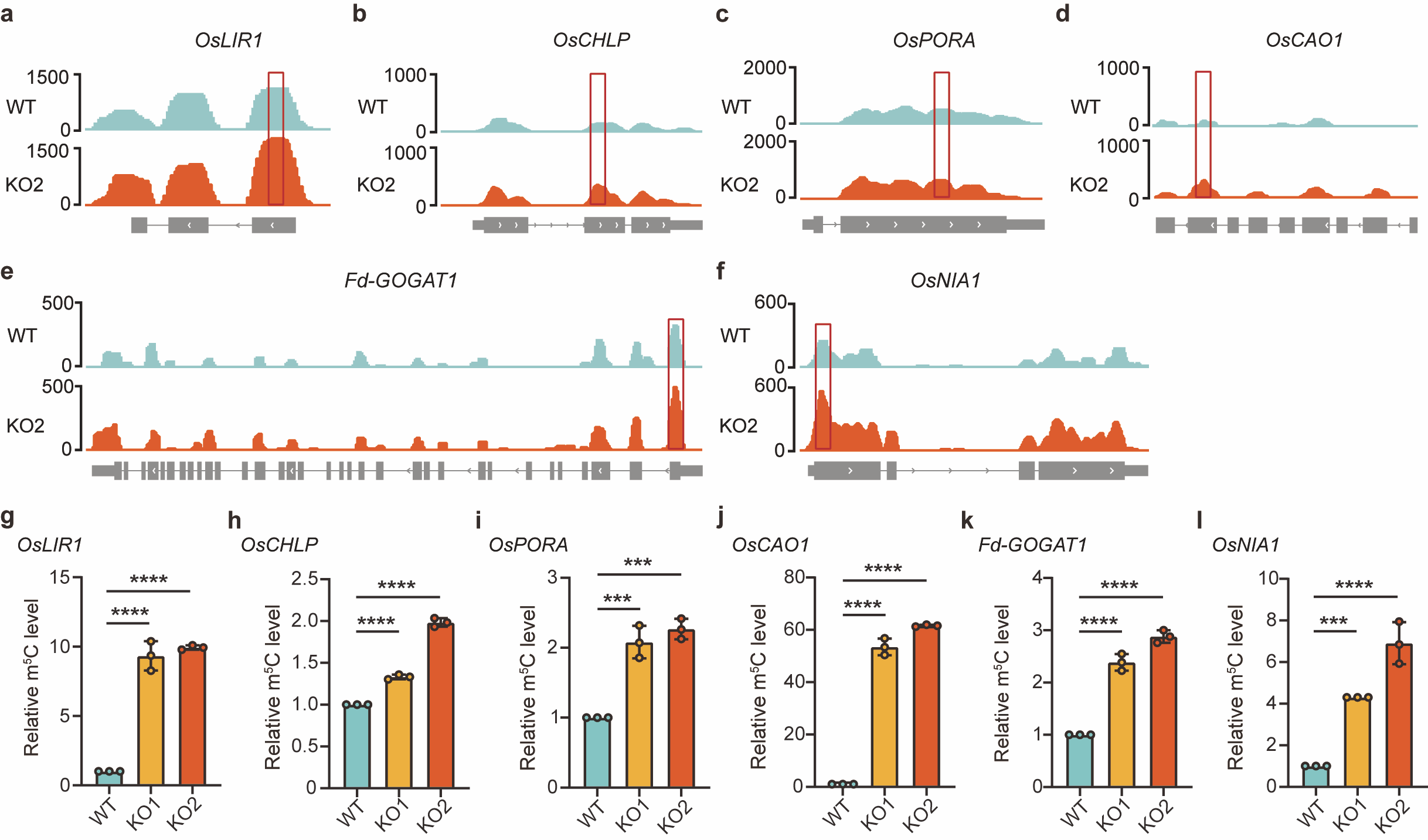


**Fig. S34 Validation of target transcripts related to carbon metabolism and nitrogen metabolism by MeRIP-qPCR assay. a-f**, Browser representation of the m^5^C reads of target transcripts related to carbon metabolism (*OsLIR1*, *OsCHLP*, *OsPORA*, and *OsCAO1*), and related to nitrogen metabolism (*Fd-GOGAT1* and *OsNIA1*) detected by MeRIP-seq displaying by integrative genomics viewers (IGV) tracks. The red box represents the experimental verification region. **g-l**, The relative RNA m^5^C level of *OsLIR1*, *OsCHLP*, *OsPORA*, *OsCAO1*, *Fd-GOGAT1*, and *OsNIA1* detected by MeRIP-qPCR. Data are mean ± S.D. (**g-l**, n = 3 biological replicates). ****P* < 0.001, *****P* < 0.0001; *P* values are from one-way ANOVA (and nonparametric or mixed).


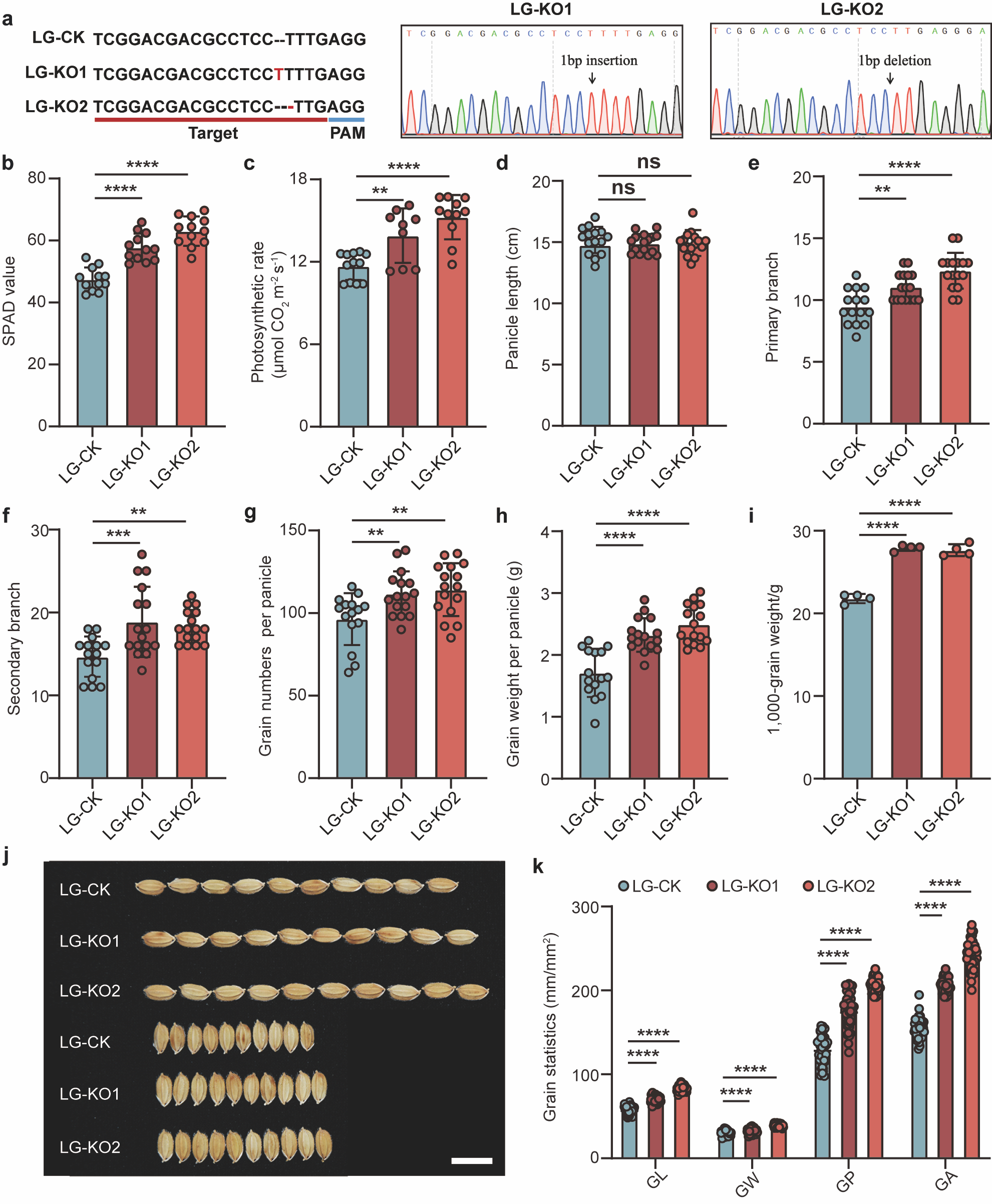


**Fig. S35 *OsNOP2* knockout improves the parameters of agronomic traits in variety Longgeng31.** **a**, The mutation sites and forms of LG-KO1 and LG-KO2. PAM, protospacer adjacent motif. **b-c**, Statistical analysis of the SPAD value (**b**) and photosynthetic rate (**c**) from Longgeng31 (LG-CK), LG-KO1, and LG-KO2 plants grown in the field in Langfang. **d-i**, Statistical analysis of the panicle length (**d**), primary branch (**e**), secondary branch (**f**), grain numbers per panicle (**g**), grain weight per panicle (**h**), and 1,000-grain weight (**i**) from Longgeng31 (LG-CK), LG-KO1, and LG-KO2 plants grown in the field in Langfang. **j**, Grain morphologies of LG-CK, LG-KO1, and LG-KO2 grains. Scale bar, 1 cm. **k**, Statistical analysis of grain length (GL), grain width (GW), grain perimeter length (GP), and grain area size (GA) of LG-CK, LG-KO1, and LG-KO2 plants. Data are mean ± S.D. (**b-c**, n ≥ 10 plants; **d-h**, n = 15 panicles; **i**, n = 4 biological replicates; **k**, n > 30 grains). ns, no significance, ***P* < 0.01, ****P* < 0.001, *****P* < 0.0001; In **b-i**, *P* values are from one-way ANOVA (and nonparametric or mixed). In **k**, *P* values are from two-way ANOVA (and nonparametric or mixed).


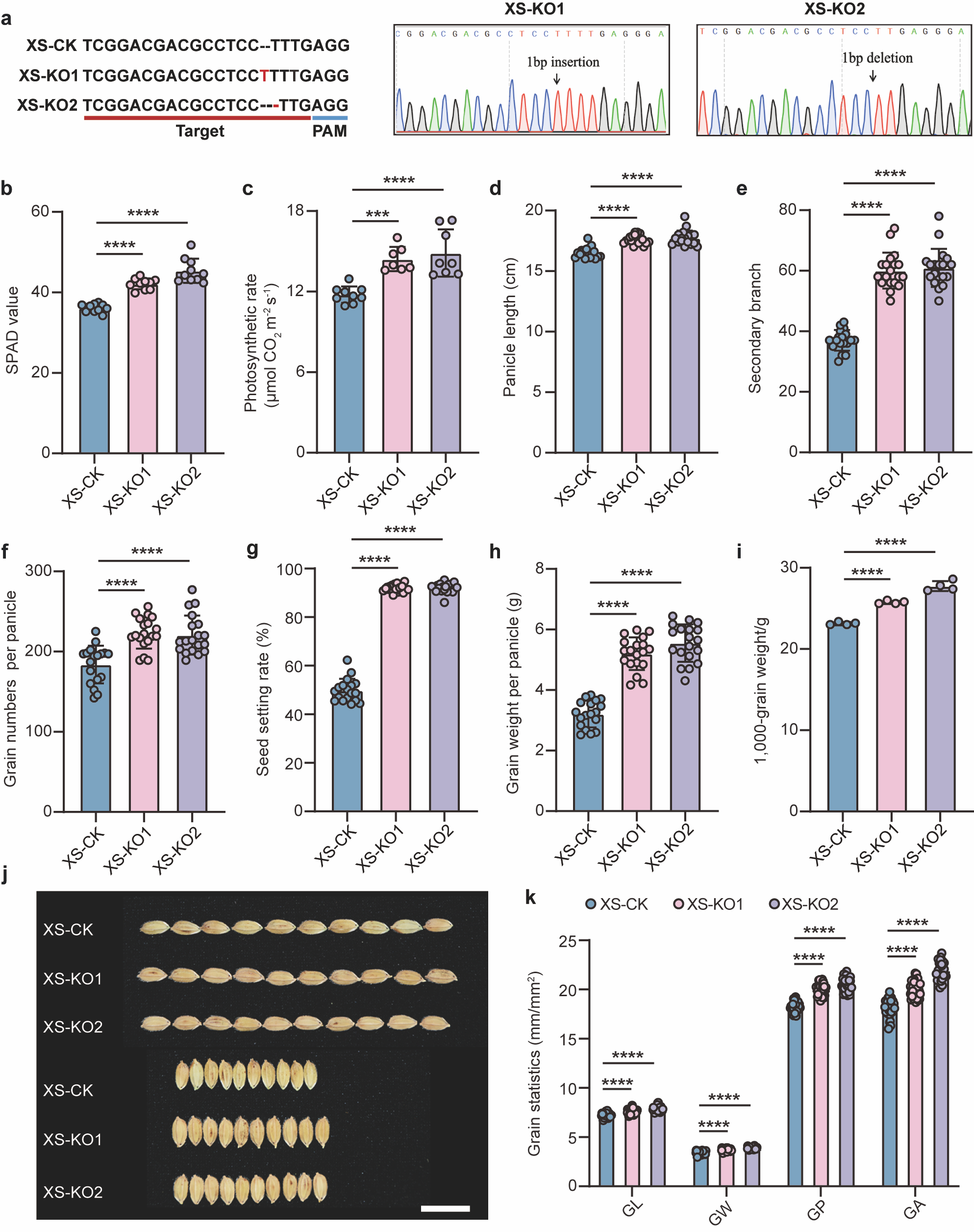


**Fig. S36 *OsNOP2* knockout enhances the parameters of agronomic traits in variety Xiushui134.** **a**, The mutation sites and forms of XS-KO1 and XS-KO2. PAM, protospacer adjacent motif. **b-c**, Statistical analysis of the SPAD value (**b**) and photosynthetic rate (**c**) from Xiushui134 (XS-CK), XS-KO1, and XS-KO2 plants grown in the field in Langfang. **d-i**, Statistical analysis of the panicle length (**d**), secondary branch (**e**), grain numbers per panicle (**f**), seed setting rate (**g**), grain weight per panicle (**h**), and 1,000-grain weight (**i**) from Xiushui134 (XS-CK), XS-KO1, and XS-KO2 plants grown in the field in Langfang. **j**, Grain morphologies of XS-CK, XS-KO1, and XS-KO2 grains. Scale bar, 1 cm. **k**, Statistical analysis of grain length (GL), grain width (GW), grain perimeter length (GP), and grain area size (GA) of XS-CK, XS-KO1, and XS-KO2 plants. Data are mean ± S.D. (**b-c**, n ≥ 7 plants; **d-h**, n = 20 panicles; **i**, n = 4 biological replicates; **k**, n > 30 grains). ****P* < 0.001, *****P* < 0.0001; In **b-i**, *P* values are from one-way ANOVA (and nonparametric or mixed). In **k**, *P* values are from two-way ANOVA (and nonparametric or mixed).


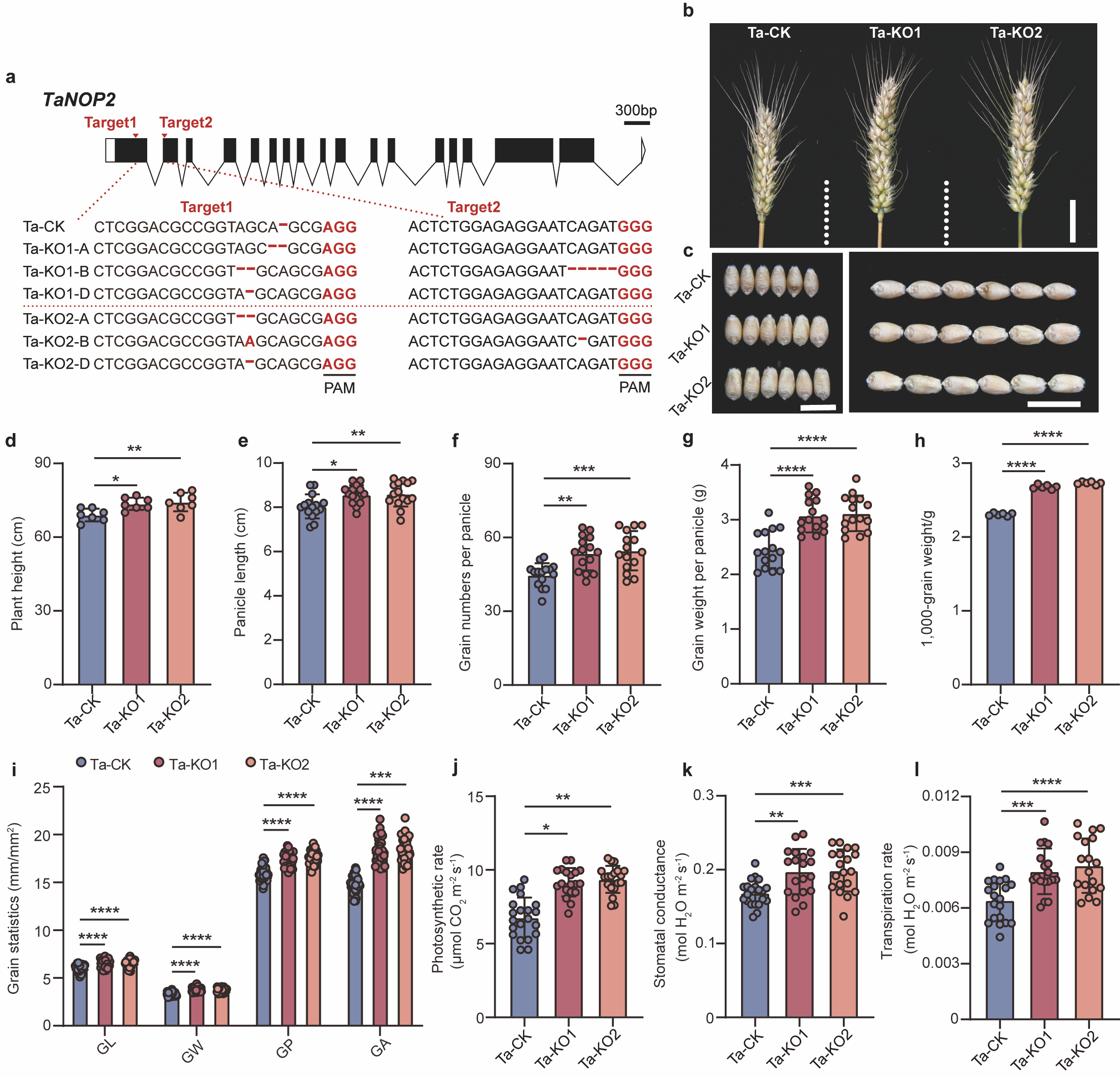


**Fig. S37 *Ta-NOP2* knockout significantly promotes the yield traits and photosynthetic parameters of wheat. a**, The gene structure and mutation sites of Ta-KO1 and Ta-KO2, and the PAM motifs were highlighted in red. PAM, protospacer adjacent motif. **b-c**, Phenotype of panicle (**b**) and grain morphologies (**c**) of Ta-CK, Ta-KO1, and Ta-KO2 plants grown in the field in Beijing. Scale bars, 3 cm (**b**) and 1 cm (**c**). **d-i**, Statistical analysis of the plant height (**d**), panicle length (**e**), grain numbers per panicle (**f**), grain weight per panicle (**g**), 1,000-grain weight (**h**), and grain length (GL), grain width (GW), grain perimeter length (GP), and grain area size (GA) (**i**) of Ta-CK, Ta-KO1, and Ta-KO2 plants. **j-l**, Statistical analysis of the photosynthetic rate (**j**), stomatal conductance (**k**), and transpiration rate (**l**) of Ta-CK, Ta-KO1, and Ta-KO2 plants. Data are mean ± S.D. (**d**, n = 7 plants; **e-g**, n = 15 panicles; **h**, n = 6 biological replicates; **i**, n ≥ 20 grains; **j-l**, n ≥ 15 plants). **P* < 0.05, ***P* < 0.01, ****P* < 0.001, *****P* < 0.0001; *P* values are from one-way ANOVA (and nonparametric or mixed).


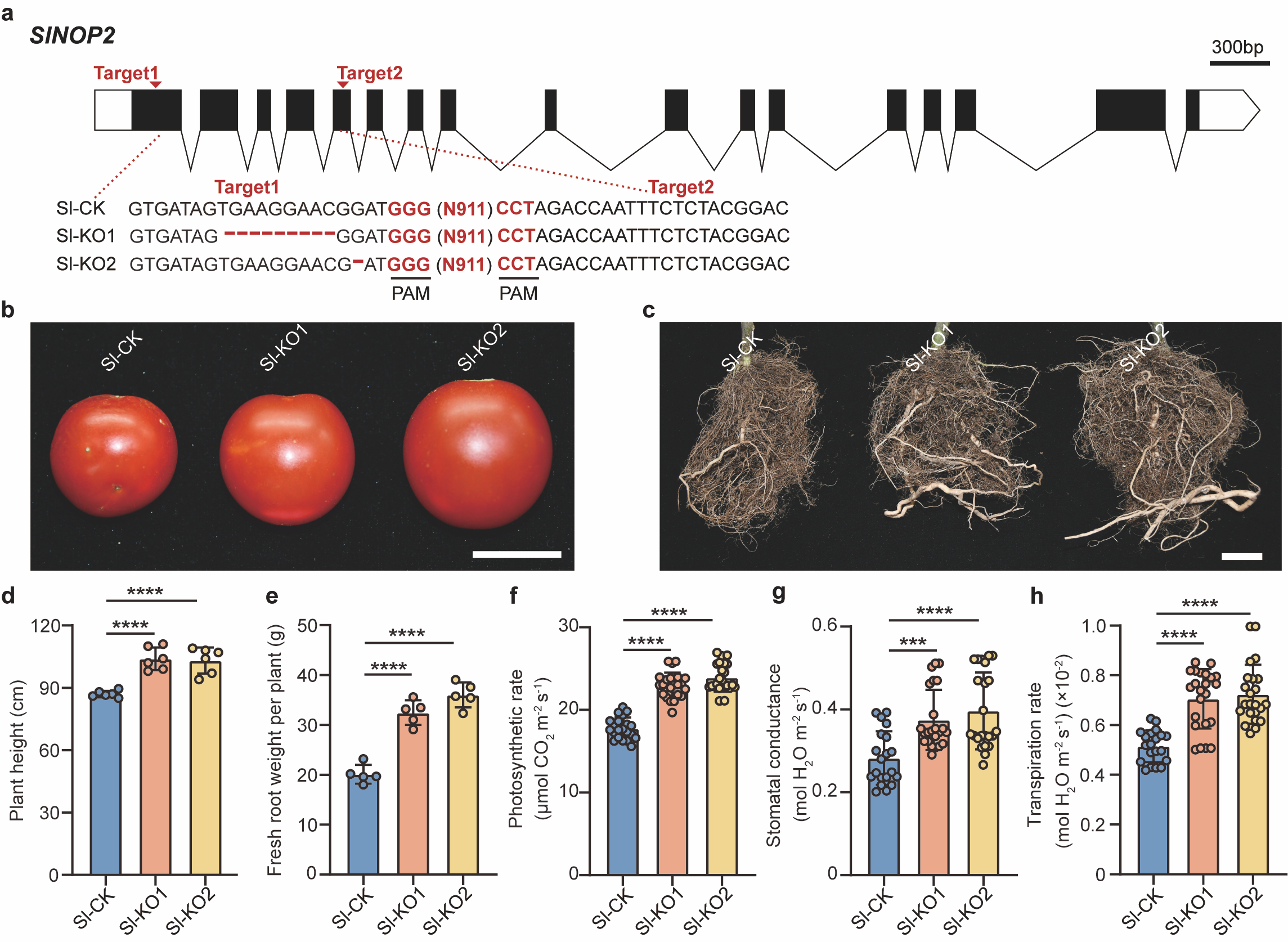


**Fig. S38 *Sl-NOP2* knockout significantly improves the yield traits and photosynthetic parameters of tomato. a**, The gene structure and mutation sites of Sl-KO1 and Sl-KO2, and the PAM motifs were highlighted in red. PAM, protospacer adjacent motif. **b-c**, Phenotype of the individual fruit (**b**) and root (**c**) of Sl-CK, Sl-KO1, and Sl-KO2 plants. Scale bars, 3 cm (**b-c**). **d-e**, Statistical analysis of the plant height (**d**) and fresh root weight per plant (**e**) of Sl-CK, Sl-KO1, and Sl-KO2 plants grown in the greenhouse at the mature stage. **f-h**, Statistical analysis of the photosynthetic rate (**f**), stomatal conductance (**g**), and transpiration rate (**h**) of Sl-CK, Sl-KO1, and Sl-KO2 plants grown in the greenhouse at the anthesis stage. Data are mean ± S.D. (**d**, n = 6 plants; **e**, n = 5 plants; **f-h**, n ≥ 20 plants). ****P* < 0.001, *****P* < 0.0001; *P* values are from one-way ANOVA (and nonparametric or mixed).

**Tables S2-14**

**Table S2 Agronomic traits of rice plants grown in the field in Langfang in 2020.**

|  | WT | KO1 | KO2 | n^a^ |
| --- | --- | --- | --- | --- |
| Plant height (cm) | 106.2±2.81 | 110.5±3.89 **** | 114.9±2.99 **** | 15 |
| Number of effective tillers per plant | 23.4±1.22 | 26.43±1.34 **** | 26.93±1.44 **** | 15 |
| Panicle length (cm) | 20.43±1.42 | 21.79±0.59 ** | 21.93±0.89 *** | 15 |
| Grain number per panicle | 141.4±18.15 | 159.08±20.01 * | 163.07±14.69 ** | 15 |
| Primary branch | 11.4±0.74 | 12.5±0.8 *** | 12.87±0.64 **** | 15 |
| Secondary branch | 26.47±3.56 | 30.42±3.26 ** | 32.07±2.79 **** | 15 |
| 1,000-grain weight (g) | 22.1±0.56 | 24.03±0.65 *** | 25.61±0.5 **** | 1,000^4^ |
| Grain length (mm) | 7.56±0.26 | 8.01±0.38 **** | 7.91±0.27 **** | > 30 |
| Grain width (mm) | 3.28±0.15 | 3.6±0.13 **** | 3.54±0.11 **** | > 30 |
| Grain perimeter length (mm) | 18.44±0.76 | 19.88±0.81 **** | 19.73±0.84 **** | > 30 |
| Grain area size (mm^2^) | 18.37±0.99 | 20.74±1.12 **** | 20.52±0.94 **** | > 30 |
| Plant height (cm) | 106.2±2.81 | 110.5±3.89 **** | 114.9±2.99 **** | 15 |

a: Number of biological replicates.

Agronomic traits of WT, KO1, and KO2 rice were obtained from field-grown plants in Langfang (grown from May to October, 2020). Data are presented as means ± S.D. **P* < 0.05, ***P* < 0.01, ****P* < 0.001, *****P* < 0.0001; *P* values are from one-way ANOVA (and nonparametric or mixed). Compared with WT.

**Table S3 Agronomic traits of rice plants grown in the field in Langfang in 2021.**

|  | WT | KO1 | KO2 | n^a^ |
| --- | --- | --- | --- | --- |
| Plant height (cm) | 112.7±2.81 | 120.2±2.2 **** | 124±1.89 **** | 30 |
| Number of effective tillers per plant | 20.7±3.74 | 28.6±3.75 *** | 30.1± 4.12 **** | 30 |
| Panicle length (cm) | 20.43±0.87 | 21.49±1.03 ** | 22.9±1.55 **** | 60 |
| Grain number per panicle | 146.43±23.97 | 162.28±22.06 ** | 181.18±26.22 **** | 60 |
| Primary branch | 11.4±0.97 | 12.14±1.19 * | 13.23±1.15 **** | 60 |
| Secondary branch | 27.07±5.19 | 31.83±4.6 *** | 33.68±5.35 **** | 60 |
| 1,000-grain weight (g) | 23.1±0.74 | 26.55±0.81 *** | 27.75±0.3 **** | 1,000^4^ |
| Grain length (mm) | 7.26±0.3 | 8.08±0.24 **** | 8.63±0.26 **** | > 30 |
| Grain width (mm) | 3.14±0.17 | 3.7±0.12 **** | 3.89±0.17 **** | > 30 |
| Grain perimeter length (mm) | 17.02±0.78 | 20.5±0.61 **** | 21.63±0.7 **** | > 30 |
| Grain area size (mm^2^) | 18.42±1.19 | 21.75±0.79 **** | 24.39±1.38 **** | > 30 |
| Seed setting rate (%) | 77.73±7.36 | 75.51±7.46 ^ns^ | 87.42±3.33 ** | 60 |
| Biomass (g) | 149.73±17.57 | 185.8±18.94 * | 191.74±37.04 ** | 30 |
| Grain yield per plant (g) | 59.49±10.2 | 74.93±10.41 * | 78.14±17.54 ** | 30 |
| Harvest index | 0.4±0.03 | 0.4±0.04 ^ns^ | 0.41±0.02 ^ns^ | 30 |

a: Number of biological replicates.

Agronomic traits of WT, KO1, and KO2 rice were obtained from field-grown plants in Langfang (grown from May to October, 2021). Data are presented as means ± S.D. ns, no significance, **P* < 0.05, ***P* < 0.01, ****P* < 0.001, *****P* < 0.0001; *P* values are from one-way ANOVA (and nonparametric or mixed). Compared with WT.

**Table S4 Agronomic traits of rice plants grown in the field in Hainan in 2021.**

|  | WT | KO1 | KO2 | n^a^ |
| --- | --- | --- | --- | --- |
| Plant height (cm) | 72.7±4.81 | 76.2±3.28 ** | 78.3±2.98 ** | 15 |
| Number of effective tillers per plant | 16.8±3.34 | 25.86±4.62 **** | 23.2± 3.89 *** | 15 |
| 1,000-grain weight (g) | 24.1±0.48 | 27.15±0.34 **** | 27.85±0.25 **** | 1,000^4^ |
| Grain length (mm) | 8.04±0.19 | 8.51±0.29 **** | 8.57±0.3 **** | > 30 |
| Grain width (mm) | 3.44±0.13 | 3.7±0.09 **** | 3.84±0.12 **** | > 30 |
| Grain perimeter length (mm) | 19.86±0.97 | 21.29±1.06 **** | 21.67±0.69 **** | > 30 |
| Grain area size (mm^2^) | 20.53±0.89 | 22.34±1.2 **** | 24.53±1.08 **** | > 30 |

a: Number of biological replicates.

Agronomic traits of WT, KO1, and KO2 rice were obtained from field-grown plants in Hainan (grown from December 2021 to April 2022). Data are presented as means ± S.D. ***P* < 0.01, ****P* < 0.001, *****P* < 0.0001; *P* values are from one-way ANOVA (and nonparametric or mixed). Compared with WT.

**Table S5 Agronomic traits of rice plants grown in the field in Langfang in 2022.**

|  | WT | KO1 | KO2 | n^a^ |
| --- | --- | --- | --- | --- |
| Plant height (cm) | 103.8±5.9 | 110.4±5.2 **** | 113.2±3.9 **** | 50 |
| Number of effective tillers per plant | 13.24±2.04 | 16.14±2.25 **** | 15.96±1.89 **** | 50 |
| Panicle length (cm) | 20.36±1.18 | 22.06±1.03 **** | 22.03±1.12 **** | 30 |
| Grain number per panicle | 133.7±15.6 | 162±16.22 **** | 158.87±13.66 **** | 30 |
| Primary branch | 10.6±0.89 | 11.27±1.05 **** | 12.23±0.86 **** | 30 |
| Secondary branch | 23.7±3.77 | 30.93±3.6 **** | 28.33±3.49 **** | 30 |
| Grain weight per panicle (g) | 3.19±0.41 | 3.56±0.35 **** | 3.61±0.3 **** | 30 |
| 1,000-grain weight (g) | 24.13±0.85 | 25.5±0.05 **** | 26.58±0.3 **** | 1,000^4^ |
| Grain length (mm) | 7.82±0.28 | 8.02±0.23 **** | 8.58±0.34 **** | > 30 |
| Grain width (mm) | 3.46±0.14 | 3.61±0.09 * | 3.89±0.097 * | > 30 |
| Grain perimeter length (mm) | 19.34±0.71 | 20.18±0.57 **** | 21.64±0.704 **** | > 30 |
| Grain area size (mm^2^) | 19.44±0.96 | 20.96±0.74 **** | 23.9±0.75 **** | > 30 |
| Seed setting rate (%) | 90.47±7.63 | 87.59±3.2 ^ns^ | 86.47±4.7 ^ns^ | 30 |
| Biomass (g) | 113.04±15.63 | 164.52±39.67 **** | 167.68±36.41 **** | 50 |
| Grain yield per plant (g) | 45.24±7.16 | 62.16±14.82 **** | 59.42±14.38 **** | 50 |
| Grain yield per plot (kg) | 4.31±0.5 | 5.5±0.43 * | 5.54±0.53 * | 150^3^ |

a: Number of biological replicates.

Agronomic traits of WT, KO1, and KO2 rice were obtained from field-grown plants in Langfang (grown from May to October, 2022). Data are presented as means ± S.D. ns, no significance, **P* < 0.05, ***P* < 0.01, ****P* < 0.001, *****P* < 0.0001; *P* values are from one-way ANOVA (and nonparametric or mixed). Compared with WT.

**Table S6 Agronomic traits of rice plants grown in the field in Hainan in 2022.**

|  | WT | KO1 | KO2 | n^a^ |
| --- | --- | --- | --- | --- |
| Plant height (cm) | 73.36±2.78 | 77.93±2.57 **** | 79.9±2.68 **** | 20 |
| Number of effective tillers per plant | 23.21±4.27 | 25±4.53 **** | 27.9±3.21 **** | 20 |
| Panicle length (cm) | 16.71±1.56 | 18.37±1.72 ** | 18.31±0.89 ** | 20 |
| Grain number per panicle | 67±13.54 | 101.3±30.12 **** | 95.8±9.26 *** | 20 |
| Primary branch | 5.4±0.97 | 7±1.15 **** | 7.3±0.95 **** | 20 |
| Secondary branch | 13.3±2.75 | 18.4±4.35 **** | 18.3±2.95 **** | 20 |
| Grain weight per panicle (g) | 1.35±0.19 | 1.62±0.18 *** | 1.57±0.32 ** | 20 |
| 1,000-grain weight (g) | 25.06±0.18 | 27.28±0.13 **** | 28.49±0.15 **** | 1,000^4^ |
| Grain length (mm) | 8.04±0.3 | 8.49±0.18 **** | 8.74±0.36 **** | > 30 |
| Grain width (mm) | 3.61±0.16 | 3.81±0.09 **** | 3.89±0.15 **** | > 30 |
| Grain perimeter length (mm) | 20.16±1.05 | 21.57±1.1 **** | 22.22±0.86 **** | > 30 |
| Grain area size (mm^2^) | 20.73±0.91 | 22.71±1.02 ** | 25.18±1.27 **** | > 30 |
| Seed setting rate (%) | 72.53±5.9 | 71.86±8.9 ^ns^ | 72.02±2.58 ^ns^ | 20 |
| Grain yield per plant (g) | 20.25±2.51 | 28.22±4.48 **** | 30.14±4.52 **** | 20 |

a: Number of biological replicates.

Agronomic traits of WT, KO1, and KO2 rice were obtained from field-grown plants in Hainan (grown from December 2022 to April 2023). Data are presented as means ± S.D. ns, no significance, ***P* < 0.01, ****P* < 0.001, *****P* < 0.0001; *P* values are from one-way ANOVA (and nonparametric or mixed). Compared with WT.

**Table S7 Agronomic traits of rice plants grown in the field in Langfang in 2023.**

|  | WT | KO1 | KO2 | n^a^ |
| --- | --- | --- | --- | --- |
| Plant height (cm) | 107.8±3.51 | 119.03±2.91 **** | 123.13±3.66 **** | 60 |
| Number of effective tillers per plant | 15.8±2.93 | 21.6±4.72 **** | 21.3±3.46 **** | 60 |
| Panicle length (cm) | 19.63±1.03 | 21.26±0.9 **** | 22.16±1.02 **** | 30 |
| Grain number per panicle | 120.4±9.1 | 147.81±26.65 **** | 150.29±14.15 **** | 30 |
| Primary branch | 10.87±0.92 | 12.25±1.18 **** | 12.71±1.05 **** | 30 |
| Secondary branch | 22.47±3.8 | 26±3.39 **** | 26.82±4.45 **** | 30 |
| Grain weight per panicle (g) | 2.88±0.32 | 3.43±0.34 **** | 3.52±0.25 **** | 30 |
| 1,000-grain weight (g) | 25±0.43 | 26.45±0.25 **** | 26.8±0.16 **** | 1,000^4^ |
| Grain length (mm) | 7.88±0.37 | 8.41±0.25 **** | 8.32±0.31 **** | > 30 |
| Grain width (mm) | 3.54±0.15 | 3.88±0.17 **** | 3.84±0.18 **** | > 30 |
| Grain perimeter length (mm) | 19.67±0.8 | 21.42±0.7 **** | 21.13±0.77 **** | > 30 |
| Grain area size (mm^2^) | 20.27±1.19 | 24.09±1.2 **** | 23.8±1.1 **** | > 30 |
| Seed setting rate (%) | 93.51±2.19 | 94.63±2.4 ^ns^ | 94.5±2.7 ^ns^ | 30 |
| Biomass (g) | 101.06±13.27 | 154.32±15.56 **** | 162.03±16.23 **** | 50 |
| Grain yield per plant (g) | 32±5.92 | 44.43±8.19 **** | 46.43±6.15 **** | 50 |
| Grain yield per plot (kg) | 8.69±0.23 | 10.46±1.46 * | 10.07±0.12 * | 232^3^ |

a: Number of biological replicates.

Agronomic traits of WT, KO1, and KO2 rice were obtained from field-grown plants in Langfang (grown from May to October, 2023). Data are presented as means ± S.D. ns, no significance, **P* < 0.05, *****P* < 0.0001; *P* values are from one-way ANOVA (and nonparametric or mixed). Compared with WT.

**Table S8 Primers used for construction of transformation vectors and characterization of transgenic plants.**

| **Name** | **Primer (5'-3')** | **Usage** |
| --- | --- | --- |
| OsNOP2-KO-F | GCGGGGAAGAAGGGGAAG | Identification of *OsNOP2* KO mutants (*Nipponbare*, *LG31*, *XS134*) |
| OsNOP2-KO-R | TAGCTCGCGTGAATGCTACA |  |
| OsNOP2-OE-SmaI-F | GTAGAAGAGGTACCCGGGATGGCGAAGAAGGGCGCGCC | *OsNOP2* overexpression in *Nipponbare* |
| OsNOP2-OE-XbaI-R | GCAGGTCGACTCTAGATCATACTTGTTTACTCCTCGA |  |
| OsNOP2-COM-Kpn1-F1 | ACGAATTCGAGCTCGGTACCAGTAGCTGATAATTCCACATTC | Construction of transformation vector for creating complementary materials |
| OsNOP2-COM-R1 | GTCGTCGGAGGACGACTCGAGCAGCCTCTGCTTCTTG |  |
| OsNOP2-COM-F2 | CTCGAGTCGTCCTCCGACGACTCCGAGCTGGAGCAGC |  |
| OsNOP2-COM-Kpn1-R2 | TCTAGAGGATCCCCGGGTACCTCATACTTGTTTACTCCTCGA |  |
| Ta-F | CTCGGACGCCGGTAGCAGCGAGG | Identification of *OsNOP2* KO mutants in Wheat (KN199) |
| Ta-R | CCCATCTGATTCCTCTCCAGAGT |  |
| Sl-F | GTGATAGTGAAGGAACGGATGGG | Identification of *OsNOP2* KO mutants in Tomato (AC) |
| Sl-R | GTCCGTAGAGAAATTGGTCTAGG |  |

**Table S9 Primers used for vector construction.**

| **Name** | **Primer (5'-3')** | **Usage** |
| --- | --- | --- |
| pAN580-OsNOP2-F | TCCGGAGCTAGCTCTAGAATGGCGAAGAAGGGCGCGCC | Subcellular localization |
| pAN580-OsNOP2-R | CACCATGGATCCCCCGGGTCATACTTGTTTACTCCTCGA |  |
| OsNOP2-GST-F | GGTTCCGCGTGGATCCATGGCGAAGAAGGGCGCGCC | OsNOP2 expression |
| OsNOP2-GST-R | GTCGACCCGGGAATTCTACTTGTTTACTCCTCGACTTC |  |
| OsNOP2^mut^-GST-F1 | GGTTCCGCGTGGATCCATGGCGAAGAAGGGCGCGCC | OsNOP2^mut^ expression |
| OsNOP2^mut^-GST-R1 | CGCTGTCTCCTCCCTCAAGGAGGCGTCGTCCGAGTC |  |
| OsNOP2^mut^-GST-F2 | GACTCGGACGACGCCTCCTTGAGGGAGGAGACAGCG |  |
| OsNOP2^mut^-GST-R2 | GTCGACCCGGGAATTCAGTCATCAGACTCATCTGAG |  |
| OsNOP2^T422V^-GST-F | GGTTCCGCGTGGATCCATGGCGAAGAAGGGCGCGCC | Verify the enzyme activity of OsNOP2 designed by prediction |
| OsNOP2^T422V^-GST-R | GGATCCTTCCAAATGACCCCTGTGCCTGTGCAGGGT |  |
| OsNOP2^H375K^-GST-F | GGTTCCGCGTGGATCCATGGCGAAGAAGGGCGCGCC |  |
| OsNOP2^H375K^-GST-R | GCGATGTATGTTGCCCAAAAGTCCCTTCAGCCTTTTC |  |
| OsNOP2^A2G^-GST-F | GGTTCCGCGTGGATCCATGGGGAAGAAGGGCGCGCC |  |
| OsNOP2^A2G^-GST-R | CTCATTGAACTCATTTGCATAAATTATTCCAGTATTC |  |
| NOP2-GST-F | GGTTCCGCGTGGATCCATGGGGCGCAAGTTGGACCCTACG | NOP2 expression |
| NOP2-GST-R | GTCGACCCGGGAATTCAGATAGCAGCAGCTGGCTGTTGCCCCTG |  |

**Table S10 Fragments used for demethylase enzyme activity and EMSA.**

| **Name** | **Primer (5'-3')** | **Usage** |
| --- | --- | --- |
| Bio-m^5^C-RNA | /5Biosg/rGrGrCrGrArUrGrCrGrG/i5M-rC-TOM/rGrGrCrGrGrArGrGrCrCrG | Enzyme activity in vitro and EMSA |
| Bio-C-RNA | /5Biosg/rGrGrCrGrArUrGrCrGrGrCrGrGrCrGrGrArGrGrCrCrG |  |

**Table S11 Primers used for identification of** **the stable transgenic lines with site-directed mutagenesis of amino acids.**

| **Name** | **Primer (5'-3')** | **Usage** |
| --- | --- | --- |
| OsNOP2^T422V^-F | TTGAATTGGGTGCAGCATTT | Identification of the stable transgenic lines |
| OsNOP2^T422V^-R | GCACAGTCTCTGATGTCCTCAAT |  |
| OsNOP2^H375K^-F | TGTCATGCACAGGAATAATTTATGC |  |
| OsNOP2^H375K^-R | GATTAAAATGGACCAAAAATTATTG |  |

**Table S12 Primers used for qRT-PCR and RNA m^5^C-IP-qPCR.**

| **Name** | **Primer (5'-3')** |
| --- | --- |
| qOsUBQ-F | GCTCCGTGGCGGTATCAT |
| qOsUBQ-R | CGGCAGTTGACAGCCCTAG |
| qOsNOP2-F | GAGGAAGACGATGAGGATGG |
| qOsNOP2-R | CATCTGAGTCCACACCAGAG |
| RCA-P1-F1 | CGATCGTCATCTTGGGCTGCTCG |
| RCA-P1-R1 | CACGGGTGTGGAGAACATTGGC |
| RCA-P2-F2 | CGAAGACGAGCTCACACTGG |
| RCA-P2-R2 | GGCTTCTACATCGCCCCTGC |
| RCA-control-F | CCACAGGCCACACGCTGAAG |
| RCA-control-R | CACAGACTCCGATCCAGGTCC |
| PsbA-P1-F1 | CCATTATTCCTACTTCTGCGGCG |
| PsbA-P1-R1 | CAATTAGCTCATAAGGACCACC |
| PsbA-P2-F2 | CGGATTCAATTTCAACCAATCTG |
| PsbA-P2-R2 | GTTGTGAGCATTACGTTCGTGC |
| PsbA-control-F | GCCGAGTTGGTACGAATTCC |
| PsbA-control-R | CATATACCTATTTACATAAC |
| OsACS6-P1-F1 | GGCGGAGCGGCAACGGCGGC |
| OsACS6-P1-R1 | GCCCCCCGCGTCCCTGCACC |
| OsACS6-P2-F2 | CAGGTCTTACAGCGAGAAAGG |
| OsACS6-P2-R2 | GTTCGCTCAATGTCGTGAAGC |
| OsACS6-control-F | AACAGATATTGATTGATACAAAG |
| OsACS6-control-R | GCCACCAAACGATTCATACCAACA |
| PDIL1-P1-F1 | GGCTGGGGCCGACTCTGCCTTCTC |
| PDIL1-P1-R1 | CTGCGAACGATGTGCCAAGCGAG |
| PDIL1-P2-F2 | CGTAGAACTCGACGACCATGAAC |
| PDIL1-P2-R2 | GGCTTGGATCTCGCTGCTGCTC |
| PDIL1-control-F | CAGATGTTTGCGAAAATCAAC |
| PDIL1-control-R | GGTGGAGAGGAATCTCCGAGC |
| OsLIR1-peak-F | CGCGCAACGCAACACGGCGAGGAG |
| OsLIR1-peak-R | CTAGCAGTGTCGTTGGATTGTCGG |
| OsCHLP-peak-F | CGGGTCGTACGTGGTGCACTACAAC |
| OsCHLP-peak-R | CTCCTGGAACGCGATGGCGTACTCG |
| OsPORA-peak-F | GCATCGCGACGACGGGCTTGTTCC |
| OsPORA-peak-R | CGCCCACCACCTGCGCCAGCCGC |
| OsCAO1-peak-F | GGTGGAGATGCGTCGAACATTGC |
| OsCAO1-peak-R | CAAGGATACTGGGATCCATACCCG |
| Fd-GOGAT1-peak-F | CTGCGCGGGCTTCTCCGCGGCCTC |
| Fd-GOGAT1-peak-R | GCCACGCTCCCACGTGCGGCGG |
| OsNIA1-peak-F | GCGGCAGTTCGGCCACCTCGAGC |
| OsNIA1-peak-R | CGCGGCGGTGGCGAGACGAGCGG |
| Sl-RCA-peak-F | GAAGACAGAATTGGTGTTTGC |
| Sl-RCA-peak-R | CAATTCCAGTACTTCCAATC |
| Sl-PsbA-peak-F | CCCAATCTGGGAAGCGGCATC |
| Sl-PsbA-peak-R | GATTCCTAGAGGCATACCATC |
| Sl-ACS6-peak-F | ATGACAAGGTCACGGAACCG |
| Sl-ACS6-peak-R | CGTTTTAGGTAGAGTTGCC |
| Sl-PDIL1-peak-F | CAGCGGAAATTTTAAGCCAG |
| Sl-PDIL1-peak-R | GCATCCTTGGATGATTTAATTTC |
| Ta-RCA-peak-F | ATGGCTGCTGCCTTCTCCTC |
| Ta-RCA-peak-R | GCAAGACCCTTCCACTTGTC |
| Ta-PsbA-peak-F | GTAGATATTGATGGTATTCG |
| Ta-PsbA-peak-R | TAAGAAGTGTAGAACAATTAG |
| Ta-ACS6-peak-F | CTCTTCTACTTCCTCCAGCTC |
| Ta-ACS6-peak-R | GGGTGGTAGGGGTCGTCGGCG |
| Ta-PDIL1-peak-F | CCTCATTCAAGACAGTGACTC |
| Ta-PDIL1-peak-R | CTAGCTTCTTGCAGTGTCCGCAC |

**Table S13 Primers used for RIP-qPCR.**

| **Name** | **Primer (5'-3')** |
| --- | --- |
| RCA-RIP-F | GGCTTGACGCTTGCTATTAATT |
| RCA-RIP-R | AACAAAAACTTGTCATGCCCAG |
| PsbA-RIP-F | TTCCAGGCAGAGCATAACATC |
| PsbA-RIP-R | GATTCGTTTTCAGTGGTTTCCC |
| OsACS6-RIP-F | ATAATGAAAGCATCGTGACAGC |
| OsACS6-RIP-R | TCTTGCGCAATCTCTCTCTATT |
| PDIL1-RIP-F | CGCTGATAACGTCCATGATTTT |
| PDIL1-RIP-R | CTTAGCGATGACAACATCCTTG |

**Table S14 Primers used for relative luciferase activity.**

| **Name** | **Primer (5'-3')** |
| --- | --- |
| RCA-LUC-HindⅢ-F | GAGGTCGACGGTATCGATAAGCTTATGAACATCGCCGACAACC |
| RCA-LUC-BamHI-R | GCCGCTCTAGAACTAGTGGATCCAAAGGTGTAAAGGCAGCTGCCG |
| PsbA-LUC-HindⅢ-F | GAGGTCGACGGTATCGATAAGCTTATGATCCCTACCTTATTGAC |
| PsbA-LUC-BamHI-R | GCCGCTCTAGAACTAGTGGATCCAGCAGCCAAGAAGAAGTGT |
| OsACS6-LUC-HindⅢ-F | GAGGTCGACGGTATCGATAAGCTTGCAGAGGTTGTTGATGATCTAG |
| OsACS6-LUC-BamHI-R | GCCGCTCTAGAACTAGTGGATCCATTGACCTTTGCCTCCTCCAAT |
| PDIL1-LUC-HindⅢ-F | GAGGTCGACGGTATCGATAAGCTTAGTCCAAGAAATTTTTGAAGG |
| PDIL1-LUC-BamHI-R | GCCGCTCTAGAACTAGTGGATCCGACCAGCGGTTTCCTTGTTCTTC |

**Table S15 Primers used in RNA decay rate assays.**

| **Name** | **Primer (5'-3')** | **Usage** |
| --- | --- | --- |
| LOC_Os10g21190.1-F | TCCGTTACCTTTTGGGAGGC | **RNA stability assays** |
| LOC_Os10g21190.1-R | TCCCAGGAAAAGCTCGAACG |  |
| EVM0009308-F | TCGAGCACAGGTTGAGGTTC |  |
| EVM0009308-R | ATGGTACGATCCCTCCGTCA |  |
| EVM0035112-F | TGTGGCCGCTCATGGTTATT |  |
| EVM0035112-R | AACGCGACCTTGGCTATCAA |  |
| LOC_Os11g29140.1-F | GTGATGATTGTGATTGCGGTAA |  |
| LOC_Os11g29140.1-R | GCTCAGGCTAGAGAATAACACT |  |
| EVM0015885-F | GTGCCTACCCATAAGCGAGATG |  |
| EVM0015885-R | ATGACTACTGGCAGGATCAACC |  |
| EVM0012725-F | GAAGTCGGCAAAACGGATCCG |  |
| EVM0012725-R | CCTTGTCCGTACCAGTTCTGAG |  |

**References:**

1. Matsumoto, T. et al. The *Nipponbare* genome and the next-generation of rice genomics research in Japan. *Rice (N.Y.)* 2016; **9**: 33.
2. Wang, Y. et al. Time-ordering *japonica*/geng genomes analysis indicates the importance of large structural variants in rice breeding. *Plant Biotechnol. J.* 2023; **21**: 202-218.
3. Sun, X. et al. Reducing ammonia volatilization from paddy field with rice straw derived biochar. *Sci. Total Environ.* 2019; **660**: 512-518.
4. Majumder, S., Datta, K. & Datta, S. K. Agrobacterium tumefaciens-Mediated Transformation of Rice by Hygromycin Phosphotransferase (hptII) Gene Containing CRISPR/Cas9 Vector. *Methods Mol. Bio. (Clifton, N.J.)* 2021; **2238**: 69-79.
5. Wang, Y. et al. Simultaneous editing of three homoeoalleles in hexaploid bread wheat confers heritable resistance to powdery mildew. *Nat. Biotechnol.* 2014; **32**: 947-951.
6. Coluccio Leskow, C. et al. The cytosolic invertase NI6 affects vegetative growth, flowering, fruit set, and yield in tomato. *J. Exp. Bot.* 2021; **72**: 2525-2543.
7. Liang, Z., Brown, R. C., Fletcher, J. C. & Opsahl-Sorteberg, H. G. Calpain-Mediated Positional Information Directs Cell Wall Orientation to Sustain Plant Stem Cell Activity, Growth and Development. *Plant Cell Physiol.* 2015; **56**, 1855-1866.
8. Yu, Q. et al. RNA demethylation increases the yield and biomass of rice and potato plants in field trials. *Nat. Biotechnol.* 2021; **39**, 1581-1588.
9. Liu, E. et al. Favorable Alleles of GRAIN-FILLING RATE1 Increase the Grain-Filling Rate and Yield of Rice. *Plant Physiol.* 2019; **181**: 1207-1222.
10. Wei, S. et al. A transcriptional regulator that boosts grain yields and shortens the growth duration of rice. *Science* 2022; **377**: eabi8455.
11. Hu, B. et al. Variation in NRT1.1B contributes to nitrate-use divergence between rice subspecies. *Nat. Genet.* 2015; **47**: 834-838.
12. Moll, R. H., Kamprath, E. J. & Jackson, W. A. Analysis and Interpretation of Factors Which Contribute to Efficiency of Nitrogen Utilization. *Agron. J.* 1962; **74**: 562-564.
13. Zhang, Y. et al. A highly efficient rice green tissue protoplast system for transient gene expression and studying light/chloroplast-related processes. *Plant Methods* 2011; **7**: 30.
14. Cui, X. et al. 5-Methylcytosine RNA Methylation in Arabidopsis Thaliana. *Mol. Plant* 2017; **10**: 1387-1399.
15. Wang, X. et al. Structural basis of N(6)-adenosine methylation by the METTL3-METTL14 complex. *Nature* 2016; **534**: 575-578.
16. Yang, X. et al. 5-methylcytosine promotes mRNA export - NSUN2 as the methyltransferase and ALYREF as an m(5)C reader. *Cell Res.* 2017; **27**: 606-625.
17. Xu, W. et al. A design optimized prime editor with expanded scope and capability in plants. *Nat. Plants* 2022; **8**: 45-52.
18. Dominissini, D. et al. Transcriptome-wide mapping of N(6)-methyladenosine by m(6)A-seq based on immunocapturing and massively parallel sequencing. *Nat. Protoc.* 2013; **8**: 176-189.
19. Zhang, Q. et al. N(6)-Methyladenine DNA Methylation in *Japonica* and *Indica* Rice Genomes and Its Association with Gene Expression, Plant Development, and Stress Responses. *Mol.* *Plant* 2018; **11**: 1492-1508.
20. Zhang, Y. et al. Model-based analysis of ChIP-Seq (MACS). *Genome Biol.* 2008; **9**: R137.
21. Machanick, P. & Bailey, T. L. MEME-ChIP: motif analysis of large DNA datasets. *Bioinformatics (Oxford, England)* 2011; **27**: 1696-1697.
22. Trapnell, C. et al. Transcript assembly and quantification by RNA-Seq reveals unannotated transcripts and isoform switching during cell differentiation. *Nat. Biotechnol*. 2010; **28**: 511-515.
23. Anders, S., Pyl, P. T. & Huber, W. HTSeq--a Python framework to work with high-throughput sequencing data. *Bioinformatics (Oxford, England)* 2015; **31**: 166-169.
24. Wiśniewski, J. R., Zougman, A., Nagaraj, N. & Mann, M. Universal sample preparation method for proteome analysis. *Nat. Methods* 2009; **6**: 359-362.
25. Liu, Y. et al. mRNA m^5^C controls adipogenesis by promoting CDKN1A mRNA export and translation. *RNA Biol.* 2021; **18**: 711-721.
26. Zhang, K. et al. A common wild rice-derived BOC1 allele reduces callus browning in indica rice transformation. *Nat. Commun.* 2020; **11**: 443.
